# Study on the Influence of Protector Design on the Biomechanical Characteristics of Knee Joint Movement

**DOI:** 10.3390/s26072168

**Published:** 2026-03-31

**Authors:** Jiaxin Zhao, Xupeng Wang, Lingxiao Xi, Xinran Cheng, Jihyun Bae, Yongwei Li

**Affiliations:** 1 School of Mechanical Engineering, Xi’an University of Technology, Xi’an 710048, China; zhaojiaxin@stu.xaut.edu.cn (J.Z.); xilingxiao0318@163.com (L.X.); chengshier12@gmail.com (X.C.); 2 School of Art and Design, Xi’an University of Technology, Xi’an 710054, China; 3 Human-Tech Convergence Program, Department of Clothing & Textiles, Hanyang University, Seoul 04763, Republic of Korea; pollysammi@hanyang.ac.kr

**Keywords:** knee protector design, knee joint, sports biomechanics, muscle activity

## Abstract

To investigate how knee joint protector design affects the biomechanical characteristics of knee motion under various activities, this pilot study (*n* = 5) examined how knee joint protector design modulates knee biomechanics across walking, jogging, squatting, and sit-to-stand tasks using optical motion capture and AnyBody musculoskeletal modeling (FullBody_GRFPrediction). We quantified knee flexion kinematics, model-estimated joint reaction forces and moments, and model-estimated muscle activity of eight lower-limb muscles under four conditions with different levels of structural constraint: no protector (Pro.off), a conventional sleeve-type protector (Pro.a), a segmented support protector (Pro.b), and a wrapping fixation protector (Pro.c). The biomechanical protective performance of the knee joint protector was task- and phase-dependent. The results showed that Pro.a optimized muscle activation. Pro.b increased sagittal-plane design but increased joint loading and muscle activity. Pro.c induced noticeable distal compensation along the kinetic chain. The findings revealed that protector effects were task-dependent. Dynamic tasks mainly affected coronal-plane stability parameters, whereas quasi-static tasks more clearly altered sagittal load distribution. In this study, biomechanical protective performance is defined as reduced knee joint loading without disproportionate increases in model-estimated muscle activity or excessive loss of functional knee flexion range. Under this definition, greater structural constraint did not consistently produce a more favorable biomechanical profile. These results provide a feasibility baseline for task-specific protector evaluation and motivate confirmatory studies with larger cohorts and experimental validation. This study provides theoretical and methodological insights to guide future design and optimization of knee joint protectors.

## 1. Introduction

The knee joint, the largest and most complex load-bearing joint in the human body, endures substantial mechanical stress during both daily and athletic activities [1]. As a widely used protective device, knee joint protectors play a crucial role in injury prevention, postoperative rehabilitation, and the management of chronic joint disorders [2]. By altering the mechanical environment of the knee joint, the knee joint protector can enhance joint stability, thereby reducing pain and slowing down the progression of degenerative changes [3]. In biomechanical research, protectors are evaluated using surrogate indicators (such as kinematics and joint torques) that cannot directly quantify tissue-level stress or the clinical risk of injury.

In recent years, research on knee joint protectors has evolved from traditional structural design to a multidisciplinary framework that integrates biomechanical assessment. Previous studies have confirmed that knee joint protectors can effectively reduce joint loading [4]. However, most investigations have focused on simplified or static movements and have not adequately addressed dynamic or functionally relevant activities [5]. Within biomechanics, current efforts aim to optimize muscle activation patterns and minimize pathological co-contraction indices [6]. In parallel, advances have been made in the development of smart knee joint protectors that incorporate sensor technologies and real-time feedback systems. Interestingly, psychological studies suggest that placebo effects may account for 30–40% of the perceived benefits of protector use [7]. Nonetheless, these innovations remain largely at the proof-of-concept stage, and significant challenges persist before their widespread clinical implementation [8].

With advances in knee joint protector biomechanics, musculoskeletal modeling has emerged as a powerful tool for motion analysis and biomechanical evaluation. The AnyBody Modeling System enables comprehensive analysis of joint kinematics, kinetics, and muscle activation based on motion capture data [9,10,11]. Through musculoskeletal simulation, it is possible to assess how different knee joint protector conditions influence knee mechanics across various movement patterns without relying on expensive experimental setups. For example, simulation studies on knee osteoarthritis protectors have identified three primary mechanisms by which protectors reduce medial joint loading [12]. To enhance simulation accuracy, other researchers have integrated multi-body modeling to evaluate novel offloading protector designs [13] and have optimized cartilage representation within musculoskeletal frameworks [14]. Musculoskeletal modeling provides a structured way to estimate internal loading from measured kinematics and assumed or estimated external loads. It complements experimental validation and should be interpreted in light of its modeling assumptions.

Despite the growing body of research, systematic biomechanical assessments of knee joint protectors remain limited, leading to ongoing debate regarding their mechanisms and practical efficacy. When combined with individualized motion-capture data, the AnyBody platform can estimate joint angles, reaction forces, moments, and muscle activity. These outputs provide a biomechanical basis for the evaluation and design optimization of protectors [15,16].

This pilot study compared the biomechanical effects of three knee joint protector designs across walking, jogging, squatting, and STS tasks. The results showed that protector effects were task- and phase-dependent. In several tasks, the rigid-support designs (Pro.b and Pro.c) were associated with larger deviations in model-estimated joint loading and muscle activity, whereas Pro.a showed smaller deviations from the unbraced condition in selected variables. Overall, greater structural constraint did not consistently produce a more favorable biomechanical profile. These findings provide a descriptive baseline for task-specific protector evaluation and support future studies with larger cohorts and experimental validation.

## 2. Methods

### 2.1. Research Object

Five healthy young adults (three males and two females) participated in this study. Their mean age was 25.8 ± 2.6 years (males: 27.0 ± 2.6 years, females: 24.0 ± 1.4 years), mean height was 172.8 ± 5.6 cm (males: 176.3 ± 3.2 cm, females: 167.5 ± 3.5 cm), mean body mass was 64.8 ± 7.8 kg (males: 69.7 ± 5.5 kg, females: 57.5 ± 2.1 kg), and mean BMI was 21.7 ± 2.2 kg/m^2^ (males: 22.5 ± 2.4 kg/m^2^, females: 20.5 ± 1.6 kg/m^2^). None of the participants had prior experience wearing knee joint protectors during exercise. All were right-leg dominant, with no history of knee injury, surgery, or degenerative joint diseases such as osteoarthritis. Radiographic examinations confirmed normal knee anatomy. Participants were instructed to avoid strenuous exercise for 24 h before testing. The experimental procedures conformed to the Declaration of Helsinki and were approved by the Ethics Committee of Xi’an University of Technology. Written informed consent was obtained from all participants before data collection.

### 2.2. Experimental Equipment

**Figure 1:**
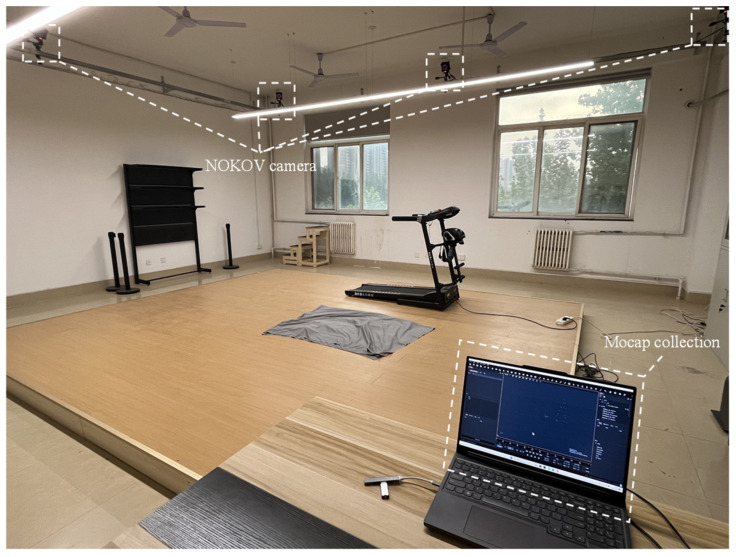
Experimental Site.

All data collection and testing were conducted at the Sports Biomechanics Laboratory of Xi’an University of Technology (Figure 1). An NOKOV optical 3D motion capture system (Beijing Nokov Science & Technology Co., Beijing, China), including Mars infrared cameras, 12 mm reflective markers, and XINGYING 3.2 software, was used. The system operated at a sampling frequency of 240 Hz, with a calibrated three-dimensional spatial accuracy of ±0.2 mm. This specification reflects camera calibration accuracy. Skin-marker-based human motion capture remains subject to soft tissue artifact (STA), and this source of error may be further aggravated by external devices. Musculoskeletal simulation and analysis were performed using the AnyBody Modeling System 7.4 (AnyBody Technology, Aalborg, Denmark).

Marker placement under the protector. For the protector conditions, markers located in the knee region were placed on the external surface of the protector to maintain optical visibility, and their positions were aligned to palpated anatomical landmarks before each trial. The same trained operator performed marker placement across all conditions. Marker positions were visually checked between trials, and markers were re-attached if displacement was observed. Because this procedure may introduce marker displacement due to knee joint protectors in addition to STA, kinematic differences are reported descriptively and interpreted with this limitation in mind.

### 2.3. Experimental Design

**Figure 2:**
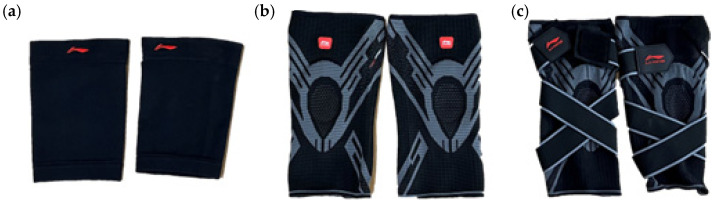
Three knee joint protectors with different design levels: (**a**) Conventional sleeve-type protector, (**b**) Zonal knitted protector, (**c**) Wrap-type strapping protector.

This study investigated knee biomechanics under four conditions: no protector (Pro.off) and three knee protectors of different designs. The three protectors, all from the same manufacturer, were shown in Figure 2.

The first protector (Pro.a) is a conventional sleeve-type design produced through a homogeneous knitting process. Its fabric structure is uniform, with no regional differentiation, providing fundamental support and compression primarily through the intrinsic elasticity of the knitted material.

The second protector (Pro.b) is an improved version featuring zonal differential knitting, which offers varying elastic strengths across regions to meet the localized requirements of the knee for pressure and support. Additionally, rigid support strips are incorporated along both sides of the sagittal plane to enhance lateral stability and restrict excessive movement.

The third protector (Pro.c) builds upon the previous design by introducing a wrapping strap configuration. This adjustable strap provides circumferential fixation during wear, improving overall stability and enabling precise control of joint compression by adjusting strap tension.

The structural differences among these three protectors were treated as key experimental variables to evaluate how protector design affects the biomechanical characteristics of knee motion under multiple movement conditions.

Reflective markers were placed according to the anatomical landmarks defined in the standard lower-limb model of the AnyBody Modeling System and following the CGM body marker set protocol. A total of 22 markers were attached to each participant at the following positions: suprasternal notch, sternal midpoint, spinous processes of the seventh cervical and tenth thoracic vertebrae, bilateral anterior superior iliac spines (ASIS), posterior superior iliac spines (PSIS), lateral thighs, lateral femoral epicondyles, lateral shanks, fifth metatarsal heads, toes, lateral malleoli, and heels.

Each participant performed tests under four protector conditions: no protector (Pro.off), conventional sleeve protector (Pro.a), segmented support protector (Pro.b), and wrapping fixation protector (Pro.c). The wearing sequence was randomized using a Latin square design to minimize order effects. After putting on each protector, participants completed a 5 min familiarization session to ensure natural and comfortable movement. As this is a pilot study, the 5 min familiarization may have been insufficient for full adaptation to the knee joint protector condition. Longer adaptation periods will therefore be used in confirmatory work to distinguish transient strategy adjustments from more stable movement patterns.

Once all markers were affixed and the protector was properly worn, participants stood quietly for 10 s in an upright posture with arms relaxed at their sides. Upon hearing the “start” command, they performed the designated movement tasks. Four motion tasks were included: walking, jogging, squatting, and sit-to-stand (STS). All trials were conducted under identical environmental conditions, and task order was randomized to avoid fatigue and learning effects.

**Figure 3:**
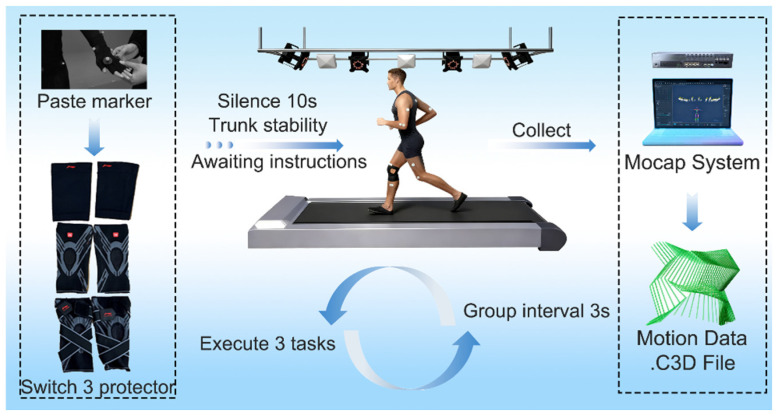
Experimental flowchart. Arrows indicate the sequence of processing and information flow.

The right knee joint was used as the reference for all biomechanical analyses to standardize within-subject comparisons across conditions. Bilateral asymmetry effects are addressed as a limitation and will be examined in future studies. Motion data were recorded using the Mocop system, and each task was repeated five times. The three trials with smooth trajectories and no visible artifacts were selected for analysis. After preprocessing, data were exported and saved in .c3d format. The experimental procedure was illustrated in Figure 3.

- Walking

Walking is one of the most commonly analyzed motion states in kinematic studies. Before performing kinematic analysis, it is essential to define the gait cycle clearly. A complete gait cycle was defined as the interval between two successive identical events of the same foot. Typically, the gait cycle includes four fundamental events and two primary phases. The stance phase accounts for approximately 60% of the cycle, and the swing phase accounts for about 40% [17]. In this study, the gait cycle (including both walking and jogging) was defined as beginning and ending with the right foot’s heel strike.

The heel strike (HS) was defined as the first gait event, marking the onset of the walking cycle at 0% of the gait period. This is followed by midstance, during which the body’s center of mass gradually shifts over the right foot. At this moment, the right leg provides full body support, joint motion is relatively stable, and the ankle begins to dorsiflex in preparation for propulsion. This event occurs at approximately 30% of the gait cycle.

Subsequently, the right toes leave the ground, indicating the transition from the stance phase to the swing phase. Propulsive force is primarily generated by the forefoot, driving the body forward. This event corresponds to toe-off (TO), which occurs at approximately 60% of the gait cycle.

Thereafter, the right leg enters mid-swing, during which the knee flexes to its maximum angle to shorten the effective limb length, preventing foot–ground interference while the shank advances forward. This represents midswing, occurring at approximately 80% of the gait cycle.

**Figure 4:**
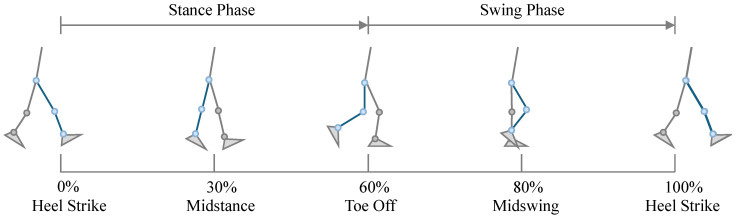
Definition of the walking cycle. The blue lower limb denotes the right leg.

Finally, the heel contacts the ground again (heel strike), ending the swing phase and beginning a new stance phase. The right foot once again initiates contact with the ground through heel strike, completing one full gait cycle, which corresponds to approximately 100% of the cycle, as illustrated in Figure 4.

b. Jogging

The jogging cycle is similar to the conventional walking gait cycle and can also be divided into a stance phase and a swing phase. However, unlike walking, jogging contains a distinct flight phase in which neither foot is in contact with the ground. Overall, the relative duration of the stance phase decreases during jogging, while the proportions of the swing phase and flight phase increase. The stance phase accounts for approximately 40% of the entire jogging cycle, whereas the swing phase plus flight phase together represent about 60% of the cycle [18].

After right heel contact, the jogging cycle includes three early gait events: heel strike (HS), midstance, and toe-off (TO). These events occur at approximately 0%, 20%, and 40% of the cycle, respectively. When the right leg completes the latter portion of the swing phase, and the left foot has not yet made ground contact, a brief flight phase occurs in which both feet are off the ground. This flight phase, occurring around 60% of the jogging cycle, is the key distinction between jogging and walking.

**Figure 5:**
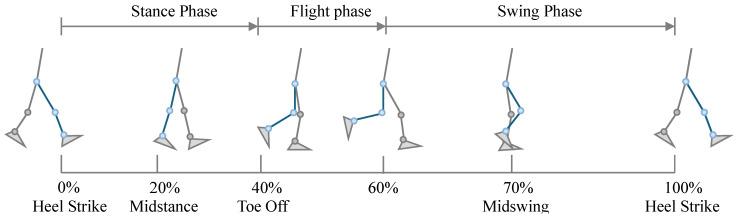
Definition of the jogging cycle. The blue lower limb denotes the right leg.

Subsequently, the right leg reaches midswing at approximately 70% of the cycle, after which the heel strikes the ground again (heel strike) at about 100%, completing one full jogging cycle, as illustrated in Figure 5.

c. Squatting

The squatting motion differs from the cyclic movements of walking and jogging, as it is a non-periodic motion primarily characterized by displacement along the vertical (z) axis. A complete squat–stand action consists of a descending phase and an ascending phase. In this study, only the descending phase was analyzed. Because the movement cannot start and end with the same posture, it is defined as a half-cycle motion.

**Figure 6:**
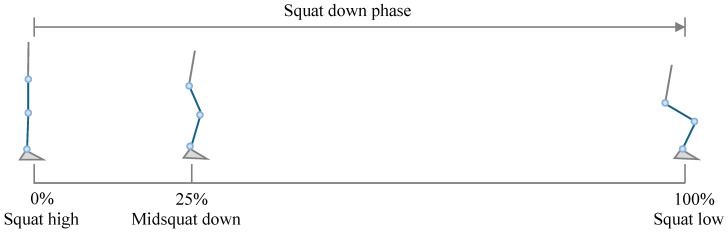
Definition of the squatting cycle. The blue lower limb denotes the right leg.

The squat started from the upright standing position, which was defined as the squat high point (0%). At this point, the knee and hip joints were nearly extended, and the center of mass was at its highest position. As the knees and hips flex, the center of mass gradually lowers, and the quadriceps contract eccentrically to control the rate of descent. This stage corresponds to the midsquat down position, occurring at approximately 25% of the cycle. When the maximum squat depth is reached, the motion reaches the squat low point (100%) [19], as shown in Figure 6. Therefore, in this study, the squatting cycle was defined as starting at the squat high point and ending at the squat low point. Experimental observations indicated that participants typically performed a three-quarter squat (¾-squat), consistent with the common movement depth observed in daily activities. Consequently, the ¾-squat was adopted as the target squatting motion.

d. STS

The sit-to-stand (STS) movement is one of the most common functional motions in daily life, representing the continuous process of rising from a seated to an upright posture. This movement can be described using either a classical four-phase model or a simplified three-phase model. The two models are not contradictory but rather differ in analytical focus. The three-phase model provides a more integrated perspective and is particularly useful for assistive device design and functional assessment in daily activities. Therefore, it was adopted in this study [20].

The motion begins from the seated position (seat-on), during which the participant maintains a stable sitting posture with the hips in contact with the chair, and body weight primarily supported by the seat. This point corresponds to 0% of the cycle. When the hips completely lift off the seat, body support shifts entirely to the lower limbs. This seat-off event occurs at approximately 30% of the cycle and marks a key phase of STS. After the seat-off, both the knee and hip joints rapidly extend, and the trunk gradually straightens while the center of mass continues to rise. This corresponds to approximately 70% of the movement cycle. Finally, upon reaching the fully extended posture, the center of mass stabilizes between the two feet, the knees are fully extended, and the movement cycle concludes at 100%.

**Figure 7:**
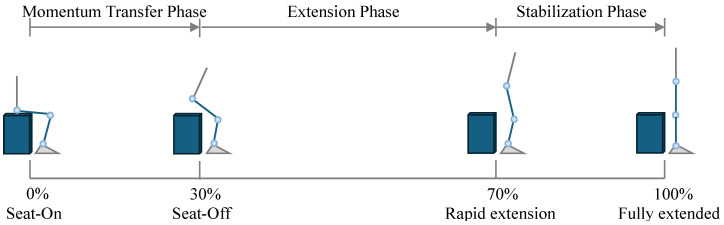
Definition of the STS cycle. The blue lower limb denotes the right leg, and the dark blue cuboid denotes the experimental seat.

Similar to the squatting task, the STS movement was non-cyclic and typically defined as a single, complete transition from sitting to standing. Accordingly, in this study, the STS cycle was defined as beginning with the seated (seat-on) position and ending with the fully extended standing position, as illustrated in Figure 7.

## 3. Results

### 3.1. Data Processing

**Figure 8:**
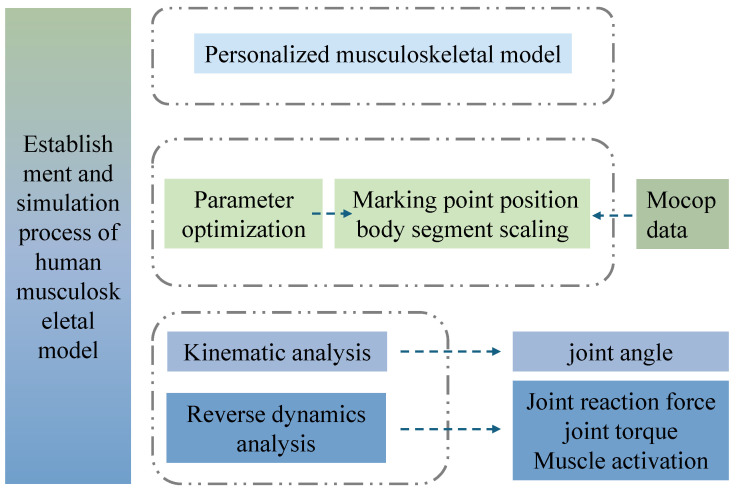
Workflow of the data-processing and musculoskeletal-modeling pipeline. Green boxes denote experimental motion-capture input and parameter-optimization steps, whereas blue boxes denote the personalized musculoskeletal model, kinematic and inverse-dynamics analyses, and the corresponding biomechanical outputs. Dashed outlines indicate grouped workflow modules, and dashed arrows indicate the direction of data flow and computational processing.

A total of 3360 simulation data sets were generated and processed according to the workflow shown in Figure 8. First, an individualized musculoskeletal model was established using the FullBody_GRFPrediction model in the AnyBody Modeling System. Marker trajectories were low-pass filtered (4th-order, zero-lag Butterworth, 6 Hz for walking/jogging and 10 Hz for squatting/STS) before inverse dynamics. The collected motion capture data, along with each participant’s height and weight, were used as input parameters for model optimization. The least-squares method was applied to minimize the spatial distance errors between the predefined virtual markers and the experimental skin markers. By fitting the spatial parameters of bony landmarks, the geometric dimensions of the skeletal model were determined, and segmental scaling of the skeletal system was completed [21].

Subsequently, a two-step calibration method was employed to adjust the muscle groups and calculate muscle dimensions [22], and muscle recruitment used the default AnyBody polynomial criterion (*p* = 3). Each trial was time-normalized to 0–100% of the defined cycle, and three artifact-free repetitions were averaged per participant. Group waveforms are reported as mean ± SD across participants (*n* = 5). Joint reaction forces, joint moments, and muscle activity are model-estimated outputs and are interpreted within the assumptions of the AnyBody workflow.

Model-fitting quality was quantified as the maximum marker residual error (mm) reported by the optimization routine. The parameter optimization results indicated successful convergence for all four motion tasks, both with and without knee joint protectors. The optimization accuracy was consistently below 0.05 mm, suggesting a satisfactory level of optimization. This reflects high precision in the matching of bony landmarks, validating the model for subsequent simulation analysis.

### 3.2. Kinematic Analysis

**Figure 9:**
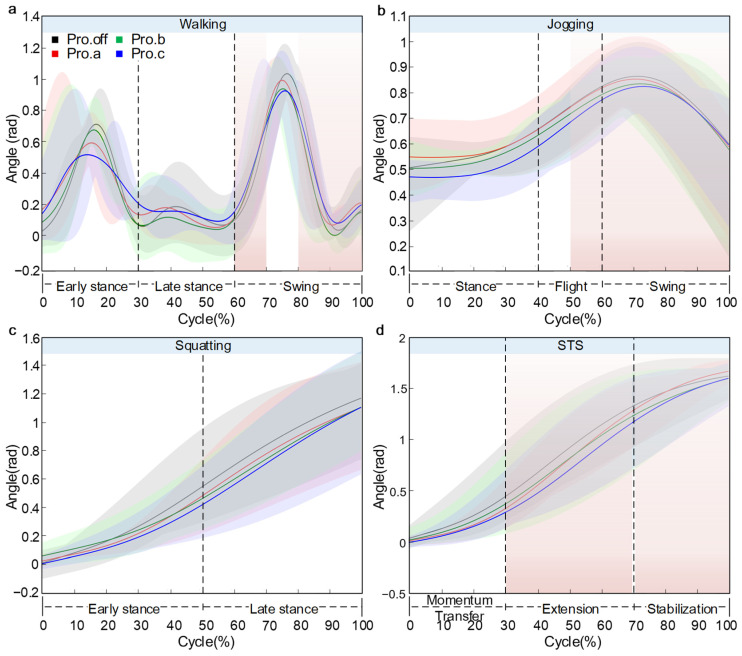
Knee flexion angle over the normalized cycle: (**a**) walking, (**b**) jogging, (**c**) squatting, (**d**) STS. Solid line: mean, shaded area: ±SD across participants (*n* = 5).

As shown in Figure 9, the variation in knee flexion angle across the four motion tasks was illustrated. The results indicated that wearing knee joint protectors altered knee flexion patterns. The main effects were reduced range of motion and condition-specific differences near peak flexion. Given bracing-related marker displacement and the pilot sample size, these differences are reported descriptively, with uncertainty shown by ±SD shading.

During walking, participants were tested on a treadmill at 3.0 km/h. The knee flexion angle exhibited a typical bimodal pattern. The first peak occurred at approximately 16% of the gait cycle, with flexion angles of 0.70 rad, 0.58 rad, 0.65 rad, and 0.52 rad for the Pro.off, Pro.a, Pro.b, and Pro.c conditions, respectively. The greatest flexion was observed in the Pro.off condition, while the lowest was in Pro.c, yielding a peak difference of 0.18 rad (25.7%). The second peak appeared at around 75% of the cycle, reaching values between 0.85 rad and 1.04 rad, with slightly higher peaks observed in the Pro.off and Pro.a conditions. A trough of approximately 0.10 rad was found midway between the two peaks, at around 55% of the cycle, where all four curves closely overlapped. The flexion angle at both the start and end of the cycle was approximately 0.20 rad.

During jogging, participants were tested on a treadmill at 6.0 km/h. The knee flexion angle displayed a unimodal profile. Starting at approximately 0.5 rad, the angle increased progressively, reaching its peak at around 70% of the gait cycle. The highest peak was observed in the Pro.off condition at about 0.86 rad, followed by Pro.a and Pro.b, both at approximately 0.84 rad, while Pro.c exhibited the lowest peak at around 0.82 rad, resulting in a maximum difference of 0.04 rad (4.7%). Following the peak, the flexion angle rapidly decreased, reaching roughly 0.59 rad at the end of the cycle. Overall, the curves shared a similar shape, with noticeable divergence observed in the peak region.

During the squatting motion, the knee flexion angle exhibited a smooth, monotonically increasing curve. As the movement progressed, the knee continued to flex, approaching maximum flexion toward the end of the motion. The initial value was close to 0 rad and followed a logarithmic growth pattern. By the end of the motion, the knee flexion angle reached approximately 1.15 rad, 1.14 rad, 1.14 rad, and 1.13 rad in the Pro.off, Pro.a, Pro.b, and Pro.c conditions, respectively. The highest value was observed in the Pro.off condition, while Pro.c exhibited the lowest value, resulting in a difference of 0.02 rad (1.7%). The curves overlapped closely during the first half of the motion but gradually diverged in the latter half, with Pro.c showing a distinct deviation from the other three conditions. The SD band(mean ± SD across participants) widened toward the end, particularly for Pro.c.

During the STS movement, the knee flexion angle increased gradually. The flexion angle rose slowly at the beginning, followed by a rapid increasing phase between 20% and 70% of the cycle, and then tended to plateau. The Pro.a condition exhibited the greatest peak flexion of approximately 1.70 rad. In comparison, Pro.off and Pro.b reached around 1.65 rad, and Pro.c showed a noticeably lower value of about 1.62 rad, with an inter-condition difference of 0.08 rad (4.7%). The divergence among the curves began at approximately 40% of the cycle and widened thereafter.

### 3.3. Kinetic Analysis

**Figure 10:**
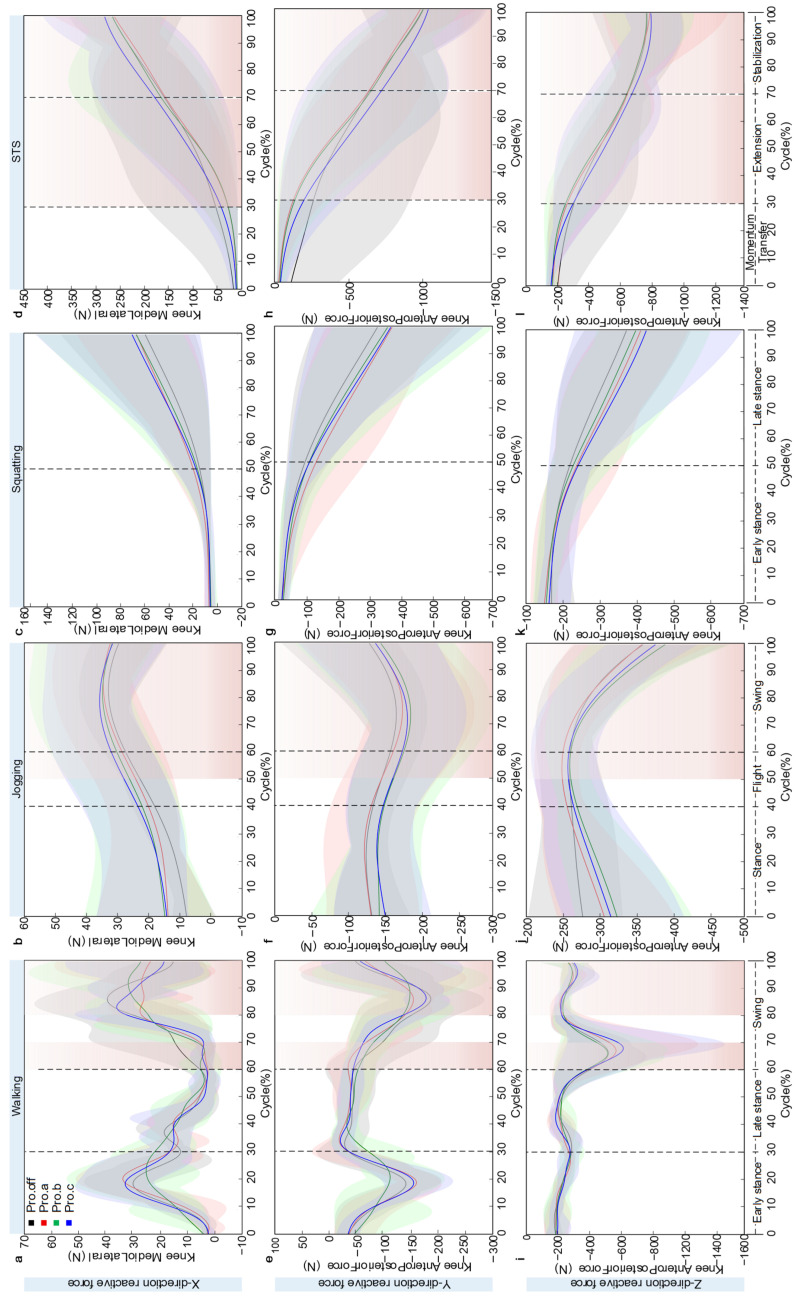
Knee joint reaction force diagram over the normalized cycle: Panels (**a**–**d**) respectively show the mediolateral component during walking, jogging, squatting, and STS. Panels (**e**–**h**) respectively show the anteroposterior component during walking, jogging, squatting, and STS. Panels (**i**–**l**) respectively show the ProximoDistal component during walking, jogging, squatting, and STS. Solid line: mean, shaded area: ±SD across participants (*n* = 5).

During walking, the variations in knee joint reaction force were illustrated in Figure 10. Across a full gait cycle, the knee joint reaction force exhibited a clear periodic fluctuation pattern, with distinct differences among protector conditions in force characteristics along each directional axis. In the mediolateral (X) direction, the reaction force displayed a pronounced double-peaked pattern. The first peak occurred during the initial contact (heel strike) phase, with peak magnitudes of approximately 28.5 N, 32.15 N, 24.50 N, and 31.15 N for the Pro.off, Pro.a, Pro.b, and Pro.c conditions, respectively. The second peak appeared around the midswing phase, reaching 39.02 N, 28.05 N, 33.30 N, and 35.12 N, respectively. The intermediate valley between the two peaks was close to zero in all cases. The Pro.a showed the highest value at the first peak. In the anteroposterior (Y) direction, the reaction force exhibited a negative double-peaked pattern, with the first negative peak reaching approximately −160 N and the second ranging between −150 N and −190 N. The four curves showed a high degree of overlap, except for Pro.b, which diverged slightly at the second peak. The Pro.off and Pro.a conditions demonstrated relatively greater negative values. In the vertical (Z) direction, the reaction force followed a single-peak loading pattern, with the peak occurring near 70% of the gait cycle. The maximum values for the four conditions were approximately −520 N, −590 N, −520 N, and −650 N, respectively. The curves remained highly consistent throughout the gait cycle, with both the start and end values stabilized around −200 N. The SD band slightly widened near the peak but remained narrow overall.

During jogging, the tri-axial joint reaction forces exhibited loading characteristics distinct from those observed during walking, reflecting the biomechanical effects of knee joint protectors under higher dynamic conditions. Although the overall trends of the reaction force curves were consistent across all protector conditions, notable differences were found in peak magnitudes and phase characteristics. In the mediolateral (X) direction, the reaction force followed a gradually increasing single-peak pattern. Starting at approximately 15 N, the force rose to its peak between 80% and 85% of the cycle. The Pro.b and Pro.c conditions showed higher peak values, approximately 35.12 N and 35.55 N, respectively, whereas Pro.off and Pro.a exhibited lower peaks, around 32.14 N and 35.05 N. The differences among the four are very small in total. Notably, the Pro.b and Pro.c curves remained above those of Pro.off and Pro.a throughout the entire motion cycle, with the difference reaching 3–4 N (9.6%) at the peak. All curves converged to around 30 N toward the end of the cycle. In the anteroposterior (Y) direction, the reaction force demonstrated a single-valley pattern, decreasing gradually from about −150 N at the start to a minimum of approximately −190 N, and then returning to −150 N by the end, forming a symmetric U-shaped curve. The four curves exhibited a high degree of overlap, with maximum differences of less than 10 N, indicating that the protector type had minimal influence on loading in the Y direction. In the vertical (Z) direction, the reaction force displayed a typical single-peak loading pattern, increasing from about −300 N at the start to peak values between −250 N and −260 N. The Pro.b condition reached the maximum peak at approximately −231.53 N, while Pro.off had the lowest peak around −355 N.

During the squatting motion, the tri-axial knee joint reaction forces exhibited quasi-static loading characteristics distinct from those observed in dynamic movements. In the mediolateral (X) direction, the reaction force followed a typical exponential growth pattern. Starting near zero, it increased slowly during the first 0–30% of the motion cycle, then entered an accelerated growth phase. By the end of the movement, the terminal values under the four protector conditions reached approximately 60 N, 65 N, 68 N, and 70 N. The Pro.c condition showed the highest load, about 2.8% greater than Pro.b, while Pro.off exhibited the lowest. The curves overlapped closely during the first 40% of the motion and gradually diverged in the later phase, with the SD band expanding as the movement progressed. In the anteroposterior (Y) direction, the reaction force demonstrated a nearly linear decreasing trend, dropping steadily from an initial value of 0 N. The terminal values were approximately −320 N, −370 N, −360 N, and −365 N. Pro.a showed the most negative value, while Pro.off had the smallest value, with a difference of 50 N (13.5%), representing the largest variation among the three directions. The four curves maintained a highly parallel pattern throughout the movement. In the vertical (Z) direction, the reaction force also followed a linear decreasing trend, starting at approximately −150 N and ending at −380 N, −410 N, −400 N, and −420 N under the four protector conditions, respectively. The Pro.c condition reached the maximum load, while Pro.off had the lowest, resulting in a difference of 40 N (10.5%).

During the STS movement, the tri-axial knee joint reaction forces exhibited distinct patterns of change. In the mediolateral (X) direction, the reaction force exhibited an exponential growth pattern, starting from zero and increasing slowly during the 0–30% phase of the cycle, then accelerating thereafter. The terminal values under the four protector conditions reached approximately 263 N, 254 N, 261 N, and 279 N. The Pro.a condition exhibited the lowest load, while Pro.c showed the highest, with a difference of 25 N (8.9%). In the anteroposterior (Y) direction, the reaction force demonstrated a continuously decreasing trend, dropping linearly from zero to approximately −1000 N at the end of the cycle. The four curves overlapped almost completely, showing negligible differences among protector conditions. In the vertical (Z) direction, the reaction force also showed a monotonic decrease, from about −200 N at the start to approximately −790 N at the end. The Pro.a and Pro.c conditions exhibited slightly lower values than Pro.off and Pro.b, though the differences were minimal, and the four curves remained nearly parallel throughout the movement.

In this study, knee joint reaction forces are reported as tri-axial components. This format highlights direction-specific load redistribution across tasks and protector designs. A single resultant magnitude would obscure these directional changes. Normalized JRF data (BW) are provided in Supplementary Tables S2–S5 to improve cross-participant comparability. The main text retains component-wise interpretation because the primary design is within-subject across protector conditions.

**Figure 11:**
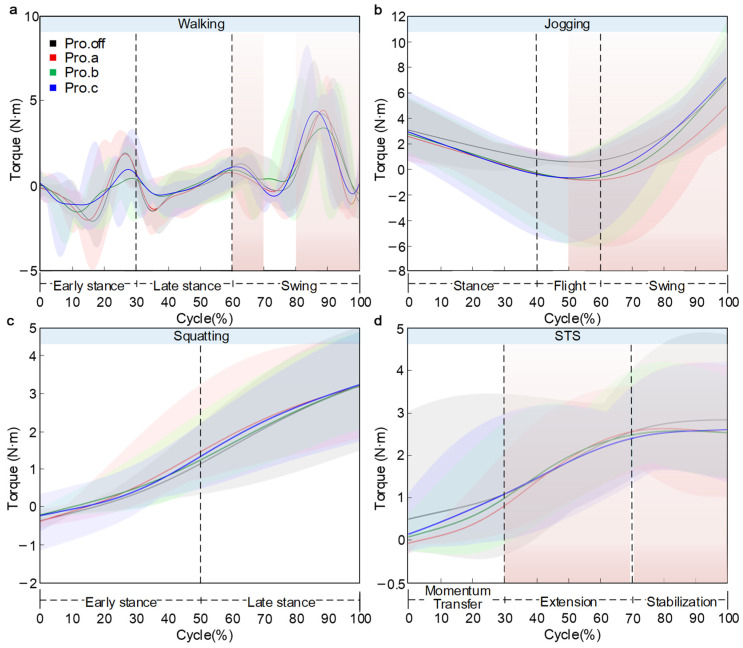
Knee joint torque diagram over the normalized cycle: (**a**) walking, (**b**) jogging, (**c**) squatting, (**d**) STS. Solid line: mean, shaded area: ±SD across participants (*n* = 5).

The variations in knee joint moment were illustrated in Figure 11. Normalized knee flexion moment data (BW × body height) are provided in Supplementary Tables S6–S9. The main text retains a descriptive interpretation of phase-localized differences.

During walking, the knee moment exhibited a clear pattern of periodic fluctuation. Starting at approximately 0.5 N·m, the curve reached its first trough of about −2.0 N·m at around 10–20% of the gait cycle, followed by an increase to a peak of roughly 2.0 N·m at 25–30% of the cycle. A slight decrease occurred near 35%, and another trough of approximately −1.0 N·m appeared around 75% of the cycle. The overall trend of the curve is consistent, with peak differences of less than 1.1 N·m. The key divergence occurred during the final 80–100% of the cycle. The Pro.c condition rose sharply to approximately 4.35 N·m, followed by Pro.off at about 4.0 N·m, while Pro.a and Pro.b reached around 4.76 N·m and 3.58 N·m. The maximum inter-condition difference reached 1.18 N·m (24.7%). The SD bands were narrow during the early phase but widened substantially toward the end, reflecting increased dispersion of the model-estimated moments in the terminal phase.

In the jogging condition, the knee flexion moment exhibited a typical U-shaped curve. Starting at approximately 3.0 N·m, the moment gradually decreased and reached its minimum between 50–60% of the cycle, with trough values of 0.5 N·m, −1.2 N·m, −1.0 N·m, and −0.8 N·m for the Pro.off, Pro.a, Pro.b, and Pro.c conditions. Pro.off showed the highest trough value, whereas Pro.a exhibited the most negative moment. Following the trough, the moment rose sharply, with terminal values of 7.08 N·m, 4.56 N·m, 7.35 N·m, and 6.31 N·m. Pro.off and Pro.c reached the highest end-cycle values, whereas Pro.a was the lowest, yielding a maximum difference of 2.52 N·m. The SD bands were relatively wide around the trough region and toward the end of the cycle.

In the squatting task, the knee flexion moment exhibited a typical nonlinear increasing pattern, with all conditions starting near 0.2 N·m. During the early phase of squatting (0–40%), the moment rose gradually to approximately 0.5 N·m, with a relatively small slope. In the mid-to-late phase (40–100%), the slope increased substantially, and the moment rose sharply. The final torque values across all protective device conditions are relatively consistent, ranging from approximately 3.1–3.4 N·m. Analysis of the SD bands indicated low and comparable dispersion across groups during the first half of the movement (0–50%). In the second half (50–100%), the intervals widened noticeably. Pro.off exhibited the greatest between-subject dispersion (widest SD band), whereas Pro.c showed the smallest dispersion (narrowest SD band).

During the STS task, the knee flexion moment exhibited a pattern of rapid increase followed by stabilization. The moment started at 0.0 N·m under all conditions and rose quickly during the 0–60% phase of the cycle, after which it plateaued. The terminal values were approximately 2.85 N·m, 2.66 N·m, 2.59 N·m, and 2.65 N·m for the Pro.off, Pro.a, Pro.b, and Pro.c conditions. Pro.a showed the highest final moment, while Pro.b presented the lowest, with a maximum difference of 0.26 N·m (9.1%). The curves overlapped closely during the first half of the movement but gradually diverged after around 40% of the cycle, maintaining these differences through the end. The SD band remained relatively stable throughout the entire motion.

Joint moments were obtained directly from inverse dynamics in AnyBody based on the fitted kinematics and predicted external loading in this workflow. Therefore, terminal-phase moment features are interpreted conservatively, and further validation with measured GRF is planned.

**Figure 12:**
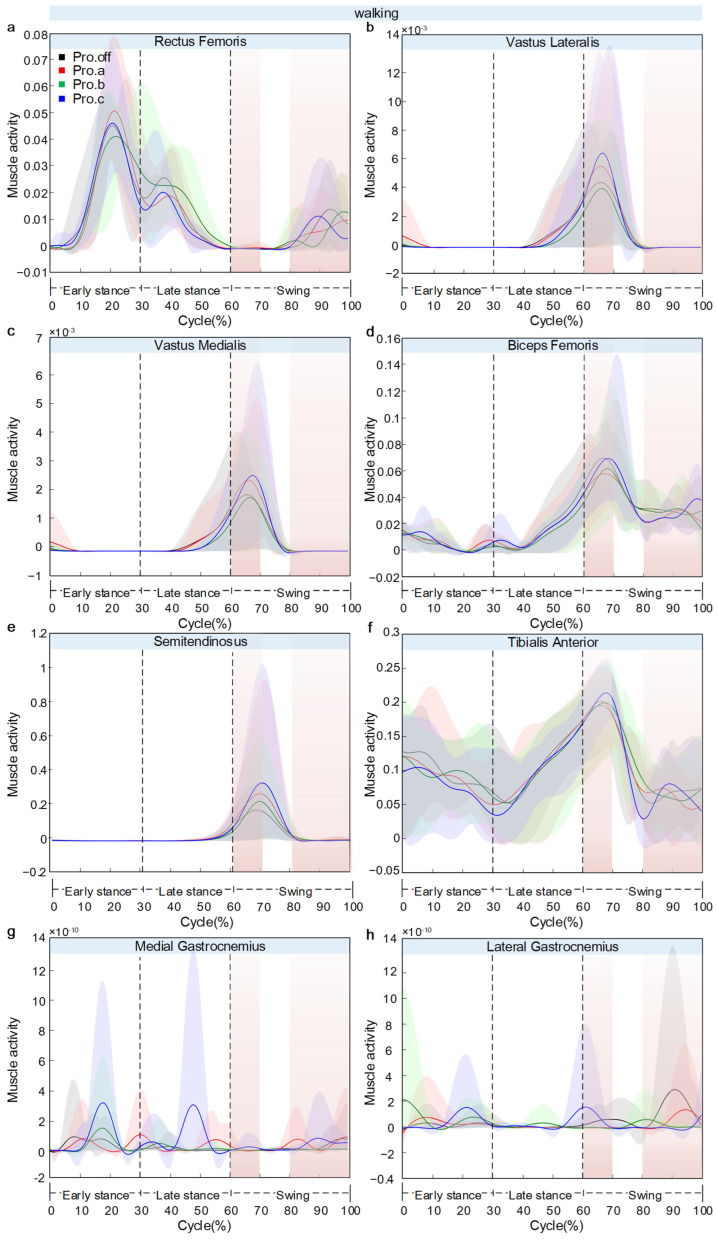

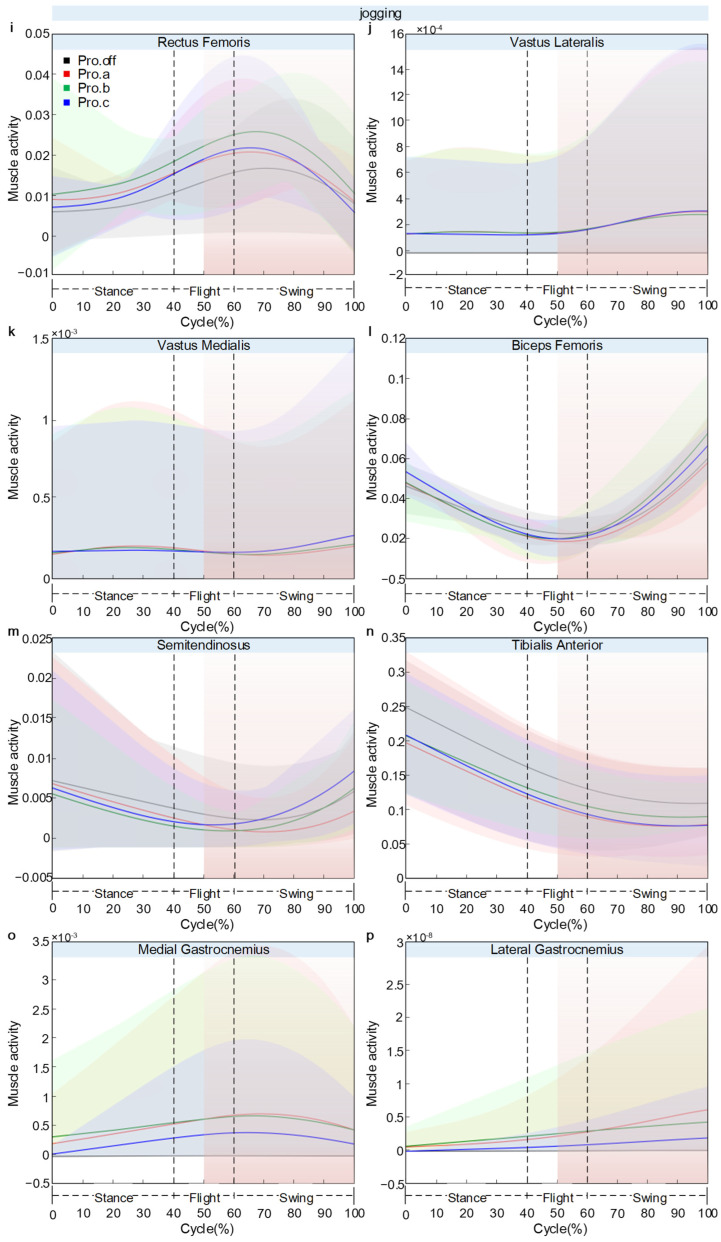

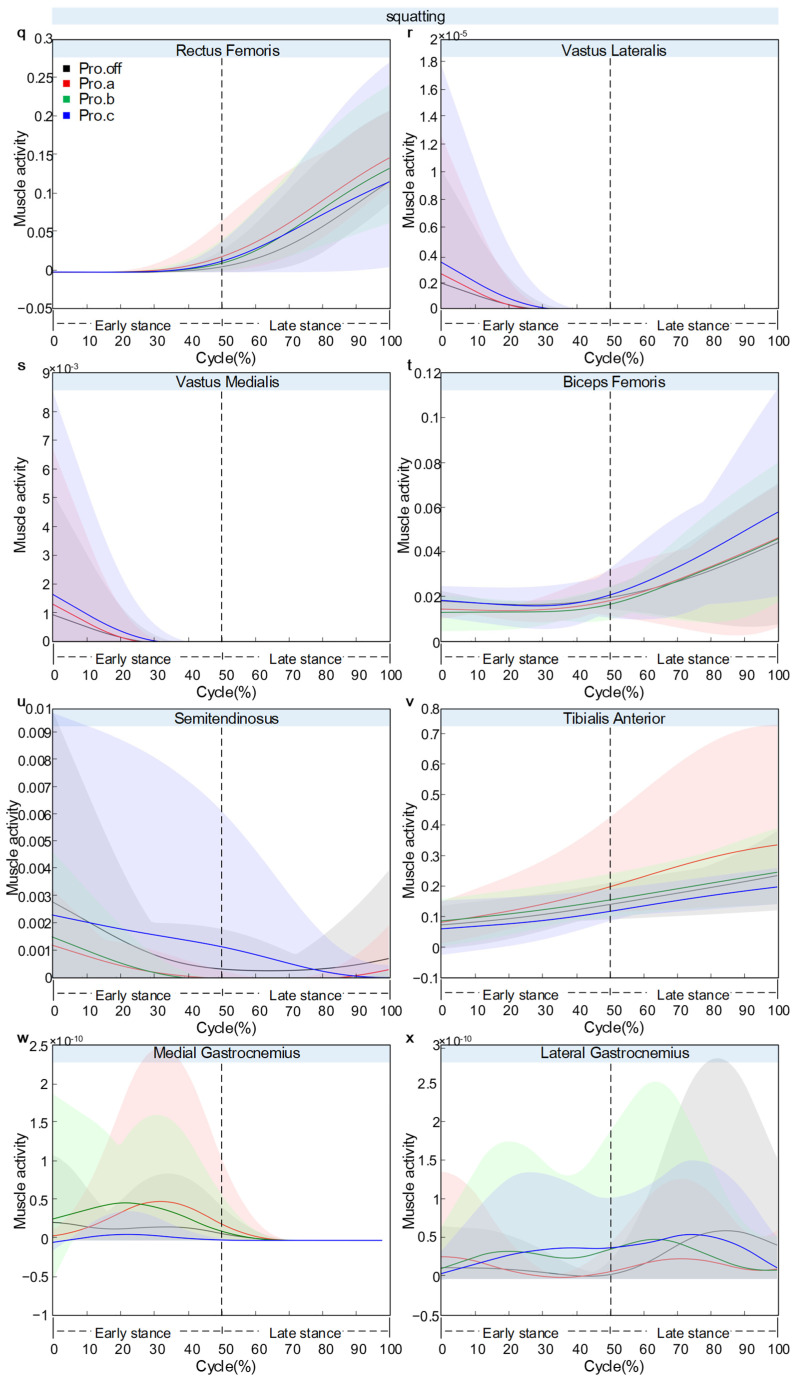

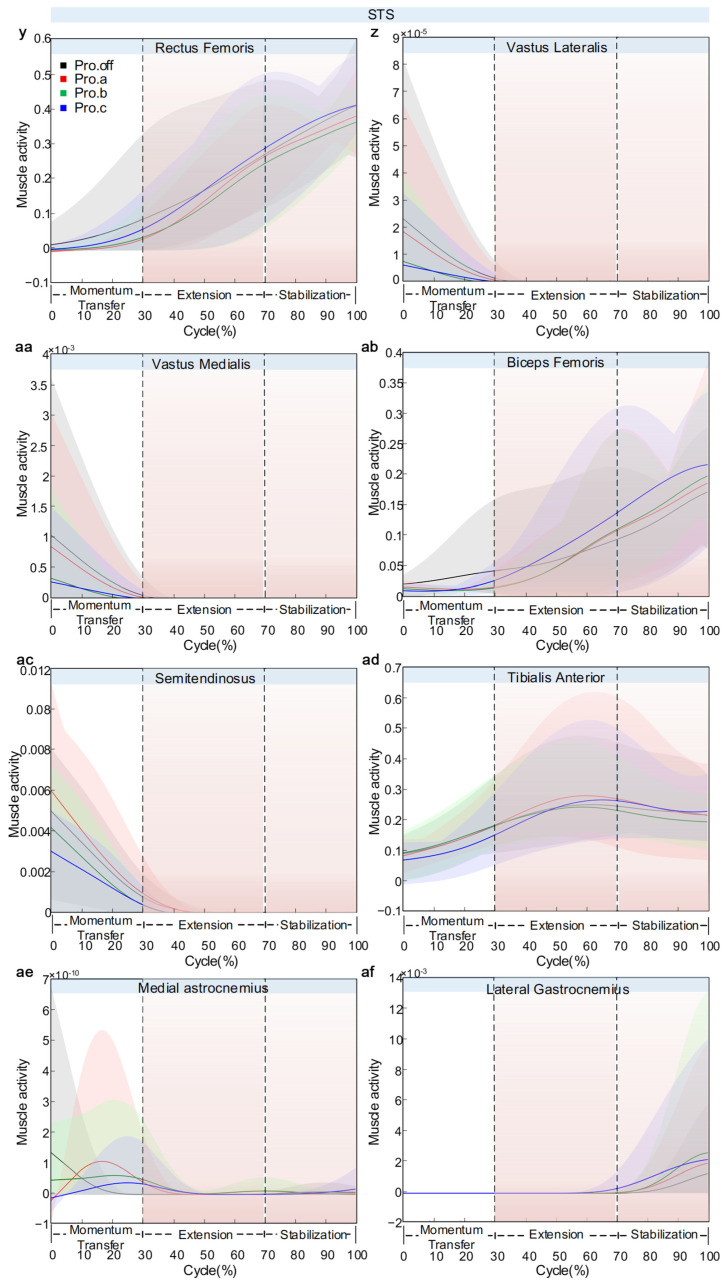
Knee joint muscle activation diagram over the normalized cycle: Panels (**a**–**h**) show walking, panels (**i**–**p**) show jogging, panels (**q**–**x**) show squatting, and panels (**y**–**af**) show STS. Within each task, the subfigures respectively correspond to rectus femoris, vastus lateralis, vastus medialis, biceps femoris, semitendinosus, tibialis anterior, medial gastrocnemius, and lateral gastrocnemius. Solid line: mean, shaded area: ±SD across participants (*n* = 5). Muscle activity denotes the AnyBody Activity output (dimensionless fraction of MVC, 0–1), not EMG.

Figure 12 presents the variations in model-estimated muscle activity for eight lower-limb muscles: rectus femoris (a), vastus lateralis (b), vastus medialis (c), biceps femoris (d), semitendinosus (e), tibialis anterior (f), medial gastrocnemius (g), and lateral gastrocnemius (h). Across tasks, between-condition separations were phase-localized rather than uniform throughout the full cycle, enabling task- and phase-specific comparisons of protector effects on muscle activity.

During walking, the muscles exhibited a temporally coordinated activation pattern. In the early stance phase (0–20% of the gait cycle), the tibialis anterior reached its first activation peak (0.13), accompanied by the activation of the gastrocnemius group and the first peak of the rectus femoris (0.05). A key transition occurred around 60–70% of the way through the cycle. At this stage, several muscles showed synchronized activation peaks: vastus lateralis (0.006), vastus medialis (0.025), biceps femoris (0.069), semitendinosus (0.334), and the second peak of the tibialis anterior (0.248). Notable between-condition differences in the rectus femoris were observed at the end of the gait cycle (80–100%), where Pro.c showed a sharp increase up to 0.014. Beyond this end-phase divergence, Pro.c elevated the major peak amplitudes across several muscles in a consistent direction (rectus femoris +55%, vastus lateralis +100%, biceps femoris +40%, tibialis anterior +47%), whereas Pro.a reduced most activations by 15–25%. In addition, Pro.c introduced a distinct medial gastrocnemius peak near 60% that was absent under the other configurations, marking a configuration-specific timing feature rather than a simple amplitude scaling. SD band widened near peak regions, indicating that inter-individual variability was concentrated at high-activation phases.

During jogging, the activation profiles were more differentiated across muscle groups, and the main between-condition separations were concentrated in late-cycle propulsion (>50%) rather than distributed throughout the stride. Within the quadriceps group, the rectus femoris reached its peak activation during the propulsion phase (60–70% of the cycle), with Pro.b showing the highest value (0.026) and Pro.a the lowest (0.017). The vastus lateralis demonstrated a continuous increase, with all four curves closely overlapping. At the same time, the vastus medialis also showed an upward trend, with slight divergence near the end, but with no clear between-condition differences. The hamstring group showed greater variation. The biceps femoris showed a U-shaped activation curve, with Pro.b reaching 0.088 at the end of the cycle—an increase of about 32.95% compared to Pro.a (0.059). The semitendinosus exhibited minimal change across conditions. The distal muscles presented evident compensatory behavior. The tibialis anterior showed a progressive decline in activation, with Pro.c (0.208) being 16.12% lower than Pro.off (0.248). The medial gastrocnemius demonstrated a linear increase, with Pro.a reaching the highest terminal value. In contrast, the lateral gastrocnemius showed a peaked pattern, with the highest activation observed under Pro.b. The key inter-condition differences were concentrated in the propulsion phase beyond 50% of the gait cycle.

During the squatting movement, the rectus femoris and tibialis anterior exhibited continuously increasing activation patterns. The rectus femoris rose sharply between 60% and 100% of the cycle, with Pro.a showing the highest activation (0.142), followed by the segmented-woven configuration (0.132). The tibialis anterior displayed a linear increase, reaching a peak of 0.348 under Pro.a, while Pro.c showed the lowest activation (0.187). Second, vastus lateralis, vastus medialis, and semitendinosus exhibited early peaks followed by rapid declines, with Pro.c showing higher early-peak amplitudes relative to other conditions. Biceps femoris reached a minimum around 30–40%, but under Pro.c it increased notably toward the end of the motion, creating a late-cycle divergence localized to the hamstring flexor. The vastus lateralis, vastus medialis, and semitendinosus all exhibited early activation peaks followed by a rapid decline, with Pro.c showing relatively higher peak values. The gastrocnemius muscles showed very low overall activation levels. In aggregate, protectors did not produce a uniform scaling pattern across all muscles. Instead, Pro.a preferentially elevated rectus femoris and tibialis anterior activity, whereas Pro.c preferentially elevated biceps femoris and early-phase hamstring-group activity.

During STS, rectus femoris increased continuously and showed a clear terminal ranking across conditions, with Pro.c reaching the highest end value (0.434) and Pro.b the lowest (0.364), indicating configuration-dependent separation in late-cycle proximal activation. Vastus lateralis, vastus medialis, and semitendinosus displayed early peaks followed by a rapid decline. Pro.off showed higher initial peaks than protector-wearing conditions at the semitendinosus. Biceps femoris followed a bell-shaped activation pattern, with a peak at 90–100%, where Pro.c reached slightly higher values than the other conditions, while tibialis anterior also showed a bell-shaped pattern peaking at 50–70% with the highest activation under Pro.a. Medial gastrocnemius rose during 10–30% and showed the highest activation under Pro.b and the lowest under Pro.off, identifying a strong late-phase configuration effect in distal plantarflexor activity. Lateral gastrocnemius remained near zero overall, with a small late peak (70–100%). Overall, STS data revealed a phase-dependent redistribution: early-cycle peaks in several muscles were highest without a protector, whereas late-cycle amplification is configuration-specific (rectus femoris: Pro.c highest, medial gastrocnemius: Pro.b highest).

## 4. Discussion

### 4.1. Key Findings

Knee joint protectors are widely used to modify knee mechanics during physical activity. In this pilot study (*n* = 5), we compared three protector designs (Pro.a–Pro.c) with an unprotected condition (Pro.off) across walking, jogging, squatting, and sit-to-stand tasks using motion capture and AnyBody musculoskeletal modeling. The results describe task- and phase-dependent changes in knee kinematics, model-estimated joint reaction forces, moments, and model-estimated muscle activity. Across tasks, protector effects were plane-specific: during more dynamic movements, changes were mainly expressed in sagittal-plane knee flexion, whereas under quasi-static or low-dynamic conditions, changes were more evident in coronal-plane loading components. Within this dataset, increasing structural constraint did not consistently reduce loading proxies. In some tasks, higher constraints coincided with increased joint reaction force components or muscle activity. These findings support task-specific evaluation of protector designs and are interpreted as descriptive patterns rather than population-level causal effects.

### 4.2. Model Fitting and Parameter Estimation

The reliability of parameter fitting using the Anybody marker set was evaluated. After proportionally scaling and matching the overall musculoskeletal model, the fitting process for the walking condition was completed rapidly, at approximately 10% of the motion cycle. In contrast, the other three motion states required fitting to complete around 35% of the cycle. Although the fitting speed varied across movement types, the overall fitting error remained low, indicating high fitting accuracy. The maximum fitting errors were 0.5, 1.1, 0.26, and 0.49, respectively. These between-task differences are consistent with increased soft tissue deformation and protector–limb interaction at higher movement intensities. The Pro.b condition showed greater parameter deviations across most motion patterns, consistent with rigid side supports altering knee kinematic constraints and affecting marker-based alignment. In contrast, Pro.c (Pro.b with additional strapping) exhibited parameter curves closer to Pro.off, indicating a more similar fitted-parameter profile to the unprotected condition. Pro.a demonstrated high similarity to Pro.off across all motion types, indicating that uniformly distributed elastic compression has only a minimal influence on kinematic parameters.

### 4.3. Knee Flexion Modulation Across Tasks

Regarding knee flexion angle, the knee joint protector exerted an influence during walking, particularly in the early swing phase. The Pro.off condition exhibited the largest flexion angle, reflecting a natural gait pattern. Pro.c reduced the first flexion peak by 27%, indicating that a strong mechanical constraint limited knee swing amplitude. The smaller difference observed at the second peak suggests that kinematic behavior converged during the late stance phase. The protector altered the range of motion mainly during the swing phase but had a limited effect during stance, a selective influence that may impact gait efficiency and energy expenditure [23]. During running, the protectors showed a progressively restrictive effect on knee flexion. Pro.off again presented the greatest flexion amplitude, corresponding to natural movement. The degree of mechanical constraint was proportional to the reduction in flexion: Pro.a and Pro.b induced moderate restriction, while Pro.c produced the greatest limitation. The 7% difference in flexion amplitude suggests that excessive constraint during high-speed motion can hinder natural knee flexion, potentially reducing gait efficiency. Hence, the protector design should account for movement velocity to avoid over-restriction. In squatting, the protectors limited the maximum flexion angle, with restriction intensity proportional to constraint strength. Pro.c produced the greatest limitation, indicating that a strong mechanical constraint physically prevented deep flexion [24]. This restriction likely occurred through two mechanisms: (1) direct mechanical blocking and (2) increased flexion resistance leading to voluntary motion termination. The Pro.off condition achieved the greatest flexion, reflecting the natural range of joint motion. Protector design should therefore balance protection and functional mobility, as excessive limitation may impair daily activities such as deep squatting. The kinematic features observed during the STS movement revealed a paradoxical effect of protector design. Although Pro.b theoretically imposed a greater constraint than Pro.off, it paradoxically increased the flexion amplitude—possibly because its rigid supports provided enhanced stability, improving user confidence and allowing deeper initial flexion. Meanwhile, Pro.c, despite being the most restrictive by design, did not impose a clear limitation, suggesting that excessive constraint triggered protective motor strategies, in which users voluntarily reduced flexion to avoid discomfort. This psychobiomechanical interaction implies that protector effectiveness depends not only on its mechanical properties but also on user perception and adaptive motor control strategies [25].

### 4.4. Joint Reaction Forces

Regarding joint reaction forces, during walking, Pro.b reduced the peak load in the X direction. This may be attributed to its rigid support bars, which effectively limit varus–valgus motion of the knee, thereby altering the load transmission path and transferring part of the medial–lateral stress to the protector structure. Conversely, Pro.a increased the medial–lateral load at the first peak, suggesting that uniform elastic compression may modify the early stance gait pattern. The high consistency of reaction forces in the Y and Z directions indicates that protector design primarily affects frontal-plane stability of the knee, while exerting limited influence on sagittal-plane motion—consistent with the fact that walking mainly occurs in the sagittal plane. Notably, Pro.c did not alter joint load distribution as expected. Its curve characteristics fell between those of Pro.off and Pro.b, suggesting that excessive constraint may induce compensatory motion and shift the load distribution toward the unbraced pattern. The small difference in Z-direction load (approximately 40 N, about 7% of the peak) suggests that protectors have minimal effect on weight-bearing capacity. However, in clinical applications, such cumulative effects may still exert long-term influence on articular cartilage [26]. During running, structurally reinforced protectors exhibited loading patterns opposite to expectations. In the X direction, Pro.b and Pro.c not only failed to reduce load but instead increased peak reaction forces by 15.6%. This may result from the rigid support bars restricting the knee’s natural shock-absorbing motion, thereby increasing lateral stress on the articular surface. This finding indicates that excessive mechanical constraint during high-speed movement may produce adverse effects, contradicting the original intent of protector design. The consistency in Y-direction loads suggests that propulsion in running is primarily governed by precise neuromuscular control, which external protectors can scarcely influence within such a highly coordinated motor pattern [27]. The minor difference in the Z-direction load (2.6%) confirms that weight-bearing transmission is primarily skeletal, with negligible involvement of the protective system. Interestingly, Pro.b exhibited the lowest X-direction load when the protector was worn, suggesting that moderate and evenly distributed compression may be more suitable than rigid constraint for high-dynamic activities. These findings challenge the traditional notion that stronger structural support automatically yields better protection. In fact, during high-speed motion such as running, maintaining moderate freedom of knee movement may better facilitate natural shock absorption and load distribution mechanisms. In squatting, the load distribution presented features opposite to expectations. Pro.c produced the highest or near-highest load in all directions, likely because its uniform pressure design became detrimental during large flexion angles—restricting normal muscle contraction and fine joint adjustment, thereby forcing the joint to bear greater direct load. In contrast, Pro.b exhibited relatively lower loads, suggesting that its combination of differential knitting and rigid side supports provided a more rational load distribution during quasi-static motion, allowing adaptive joint adjustment within a constrained range. Notably, the largest difference was observed in the Z-direction load (10.5%), indicating that protector effects on weight-bearing are more pronounced in quasi-static movements, in sharp contrast to findings under dynamic conditions. These results suggest that for protectors designed for deep-flexion, quasi-static movements such as squatting, excessive uniform constraint should be avoided. Instead, a zonal and differentiated support strategy should be employed—providing necessary stability while preserving the joint’s adaptive regulatory capacity [28]. In the STS (sit-to-stand) movement, protectors mainly affected medial–lateral load distribution (a 10.3% difference), while exerting minimal influence on Y- and Z-direction loads. Pro.c increased X-direction load, likely due to its reinforced constraints limiting natural lateral adjustments. In contrast, Pro.a reduced X-direction load, indicating that moderate elastic support aids in load dispersion. The high consistency in Y- and Z-direction loads demonstrates that the primary driving forces in STS are controlled by large muscle groups, with limited mechanical contribution from the protector. Unlike dynamic movements, the quasi-static nature of STS causes protector effects to manifest primarily in the auxiliary (frontal) plane rather than the main (sagittal) plane [29].

### 4.5. Joint Torques

Regarding joint torque, the clear differentiation in flexion torque during the terminal phase of walking reveals the protector’s phase-specific effects within the gait cycle. The high consistency observed during the first 80% of the cycle suggests that under normal gait conditions, protectors have minimal influence on torque patterns, as neuromuscular control mechanisms dominate movement. The sharp divergence near the end phase likely corresponds to the swing-preparation stage, where Pro.c generates the greatest resistance—requiring approximately 50% more flexion torque to overcome the constraint. These data indicate a phase-localized increase in model-estimated flexion moment during terminal walking under higher-constraint conditions. In the present pilot dataset, this pattern suggests that excessive terminal-phase restriction may increase the mechanical demand required for swing preparation. During running, protectors produced differentiated effects on flexion torque. The Pro.off torque trough remained positive, indicating that under natural conditions, muscles maintain a baseline tension to stabilize the joint. Pro.a generated the largest negative torque but had the smallest terminal torque, suggesting that enhanced eccentric braking might reduce subsequent propulsion demands. Both Pro.b and Pro.c exhibited nearly identical, highest terminal torque values, implying that structural support increases resistance during the propulsion phase. These endpoint differences highlight the necessity for protector designs to balance braking capacity and propulsion efficiency. During squatting, protectors had almost no influence on lower-limb flexion torque, demonstrating that the biomechanical effects of protectors are highly joint-specific. Although knee movement restriction theoretically increases compensatory loading at the hip joint, in this study, the constraint on knee flexion was minimal and insufficient to trigger a kinetic chain compensation. According to squat biomechanics, the trunk–tibia angle is a key determinant of hip–knee torque distribution. When this angle remains stable, hip joint torque does not change appreciably [30]. Despite having the strongest constraint, Pro.c showed the least torque variability, possibly due to enhanced mechanical stability and improved proprioceptive feedback, which allowed participants to execute movements with higher precision, thereby reducing inter-individual variability [31]. In the STS (sit-to-stand) task, protectors exhibited a pronounced torque-modulating effect. Pro.b produced the greatest reduction in torque, likely because its rigid supports effectively shared the load during the standing-maintenance phase. Although Pro.c provided greater constraint, its torque was only slightly lower than that of Pro.off, suggesting that excessive restriction may impair load-bearing efficiency. Its effect was intermediate between the two. The overlap observed in the early phase indicates that the explosive rising stage relies mainly on muscular drive, while the protector primarily contributes support during the static stabilization period.

### 4.6. Muscle Activation

In terms of muscle activation, during walking, the changes induced by knee joint protectors reflected complex neuromuscular compensation mechanisms. The excessive constraint of Pro.c led to enhanced co-contraction of the antagonist muscles [32]. The lower model-estimated activity observed with Pro.a may reflect reduced demand for active stabilization under external compression [33]. The abnormal activation of the gastrocnemius may reflect distal compensation. One possible explanation is that the protector changed muscle activation patterns and external force transmission, which reduced medial knee loading [34]. Taken together, these model-estimated activation patterns suggest that protector effects on neuromuscular demand are task-specific and not uniform across muscle groups [35]. During running, protector-induced changes in muscle activation reflected a complex neuromuscular adaptation process. Pro.b increased the activation of the rectus femoris and biceps femoris, indicating that rigid support altered the coordination between the knee extensors and flexors [36]. The decreased activation of the tibialis anterior suggests adjustments in ankle strategy. The differentiated responses between the medial and lateral heads of the gastrocnemius reveal that different protector types exert selective influence on ankle joint control [37]. In squatting, the protector altered muscle recruitment strategies by providing external support. Pro.a increased quadriceps activation, possibly because its uniform compression enhanced proprioceptive feedback and promoted synergistic contraction of the knee extensors [38]. Pro.c increased terminal activation of the biceps femoris, indicating that its unique strap-based structure heightened the stability demands of the knee flexors. Pro.b showed intermediate activation levels, suggesting that its rigid side partially compensated for muscular stabilization. The extremely low activation of the gastrocnemius indicates that squatting primarily relies on proximal muscle control. These findings demonstrate that different protector designs induce specific neuromuscular adaptation patterns by altering the joint’s mechanical environment, and that their mechanical characteristics directly influence movement control strategies. During the STS movement, the continuous increase in rectus femoris activation corresponded to the progressively increasing demand for knee extension. The enhanced activation in Pro.c likely resulted from additional constraints increasing muscle recruitment requirements. The early peaks observed in the vastus lateralis, vastus medialis, and semitendinosus corresponded to stability demands during movement initiation. Their reduced activation with bracing indicates that external support partially substituted for intrinsic muscular stabilization [39]. The bell-shaped activation of the biceps femoris and tibialis anterior reflected control needs during mid-phase posture transition, with Pro.a showing enhanced activation likely due to its uniform compression, which altered the joint’s mechanical environment [40]. The late-phase increase in medial gastrocnemius activation corresponded to the need for standing stabilization, and the high activation observed with Pro.b suggests that its rigid supports modified the ankle joint strategy [41]. This differentiated pattern of muscle activation indicates that protectors induce specific neuromuscular control strategies by altering joint constraint conditions. Therefore, in clinical applications, protector selection should be tailored to the patient’s functional demands and movement characteristics.

### 4.7. Limitations and Future Directions

This study still has certain limitations. First, inter-individual variability may have been amplified under different movement conditions, leading to potential instability in the observed characteristic changes. The 5 min familiarization per condition may be insufficient to ensure steady-state strategies and may allow transient adaptation effects to persist. The study population should also be expanded beyond healthy young participants in future work. In addition, models based solely on kinematic data may exhibit reduced accuracy in estimating joint forces [42]. Though GRF prediction has been evaluated in prior work, prediction errors may propagate to model-estimated joint reaction forces and moments, with higher sensitivity expected near peak loading and during rapid foot–ground contact transitions. Given the pilot cohort size, we report group-level patterns as mean ± SD and do not perform confirmatory hypothesis testing in the current manuscript. Such inference will be conducted in a larger follow-up study with a priori power analysis based on the effect magnitudes observed here. No sEMG was collected because the protector overlap may compromise electrode placement and signal integrity. Future work will use access windows or low-profile EMG systems. Future research will incorporate measured GRF (instrumented treadmill, force plates or wearable in-shoe systems) and a dedicated sensitivity analysis to quantify how GRF perturbations affect knee joint reaction forces across phases.

## 5. Conclusions

Based on motion capture and AnyBody musculoskeletal simulation, this pilot study characterized task- and phase-dependent biomechanical differences among knee joint protectors. In several tasks, rigid-support designs (Pro.b/Pro.c) showed larger deviations in model-estimated joint loading components and muscle activity. In contrast, elastic compression (Pro.a) showed smaller deviations from Pro.off in some metrics. Overall, greater structural constraint did not consistently produce a more favorable biomechanical profile. These descriptive findings provide a feasibility baseline for task-specific protector evaluation and support future confirmatory studies with larger cohorts, measured GRF, and sEMG validation.

## Data Availability

The authors confim that the data supporting the findings of this study are available within the article and its Supplementary Materials.

## Supplementary Materials

The following supporting information can be downloaded at: https://www.mdpi.com/article/10.3390/s26072168/s1, Table S1: Peak values under various tasks and conditions; Table S2: Full normalized knee joint reaction force data during walking under four knee protector conditions (group mean ± SD, *n* = 5); Table S3: Full normalized knee joint reaction force data during jogging under four knee protector conditions (group mean ± SD, *n* = 5); Table S4: Full normalized knee joint reaction force data during squatting under four knee protector conditions (group mean ± SD, *n* = 5); Table S5: Full normalized knee joint reaction force data during STS under four knee protector conditions (group mean ± SD, *n* = 5); Table S6: Full normalized knee flexion moment data during walking under four knee protector conditions (group mean ± SD, *n* = 5); Table S7: Full normalized knee flexion moment data during jogging under four knee protector conditions (group mean ± SD, *n* = 5); Table S8: Full normalized knee flexion moment data during squatting under four knee protector conditions (group mean ± SD, *n* = 5); Table S9: Full normalized knee flexion moment data during STS under four knee protector conditions (group mean ± SD, *n* = 5).
