# Supplementary material for: Study on the Influence of Protector Design on the Biomechanical Characteristics of Knee Joint Movement
Source: Sensors (Basel). 2026 Mar 31;26(7):2168. doi: 10.3390/s26072168 (PMC13075084; doi:10.3390/s26072168)
Supplement: Supplementary file 1 [file sensors-26-02168-s001.zip › sensors-4156715-supplementary.pdf]

Supplementary Materials

Study on the influence of protector design on the biomechanical characteristics of knee joint movement

Jiaxin Zhao [1,2], Xupeng Wang [1,2]*, Lingxiao Xi [1], Xinran Cheng [1], Jihyun Bae[3]* and Yongwei Li[3]

1 School of Mechanical Engineering, Xi'an University of Technology, Xi'an 710048, China

2 School of Art and Design, Xi'an University of Technology, Xi'an 710054, China

3 Human-Tech Convergence Program Department of Clothing & Textiles Hanyang University Seoul 04763, Republic of Korea

* Corresponding author: wangxupeng@xaut.edu.cn (X. Wang), jbae2@hanyang.ac.kr (J. Bae)

This supplement contains:

Table S1 Peak values under various tasks and conditions.

Table S2 Full normalized knee joint reaction force data during walking under four knee protector conditions (group mean ± SD, n = 5).

Table S3 Full normalized knee joint reaction force data during jogging under four knee protector conditions (group mean ± SD, n = 5).

Table S4 Full normalized knee joint reaction force data during squatting under four knee protector conditions (group mean ± SD, n = 5).

Table S5 Full normalized knee joint reaction force data during STS under four knee protector conditions (group mean ± SD, n = 5).

Table S6 Full normalized knee flexion moment data during walking under four knee protector conditions (group mean ± SD, n = 5).

Table S7 Full normalized knee flexion moment data during jogging under four knee protector conditions (group mean ± SD, n = 5).

Table S8 Full normalized knee flexion moment data during squatting under four knee protector conditions (group mean ± SD, n = 5).

Table S9 Full normalized knee flexion moment data during STS under four knee protector conditions (group mean ± SD, n = 5).

Tab. S1 Peak values under various tasks and conditions

Angle(rad) (walk)	Angle(rad) (jogging)	Angle(rad) (squatting)	Angle(rad) (STS)
1.04	0.86	1.15	1.68
0.95	0.85	1.14	1.7
0.91	0.83	1.14	1.65
0.85	0.82	1.13	1.62
Knee MedioLateral(N) (walk)	Knee MedioLateral(N) (jogging)	Knee MedioLateral(N) (squatting)	Knee MedioLateral(N) (STS)
39.02	32.14	58.64	263.84
35.58	35.05	64.51	254.78
33.3	35.12	65.81	261.01
31.31	35.55	71.14	279.39
Knee AnteroPosterior Force (N)(walk)	Knee AnteroPosterior Force (N)(jogging)	Knee AnteroPosterior Force (N)(squatting)	Knee AnteroPosterior Force (N)(STS)
-28.18	-128.25	-20.017	-106.56
-18.12	-126.79	-21.34	-20.03
-32.18	-140.26	-25.4	-27.83
-20.42	-136.18	-21.28	-33.45
Knee ProximoDistal Force (N)(walk)	Knee ProximoDistal Force (N)(jogging)	Knee ProximoDistal Force (N)(squatting)	Knee ProximoDistal Force (N)(STS)
-180.95	-259.26	-168.06	-202.68
-189.61	-251.46	-148.61	-168.92
-189.78	-231.53	-153.36	-168.7
-187.8	-258.76	-161.13	-160.68
Torque (N·m) (walk)	Torque (N·m) (jogging)	Torque (N·m) (squatting)	Torque (N·m) (STS)
4.02	7.08	3.35	2.85
4.76	4.56	3.17	2.66
3.58	7.35	3.19	2.59
4.35	6.31	3.29	2.65

Muscle activity (Rectus Femoris) (walk)	Muscle activity (Vastus Lateralis) (walk)	Muscle activity (Vastus Medialis) (walk)	Muscle activity (Biceps Femoris) (walk)	Muscle activity (Semitendinosus) (walk)	Muscle activity (Tibialis Anterior) (walk)	Muscle activity (Medial Gastrocnemius) (walk)	Muscle activity (Lateral Gastrocnemius) (walk)
0.045	0.004	0.018	0.069	0.177	0.2	1.94E-10	3.17E-10
0.051	0.005	0.022	0.057	0.271	0.207	1.98E-10	1.49E-10
0.042	0.004	0.017	0.061	0.227	0.204	2.12E-10	9.13E-11
0.047	0.006	0.025	0.068	0.334	0.219	2.86E-10	1.05E-10
Muscle activity	Muscle activity	Muscle activity	Muscle activity	Muscle activity	Muscle activity	Muscle activity	Muscle activity

(Rectus Femoris) (jogging)	(Vastus Lateralis) (jogging)	(Vastus Medialis) (jogging)	(Biceps Femoris) (jogging)	(Semitendinosus) (run)	(Tibialis Anterior) (jogging)	(Medial Gastrocnemius) (jogging)	(Lateral Gastrocnemius) (jogging)
0.017	5.26E-09	8.17E-12	0.067	0.063	0.248	8.17E-06	6.23E-11
0.021	3.00E-04	2.00E-04	0.059	0.059	0.197	5.94E-04	6.96E-09
0.026	2.80E-04	2.60E-04	0.088	0.058	0.207	4.41E-04	4.64E-09
0.022	3.10E-04	3.10E-04	0.059	0.078	0.208	2.33E-04	2.88E-09
Muscle activity (Rectus Femoris) (squatting)	Muscle activity (Vastus Lateralis) (squatting)	Muscle activity (Vastus Medialis) (squatting)	Muscle activity (Biceps Femoris) (squatting)	Muscle activity (Semitendinosus) (squatting)	Muscle activity (Tibialis Anterior) (squatting)	Muscle activity (Medial Gastrocnemius) (squatting)	Muscle activity (Lateral Gastrocnemius) (squatting)
0.127	2.14E-06	9.17E-04	0.041	0.003	0.227	2.86E-11	5.12E-11
0.142	2.85E-06	1.37E-03	0.046	0.001	0.348	4.62E-11	2.32E-11
0.132	1.02E-08	1.31E-09	0.058	0.002	0.248	4.51E-11	4.98E-11
0.115	3.72E-06	1.70E-03	0.044	0.002	0.187	5.80E-12	5.05E-11
Muscle activity (Rectus Femoris) (STS)	Muscle activity (Vastus Lateralis) (STS)	Muscle activity (Vastus Medialis) (STS)	Muscle activity (Biceps Femoris) (STS)	Muscle activity (Semitendinosus) (STS)	Muscle activity (Tibialis Anterior) (STS)	Muscle activity (Medial Gastrocnemius) (STS)	Muscle activity (Lateral Gastrocnemius) (STS)
0.41	2.38E-05	1.00E-04	0.017	0.005	0.259	1.20E-10	1.08E-03
0.383	1.91E-05	8.00E-05	0.196	0.006	0.288	9.70E-11	1.81E-03
0.364	8.22E-06	3.70E-05	0.207	0.004	0.251	5.10E-11	2.27E-03
0.434	6.90E-06	3.10E-05	0.219	0.003	0.275	1.20E-11	2.10E-03

Tab. S2 Full normalized knee joint reaction force data during walking under four knee protector conditions (group mean ± SD, n = 5).

Percent cycle (%)	Fx/BW Pro.off	Fx/BW Pro.a	Fx/BW Pro.b	Fx/BW Pro.c	Fy/BW Pro.off	Fy/BW Pro.a	Fy/BW Pro.b	Fy/BW Pro.c	Fz/BW Pro.off	Fz/BW Pro.a	Fz/BW Pro.b	Fz/BW Pro.c
0	0.003±0.003	0.004 ± 0.004	0.005 ± 0.003	0.003 ± 0.003	-0.055 ± 0.025	-0.055 ± 0.042	-0.070 ± 0.049	-0.053 ± 0.023	-0.300 ± 0.052	-0.314 ± 0.067	-0.294 ± 0.039	-0.305 ± 0.059
1	0.003 ± 0.003	0.003 ± 0.005	0.007 ± 0.004	0.003 ± 0.003	-0.059 ± 0.027	-0.059 ± 0.043	-0.077 ± 0.057	-0.056 ± 0.024	-0.295 ± 0.046	-0.304 ± 0.070	-0.298 ± 0.041	-0.310 ± 0.059
2	0.004 ± 0.004	0.002 ± 0.006	0.009 ± 0.006	0.004 ± 0.004	-0.064 ± 0.031	-0.063 ± 0.044	-0.084 ± 0.067	-0.059 ± 0.024	-0.290 ± 0.043	-0.298 ± 0.071	-0.300 ± 0.044	-0.314 ± 0.058
3	0.005 ± 0.005	0.003 ± 0.006	0.011 ± 0.009	0.005 ± 0.004	-0.071 ± 0.035	-0.068 ± 0.046	-0.092 ± 0.078	-0.064 ± 0.025	-0.287 ± 0.042	-0.294 ± 0.070	-0.301 ± 0.049	-0.316 ± 0.055

4	0.006 ± 0.007	0.003 ± 0.007	0.012 ± 0.012	0.006 ± 0.005	-0.078 ± 0.041	-0.074 ± 0.049	-0.099 ± 0.087	-0.070 ± 0.026	-0.284 ± 0.043	-0.293 ± 0.068	-0.301 ± 0.056	-0.317 ± 0.049
5	0.007 ± 0.008	0.005 ± 0.008	0.014 ± 0.015	0.009 ± 0.006	-0.086 ± 0.047	-0.081 ± 0.051	-0.106 ± 0.095	-0.078 ± 0.026	-0.283 ± 0.043	-0.293 ± 0.065	-0.302 ± 0.063	-0.317 ± 0.041
6	0.009 ± 0.010	0.007 ± 0.009	0.016 ± 0.017	0.012 ± 0.008	-0.096 ± 0.052	-0.090 ± 0.054	-0.112 ± 0.099	-0.088 ± 0.026	-0.284 ± 0.042	-0.295 ± 0.061	-0.302 ± 0.069	-0.316 ± 0.035
7	0.012 ± 0.011	0.010 ± 0.011	0.017 ± 0.018	0.016 ± 0.010	-0.106 ± 0.057	-0.101 ± 0.057	-0.117 ± 0.100	-0.102 ± 0.027	-0.287 ± 0.041	-0.298 ± 0.057	-0.303 ± 0.073	-0.316 ± 0.032
8	0.016 ± 0.012	0.014 ± 0.013	0.019 ± 0.019	0.021 ± 0.013	-0.118 ± 0.060	-0.115 ± 0.060	-0.122 ± 0.097	-0.118 ± 0.028	-0.293 ± 0.040	-0.300 ± 0.051	-0.304 ± 0.075	-0.316 ± 0.033
9	0.020 ± 0.012	0.018 ± 0.015	0.021 ± 0.018	0.026 ± 0.016	-0.131 ± 0.062	-0.131 ± 0.061	-0.127 ± 0.092	-0.137 ± 0.032	-0.301 ± 0.038	-0.303 ± 0.044	-0.307 ± 0.074	-0.319 ± 0.034
10	0.024 ± 0.011	0.023 ± 0.017	0.023 ± 0.018	0.031 ± 0.018	-0.146 ± 0.060	-0.150 ± 0.060	-0.132 ± 0.086	-0.158 ± 0.038	-0.310 ± 0.037	-0.306 ± 0.036	-0.311 ± 0.071	-0.323 ± 0.037
11	0.029 ± 0.011	0.028 ± 0.018	0.025 ± 0.018	0.036 ± 0.020	-0.161 ± 0.056	-0.172 ± 0.057	-0.137 ± 0.081	-0.180 ± 0.046	-0.319 ± 0.036	-0.309 ± 0.026	-0.315 ± 0.068	-0.330 ± 0.040
12	0.034 ± 0.010	0.033 ± 0.017	0.027 ± 0.019	0.041 ± 0.021	-0.178 ± 0.050	-0.195 ± 0.051	-0.143 ± 0.077	-0.200 ± 0.054	-0.329 ± 0.037	-0.314 ± 0.018	-0.321 ± 0.064	-0.338 ± 0.045
13	0.039 ± 0.009	0.039 ± 0.016	0.029 ± 0.020	0.044 ± 0.021	-0.194 ± 0.040	-0.217 ± 0.043	-0.149 ± 0.076	-0.219 ± 0.062	-0.339 ± 0.040	-0.321 ± 0.016	-0.329 ± 0.061	-0.348 ± 0.054
14	0.042 ± 0.008	0.044 ± 0.015	0.031 ± 0.021	0.047 ± 0.021	-0.209 ± 0.031	-0.236 ± 0.034	-0.156 ± 0.075	-0.233 ± 0.067	-0.348 ± 0.045	-0.331 ± 0.023	-0.337 ± 0.058	-0.358 ± 0.065
15	0.046 ± 0.009	0.049 ± 0.013	0.033 ± 0.022	0.049 ± 0.021	-0.222 ± 0.026	-0.251 ± 0.027	-0.162 ± 0.075	-0.241 ± 0.068	-0.357 ± 0.051	-0.343 ± 0.032	-0.346 ± 0.056	-0.369 ± 0.077
16	0.048 ± 0.010	0.052 ± 0.012	0.035 ± 0.023	0.050 ± 0.021	-0.229 ± 0.032	-0.258 ± 0.026	-0.168 ± 0.073	-0.243 ± 0.066	-0.366 ± 0.058	-0.356 ± 0.040	-0.355 ± 0.054	-0.379 ± 0.088
17	0.049 ± 0.013	0.053 ± 0.012	0.036 ± 0.023	0.050 ± 0.020	-0.231 ± 0.043	-0.258 ± 0.030	-0.174 ± 0.070	-0.239 ± 0.060	-0.374 ± 0.065	-0.371 ± 0.047	-0.365 ± 0.051	-0.387 ± 0.098
18	0.049 ± 0.016	0.054 ± 0.013	0.038 ± 0.024	0.049 ± 0.019	-0.227 ± 0.055	-0.250 ± 0.035	-0.178 ± 0.067	-0.228 ± 0.051	-0.383 ± 0.072	-0.387 ± 0.053	-0.376 ± 0.049	-0.394 ± 0.106
19	0.047 ± 0.018	0.052 ± 0.015	0.039 ± 0.024	0.047 ± 0.019	-0.216 ± 0.063	-0.234 ± 0.041	-0.180 ± 0.066	-0.211 ± 0.040	-0.392 ± 0.077	-0.402 ± 0.058	-0.388 ± 0.048	-0.399 ± 0.112
20	0.044 ± 0.020	0.050 ± 0.016	0.039 ± 0.024	0.044 ± 0.018	-0.198 ± 0.067	-0.212 ± 0.045	-0.179 ± 0.068	-0.190 ± 0.028	-0.400 ± 0.081	-0.416 ± 0.062	-0.400 ± 0.051	-0.404 ± 0.115
21	0.040 ± 0.020	0.046 ± 0.016	0.040 ± 0.023	0.040 ± 0.016	-0.177 ± 0.066	-0.185 ± 0.046	-0.176 ± 0.072	-0.165 ± 0.018	-0.408 ± 0.083	-0.429 ± 0.065	-0.412 ± 0.056	-0.409 ± 0.114
22	0.035 ± 0.019	0.042 ± 0.016	0.040 ± 0.022	0.036 ± 0.015	-0.151 ± 0.061	-0.156 ± 0.046	-0.170 ± 0.076	-0.139 ± 0.014	-0.415 ± 0.083	-0.439 ± 0.066	-0.425 ± 0.062	-0.415 ± 0.111
23	0.030 ± 0.018	0.036 ± 0.016	0.039 ± 0.021	0.032 ± 0.013	-0.125 ± 0.053	-0.126 ± 0.044	-0.160 ± 0.079	-0.114 ± 0.016	-0.421 ± 0.079	-0.446 ± 0.066	-0.436 ± 0.069	-0.422 ± 0.107

24	0.025 ± 0.015	0.031 ± 0.015	0.038 ± 0.020	0.028 ± 0.011	-0.100 ± 0.044	-0.096 ± 0.043	-0.148 ± 0.078	-0.090 ± 0.020	-0.428 ± 0.071	-0.448 ± 0.064	-0.445 ± 0.076	-0.430 ± 0.105
25	0.021 ± 0.013	0.026 ± 0.015	0.037 ± 0.019	0.025 ± 0.010	-0.076 ± 0.038	-0.070 ± 0.042	-0.133 ± 0.075	-0.070 ± 0.022	-0.436 ± 0.062	-0.446 ± 0.063	-0.451 ± 0.082	-0.436 ± 0.105
26	0.018 ± 0.011	0.021 ± 0.014	0.035 ± 0.018	0.023 ± 0.010	-0.057 ± 0.036	-0.048 ± 0.042	-0.118 ± 0.069	-0.055 ± 0.022	-0.444 ± 0.052	-0.441 ± 0.064	-0.451 ± 0.086	-0.441 ± 0.108
27	0.017 ± 0.009	0.018 ± 0.013	0.033 ± 0.016	0.022 ± 0.011	-0.043 ± 0.040	-0.031 ± 0.042	-0.102 ± 0.061	-0.044 ± 0.022	-0.452 ± 0.044	-0.432 ± 0.068	-0.447 ± 0.089	-0.442 ± 0.115
28	0.017 ± 0.009	0.016 ± 0.013	0.030 ± 0.015	0.022 ± 0.013	-0.034 ± 0.044	-0.020 ± 0.042	-0.088 ± 0.052	-0.038 ± 0.021	-0.460 ± 0.038	-0.424 ± 0.075	-0.439 ± 0.092	-0.439 ± 0.124
29	0.019 ± 0.009	0.016 ± 0.013	0.028 ± 0.013	0.023 ± 0.014	-0.030 ± 0.047	-0.015 ± 0.041	-0.074 ± 0.043	-0.036 ± 0.022	-0.467 ± 0.037	-0.416 ± 0.085	-0.427 ± 0.095	-0.430 ± 0.135
30	0.022 ± 0.009	0.018 ± 0.013	0.026 ± 0.012	0.025 ± 0.015	-0.031 ± 0.049	-0.015 ± 0.038	-0.063 ± 0.035	-0.037 ± 0.024	-0.471 ± 0.041	-0.409 ± 0.097	-0.415 ± 0.099	-0.418 ± 0.144
31	0.025 ± 0.009	0.020 ± 0.013	0.025 ± 0.012	0.026 ± 0.015	-0.036 ± 0.049	-0.019 ± 0.034	-0.054 ± 0.030	-0.039 ± 0.027	-0.469 ± 0.049	-0.402 ± 0.109	-0.403 ± 0.105	-0.403 ± 0.148
32	0.027 ± 0.009	0.023 ± 0.012	0.023 ± 0.012	0.027 ± 0.015	-0.042 ± 0.047	-0.026 ± 0.030	-0.047 ± 0.028	-0.043 ± 0.029	-0.462 ± 0.058	-0.395 ± 0.118	-0.393 ± 0.112	-0.386 ± 0.145
33	0.028 ± 0.009	0.026 ± 0.012	0.022 ± 0.012	0.027 ± 0.015	-0.048 ± 0.044	-0.033 ± 0.026	-0.044 ± 0.028	-0.046 ± 0.029	-0.448 ± 0.063	-0.387 ± 0.122	-0.385 ± 0.118	-0.368 ± 0.134
34	0.028 ± 0.009	0.027 ± 0.011	0.021 ± 0.013	0.026 ± 0.014	-0.054 ± 0.040	-0.041 ± 0.023	-0.043 ± 0.030	-0.049 ± 0.028	-0.429 ± 0.063	-0.376 ± 0.119	-0.380 ± 0.122	-0.352 ± 0.119
35	0.027 ± 0.009	0.026 ± 0.010	0.021 ± 0.014	0.023 ± 0.012	-0.059 ± 0.036	-0.047 ± 0.021	-0.045 ± 0.033	-0.051 ± 0.026	-0.406 ± 0.058	-0.362 ± 0.111	-0.376 ± 0.124	-0.337 ± 0.101
36	0.025 ± 0.010	0.024 ± 0.010	0.020 ± 0.015	0.020 ± 0.010	-0.062 ± 0.032	-0.052 ± 0.021	-0.048 ± 0.034	-0.053 ± 0.024	-0.381 ± 0.051	-0.346 ± 0.098	-0.372 ± 0.123	-0.325 ± 0.083
37	0.022 ± 0.011	0.022 ± 0.009	0.020 ± 0.015	0.017 ± 0.008	-0.064 ± 0.029	-0.056 ± 0.022	-0.052 ± 0.035	-0.055 ± 0.022	-0.358 ± 0.044	-0.329 ± 0.084	-0.369 ± 0.118	-0.317 ± 0.068
38	0.019 ± 0.011	0.018 ± 0.008	0.019 ± 0.016	0.014 ± 0.008	-0.064 ± 0.027	-0.058 ± 0.023	-0.057 ± 0.035	-0.057 ± 0.021	-0.339 ± 0.039	-0.315 ± 0.070	-0.364 ± 0.109	-0.312 ± 0.057
39	0.016 ± 0.011	0.015 ± 0.007	0.019 ± 0.016	0.012 ± 0.007	-0.065 ± 0.026	-0.059 ± 0.026	-0.060 ± 0.035	-0.059 ± 0.021	-0.324 ± 0.037	-0.305 ± 0.060	-0.360 ± 0.097	-0.310 ± 0.051
40	0.014 ± 0.011	0.012 ± 0.006	0.017 ± 0.015	0.010 ± 0.007	-0.065 ± 0.026	-0.060 ± 0.028	-0.064 ± 0.034	-0.061 ± 0.021	-0.314 ± 0.038	-0.299 ± 0.053	-0.355 ± 0.082	-0.311 ± 0.048
41	0.012 ± 0.010	0.010 ± 0.006	0.015 ± 0.014	0.009 ± 0.006	-0.066 ± 0.027	-0.061 ± 0.030	-0.066 ± 0.032	-0.063 ± 0.021	-0.309 ± 0.041	-0.297 ± 0.048	-0.352 ± 0.064	-0.313 ± 0.046
42	0.010 ± 0.010	0.008 ± 0.005	0.013 ± 0.013	0.008 ± 0.006	-0.066 ± 0.029	-0.061 ± 0.032	-0.067 ± 0.031	-0.064 ± 0.022	-0.308 ± 0.043	-0.300 ± 0.043	-0.351 ± 0.046	-0.316 ± 0.044
43	0.008 ± 0.009	0.007 ± 0.005	0.011 ± 0.011	0.007 ± 0.006	-0.067 ± 0.030	-0.061 ± 0.034	-0.069 ± 0.030	-0.064 ± 0.024	-0.311 ± 0.044	-0.307 ± 0.036	-0.352 ± 0.034	-0.320 ± 0.040

44	0.007 ± 0.009	0.006 ± 0.005	0.009 ± 0.010	0.006 ± 0.005	-0.068 ± 0.032	-0.061 ± 0.036	-0.070 ± 0.029	-0.064 ± 0.025	-0.317 ± 0.043	-0.318 ± 0.028	-0.357 ± 0.040	-0.326 ± 0.035
45	0.006 ± 0.008	0.005 ± 0.004	0.008 ± 0.009	0.005 ± 0.005	-0.068 ± 0.033	-0.060 ± 0.038	-0.071 ± 0.029	-0.064 ± 0.027	-0.325 ± 0.042	-0.331 ± 0.023	-0.366 ± 0.059	-0.333 ± 0.035
46	0.005 ± 0.007	0.004 ± 0.004	0.007 ± 0.008	0.004 ± 0.005	-0.069 ± 0.033	-0.059 ± 0.039	-0.072 ± 0.030	-0.063 ± 0.027	-0.335 ± 0.041	-0.348 ± 0.028	-0.379 ± 0.086	-0.343 ± 0.041
47	0.005 ± 0.007	0.003 ± 0.004	0.006 ± 0.008	0.003 ± 0.005	-0.069 ± 0.034	-0.058 ± 0.040	-0.073 ± 0.031	-0.063 ± 0.028	-0.348 ± 0.044	-0.368 ± 0.044	-0.396 ± 0.118	-0.357 ± 0.054
48	0.004 ± 0.006	0.003 ± 0.005	0.006 ± 0.007	0.002 ± 0.005	-0.069 ± 0.033	-0.057 ± 0.041	-0.074 ± 0.032	-0.062 ± 0.028	-0.363 ± 0.052	-0.393 ± 0.066	-0.416 ± 0.155	-0.376 ± 0.073
49	0.004 ± 0.006	0.003 ± 0.005	0.006 ± 0.007	0.002 ± 0.005	-0.070 ± 0.033	-0.057 ± 0.042	-0.075 ± 0.033	-0.062 ± 0.027	-0.381 ± 0.063	-0.424 ± 0.095	-0.441 ± 0.198	-0.401 ± 0.097
50	0.004 ± 0.006	0.003 ± 0.005	0.006 ± 0.007	0.002 ± 0.005	-0.070 ± 0.033	-0.056 ± 0.043	-0.077 ± 0.034	-0.063 ± 0.026	-0.402 ± 0.078	-0.462 ± 0.129	-0.471 ± 0.245	-0.432 ± 0.125
51	0.004 ± 0.006	0.003 ± 0.005	0.007 ± 0.008	0.002 ± 0.005	-0.070 ± 0.034	-0.056 ± 0.045	-0.079 ± 0.035	-0.064 ± 0.024	-0.428 ± 0.096	-0.506 ± 0.169	-0.506 ± 0.293	-0.471 ± 0.156
52	0.005 ± 0.006	0.004 ± 0.005	0.007 ± 0.008	0.002 ± 0.005	-0.071 ± 0.036	-0.056 ± 0.048	-0.081 ± 0.037	-0.065 ± 0.023	-0.459 ± 0.117	-0.556 ± 0.212	-0.544 ± 0.337	-0.518 ± 0.191
53	0.006 ± 0.007	0.004 ± 0.004	0.007 ± 0.009	0.003 ± 0.005	-0.072 ± 0.039	-0.057 ± 0.051	-0.084 ± 0.039	-0.066 ± 0.022	-0.497 ± 0.141	-0.609 ± 0.255	-0.583 ± 0.373	-0.571 ± 0.230
54	0.007 ± 0.007	0.005 ± 0.004	0.006 ± 0.008	0.003 ± 0.005	-0.075 ± 0.043	-0.059 ± 0.055	-0.087 ± 0.042	-0.068 ± 0.022	-0.541 ± 0.167	-0.665 ± 0.295	-0.622 ± 0.394	-0.632 ± 0.274
55	0.008 ± 0.008	0.006 ± 0.005	0.006 ± 0.007	0.004 ± 0.005	-0.078 ± 0.048	-0.062 ± 0.060	-0.090 ± 0.044	-0.071 ± 0.023	-0.590 ± 0.193	-0.721 ± 0.329	-0.655 ± 0.398	-0.698 ± 0.324
56	0.010 ± 0.009	0.007 ± 0.007	0.006 ± 0.007	0.005 ± 0.006	-0.083 ± 0.054	-0.067 ± 0.066	-0.094 ± 0.047	-0.074 ± 0.025	-0.641 ± 0.214	-0.773 ± 0.358	-0.681 ± 0.384	-0.765 ± 0.377
57	0.012 ± 0.011	0.009 ± 0.010	0.007 ± 0.010	0.007 ± 0.008	-0.089 ± 0.062	-0.073 ± 0.072	-0.099 ± 0.049	-0.080 ± 0.026	-0.693 ± 0.227	-0.818 ± 0.381	-0.696 ± 0.361	-0.828 ± 0.427
58	0.014 ± 0.012	0.011 ± 0.013	0.008 ± 0.014	0.009 ± 0.012	-0.096 ± 0.070	-0.081 ± 0.078	-0.104 ± 0.052	-0.086 ± 0.029	-0.741 ± 0.231	-0.854 ± 0.397	-0.702 ± 0.338	-0.883 ± 0.467
59	0.016 ± 0.014	0.013 ± 0.018	0.010 ± 0.018	0.012 ± 0.016	-0.104 ± 0.077	-0.089 ± 0.083	-0.110 ± 0.055	-0.094 ± 0.031	-0.783 ± 0.227	-0.875 ± 0.404	-0.698 ± 0.321	-0.920 ± 0.488
60	0.018 ± 0.016	0.015 ± 0.021	0.013 ± 0.021	0.015 ± 0.021	-0.112 ± 0.084	-0.098 ± 0.085	-0.118 ± 0.059	-0.103 ± 0.035	-0.816 ± 0.221	-0.879 ± 0.400	-0.685 ± 0.311	-0.935 ± 0.483
61	0.019 ± 0.018	0.017 ± 0.023	0.016 ± 0.023	0.018 ± 0.025	-0.120 ± 0.090	-0.107 ± 0.084	-0.125 ± 0.063	-0.113 ± 0.040	-0.839 ± 0.218	-0.863 ± 0.383	-0.667 ± 0.301	-0.924 ± 0.451
62	0.021 ± 0.020	0.018 ± 0.024	0.019 ± 0.024	0.019 ± 0.028	-0.128 ± 0.095	-0.116 ± 0.080	-0.133 ± 0.067	-0.121 ± 0.046	-0.847 ± 0.224	-0.827 ± 0.354	-0.644 ± 0.286	-0.886 ± 0.395
63	0.022 ± 0.022	0.018 ± 0.022	0.021 ± 0.024	0.020 ± 0.028	-0.135 ± 0.097	-0.124 ± 0.076	-0.140 ± 0.071	-0.129 ± 0.051	-0.840 ± 0.236	-0.773 ± 0.317	-0.618 ± 0.266	-0.826 ± 0.327

64	0.022 ± 0.023	0.017 ± 0.018	0.022 ± 0.025	0.020 ± 0.025	-0.141 ± 0.097	-0.132 ± 0.071	-0.148 ± 0.075	-0.135 ± 0.055	-0.817 ± 0.248	-0.705 ± 0.275	-0.589 ± 0.240	-0.750 ± 0.258
65	0.023 ± 0.023	0.016 ± 0.015	0.023 ± 0.025	0.020 ± 0.020	-0.147 ± 0.096	-0.140 ± 0.069	-0.155 ± 0.081	-0.140 ± 0.057	-0.778 ± 0.255	-0.631 ± 0.231	-0.559 ± 0.213	-0.668 ± 0.202
66	0.024 ± 0.023	0.016 ± 0.014	0.023 ± 0.025	0.019 ± 0.016	-0.153 ± 0.094	-0.148 ± 0.069	-0.162 ± 0.086	-0.146 ± 0.059	-0.726 ± 0.253	-0.559 ± 0.188	-0.529 ± 0.185	-0.588 ± 0.163
67	0.025 ± 0.023	0.017 ± 0.015	0.025 ± 0.025	0.019 ± 0.014	-0.161 ± 0.091	-0.158 ± 0.069	-0.169 ± 0.091	-0.154 ± 0.061	-0.664 ± 0.240	-0.494 ± 0.148	-0.499 ± 0.158	-0.517 ± 0.140
68	0.027 ± 0.024	0.020 ± 0.016	0.027 ± 0.025	0.021 ± 0.016	-0.171 ± 0.088	-0.169 ± 0.068	-0.178 ± 0.094	-0.165 ± 0.063	-0.599 ± 0.216	-0.442 ± 0.110	-0.473 ± 0.131	-0.461 ± 0.123
69	0.030 ± 0.024	0.026 ± 0.016	0.030 ± 0.024	0.025 ± 0.018	-0.184 ± 0.087	-0.183 ± 0.066	-0.187 ± 0.093	-0.180 ± 0.066	-0.536 ± 0.185	-0.403 ± 0.080	-0.449 ± 0.104	-0.420 ± 0.105
70	0.034 ± 0.025	0.032 ± 0.014	0.035 ± 0.023	0.031 ± 0.019	-0.200 ± 0.086	-0.200 ± 0.060	-0.198 ± 0.088	-0.200 ± 0.067	-0.480 ± 0.148	-0.376 ± 0.061	-0.430 ± 0.077	-0.391 ± 0.086
71	0.039 ± 0.026	0.038 ± 0.012	0.039 ± 0.021	0.038 ± 0.019	-0.219 ± 0.085	-0.218 ± 0.051	-0.211 ± 0.080	-0.222 ± 0.068	-0.435 ± 0.109	-0.359 ± 0.053	-0.415 ± 0.052	-0.372 ± 0.068
72	0.045 ± 0.026	0.045 ± 0.009	0.044 ± 0.019	0.045 ± 0.018	-0.241 ± 0.083	-0.236 ± 0.041	-0.226 ± 0.071	-0.246 ± 0.067	-0.402 ± 0.072	-0.350 ± 0.052	-0.404 ± 0.032	-0.360 ± 0.053
73	0.051 ± 0.026	0.050 ± 0.009	0.048 ± 0.017	0.052 ± 0.017	-0.263 ± 0.080	-0.254 ± 0.033	-0.241 ± 0.062	-0.267 ± 0.065	-0.378 ± 0.042	-0.346 ± 0.052	-0.395 ± 0.024	-0.353 ± 0.043
74	0.056 ± 0.025	0.055 ± 0.011	0.052 ± 0.016	0.057 ± 0.017	-0.283 ± 0.075	-0.270 ± 0.037	-0.256 ± 0.059	-0.286 ± 0.063	-0.364 ± 0.027	-0.345 ± 0.051	-0.388 ± 0.028	-0.350 ± 0.037
75	0.060 ± 0.023	0.058 ± 0.014	0.056 ± 0.017	0.061 ± 0.016	-0.301 ± 0.067	-0.281 ± 0.052	-0.269 ± 0.063	-0.299 ± 0.059	-0.356 ± 0.031	-0.349 ± 0.051	-0.383 ± 0.036	-0.350 ± 0.035
76	0.063 ± 0.020	0.059 ± 0.017	0.058 ± 0.019	0.063 ± 0.016	-0.314 ± 0.058	-0.287 ± 0.071	-0.280 ± 0.074	-0.305 ± 0.056	-0.352 ± 0.039	-0.357 ± 0.054	-0.379 ± 0.043	-0.354 ± 0.035
77	0.065 ± 0.017	0.060 ± 0.020	0.060 ± 0.022	0.063 ± 0.016	-0.321 ± 0.050	-0.287 ± 0.090	-0.286 ± 0.086	-0.305 ± 0.055	-0.352 ± 0.045	-0.367 ± 0.063	-0.379 ± 0.050	-0.364 ± 0.040
78	0.065 ± 0.015	0.060 ± 0.022	0.060 ± 0.025	0.063 ± 0.017	-0.321 ± 0.045	-0.281 ± 0.105	-0.287 ± 0.098	-0.298 ± 0.056	-0.356 ± 0.049	-0.378 ± 0.072	-0.382 ± 0.056	-0.378 ± 0.049
79	0.064 ± 0.013	0.059 ± 0.024	0.060 ± 0.027	0.060 ± 0.017	-0.313 ± 0.045	-0.270 ± 0.115	-0.282 ± 0.107	-0.283 ± 0.059	-0.363 ± 0.055	-0.387 ± 0.078	-0.388 ± 0.063	-0.395 ± 0.062
80	0.061 ± 0.013	0.056 ± 0.024	0.058 ± 0.028	0.057 ± 0.018	-0.299 ± 0.052	-0.253 ± 0.118	-0.271 ± 0.113	-0.262 ± 0.062	-0.374 ± 0.062	-0.395 ± 0.078	-0.397 ± 0.071	-0.415 ± 0.080
81	0.058 ± 0.014	0.053 ± 0.023	0.055 ± 0.029	0.052 ± 0.018	-0.278 ± 0.063	-0.230 ± 0.115	-0.254 ± 0.113	-0.236 ± 0.066	-0.389 ± 0.072	-0.400 ± 0.073	-0.407 ± 0.077	-0.434 ± 0.101
82	0.053 ± 0.016	0.048 ± 0.021	0.051 ± 0.028	0.046 ± 0.018	-0.252 ± 0.074	-0.204 ± 0.107	-0.233 ± 0.109	-0.205 ± 0.069	-0.406 ± 0.083	-0.404 ± 0.065	-0.417 ± 0.081	-0.451 ± 0.120
83	0.048 ± 0.018	0.043 ± 0.018	0.046 ± 0.027	0.040 ± 0.017	-0.221 ± 0.083	-0.174 ± 0.095	-0.209 ± 0.100	-0.173 ± 0.069	-0.425 ± 0.094	-0.409 ± 0.056	-0.426 ± 0.080	-0.465 ± 0.132

84	0.042 ± 0.019	0.036 ± 0.015	0.041 ± 0.023	0.034 ± 0.016	-0.188 ± 0.088	-0.142 ± 0.081	-0.182 ± 0.087	-0.140 ± 0.066	-0.444 ± 0.103	-0.418 ± 0.046	-0.435 ± 0.074	-0.476 ± 0.133
85	0.036 ± 0.019	0.030 ± 0.012	0.037 ± 0.019	0.028 ± 0.014	-0.155 ± 0.088	-0.109 ± 0.067	-0.155 ± 0.072	-0.109 ± 0.061	-0.462 ± 0.105	-0.431 ± 0.038	-0.443 ± 0.065	-0.483 ± 0.121
86	0.031 ± 0.018	0.023 ± 0.010	0.032 ± 0.014	0.023 ± 0.011	-0.123 ± 0.082	-0.076 ± 0.054	-0.128 ± 0.057	-0.081 ± 0.052	-0.479 ± 0.098	-0.449 ± 0.037	-0.452 ± 0.054	-0.488 ± 0.097
87	0.026 ± 0.016	0.018 ± 0.008	0.028 ± 0.010	0.020 ± 0.009	-0.094 ± 0.070	-0.047 ± 0.043	-0.102 ± 0.043	-0.058 ± 0.040	-0.494 ± 0.082	-0.470 ± 0.048	-0.461 ± 0.044	-0.490 ± 0.066
88	0.023 ± 0.013	0.013 ± 0.006	0.025 ± 0.006	0.018 ± 0.006	-0.068 ± 0.055	-0.023 ± 0.037	-0.079 ± 0.034	-0.040 ± 0.029	-0.509 ± 0.062	-0.490 ± 0.066	-0.471 ± 0.037	-0.491 ± 0.048
89	0.021 ± 0.010	0.011 ± 0.006	0.022 ± 0.005	0.017 ± 0.003	-0.047 ± 0.037	-0.004 ± 0.037	-0.059 ± 0.029	-0.027 ± 0.020	-0.523 ± 0.047	-0.504 ± 0.084	-0.479 ± 0.042	-0.489 ± 0.064
90	0.019 ± 0.008	0.010 ± 0.006	0.020 ± 0.004	0.016 ± 0.003	-0.031 ± 0.024	0.007 ± 0.042	-0.043 ± 0.029	-0.018 ± 0.020	-0.533 ± 0.053	-0.509 ± 0.099	-0.485 ± 0.058	-0.484 ± 0.092
91	0.018 ± 0.009	0.010 ± 0.007	0.018 ± 0.003	0.016 ± 0.005	-0.018 ± 0.025	0.012 ± 0.048	-0.031 ± 0.031	-0.013 ± 0.026	-0.536 ± 0.072	-0.503 ± 0.106	-0.485 ± 0.080	-0.473 ± 0.115
92	0.017 ± 0.010	0.012 ± 0.009	0.016 ± 0.005	0.016 ± 0.007	-0.009 ± 0.038	0.010 ± 0.052	-0.023 ± 0.035	-0.012 ± 0.033	-0.532 ± 0.092	-0.486 ± 0.104	-0.480 ± 0.101	-0.456 ± 0.127
93	0.016 ± 0.011	0.014 ± 0.010	0.016 ± 0.007	0.016 ± 0.009	-0.004 ± 0.050	0.004 ± 0.051	-0.019 ± 0.040	-0.015 ± 0.037	-0.516 ± 0.106	-0.458 ± 0.095	-0.470 ± 0.118	-0.434 ± 0.129
94	0.015 ± 0.011	0.016 ± 0.011	0.015 ± 0.010	0.016 ± 0.010	-0.002 ± 0.059	-0.006 ± 0.046	-0.018 ± 0.045	-0.020 ± 0.038	-0.489 ± 0.111	-0.423 ± 0.084	-0.454 ± 0.127	-0.407 ± 0.123
95	0.013 ± 0.011	0.018 ± 0.012	0.015 ± 0.012	0.017 ± 0.011	-0.003 ± 0.063	-0.018 ± 0.038	-0.021 ± 0.050	-0.026 ± 0.036	-0.453 ± 0.109	-0.384 ± 0.081	-0.433 ± 0.128	-0.377 ± 0.110
96	0.012 ± 0.010	0.019 ± 0.012	0.015 ± 0.014	0.016 ± 0.011	-0.008 ± 0.062	-0.029 ± 0.029	-0.026 ± 0.052	-0.034 ± 0.032	-0.412 ± 0.100	-0.347 ± 0.087	-0.409 ± 0.120	-0.347 ± 0.095
97	0.012 ± 0.008	0.019 ± 0.013	0.015 ± 0.013	0.016 ± 0.011	-0.016 ± 0.057	-0.038 ± 0.020	-0.033 ± 0.050	-0.042 ± 0.027	-0.371 ± 0.085	-0.317 ± 0.096	-0.381 ± 0.102	-0.321 ± 0.080
98	0.012 ± 0.007	0.018 ± 0.012	0.015 ± 0.012	0.014 ± 0.010	-0.026 ± 0.046	-0.045 ± 0.014	-0.042 ± 0.043	-0.048 ± 0.021	-0.335 ± 0.061	-0.296 ± 0.095	-0.350 ± 0.074	-0.299 ± 0.067
99	0.014 ± 0.008	0.014 ± 0.009	0.014 ± 0.010	0.012 ± 0.009	-0.040 ± 0.032	-0.046 ± 0.010	-0.051 ± 0.031	-0.053 ± 0.016	-0.308 ± 0.044	-0.291 ± 0.084	-0.317 ± 0.048	-0.286 ± 0.055
100	0.017 ± 0.011	0.008 ± 0.004	0.012 ± 0.009	0.009 ± 0.011	-0.056 ± 0.020	-0.042 ± 0.014	-0.061 ± 0.018	-0.055 ± 0.012	-0.293 ± 0.078	-0.304 ± 0.077	-0.282 ± 0.076	-0.282 ± 0.053

Tab. S3 Full normalized knee joint reaction force data during jogging under four knee protector conditions (group mean ± SD, n = 5).

Percent cycle (%)	Fx/BW Pro.off	Fx/BW Pro.a	Fx/BW Pro.b	Fx/BW Pro.c	Fy/BW Pro.off	Fy/BW Pro.a	Fy/BW Pro.b	Fy/BW Pro.c	Fz/BW Pro.of	Fz/BW Pro.a	Fz/BW Pro.b	Fz/BW Pro.c

									f			
0	0.013 ± 0.009	0.021 ± 0.019	0.022 ± 0.027	0.021 ± 0.017	-0.205 ± 0.049	-0.201 ± 0.069	-0.219 ± 0.076	-0.234 ± 0.058	-0.436 ± 0.052	-0.480 ± 0.044	-0.508 ± 0.046	-0.493 ± 0.046
1	0.013 ± 0.009	0.021 ± 0.018	0.022 ± 0.026	0.021 ± 0.016	-0.204 ± 0.047	-0.200 ± 0.069	-0.219 ± 0.074	-0.232 ± 0.058	-0.435 ± 0.050	-0.478 ± 0.044	-0.506 ± 0.046	-0.491 ± 0.046
2	0.013 ± 0.009	0.021 ± 0.018	0.022 ± 0.025	0.021 ± 0.016	-0.203 ± 0.046	-0.199 ± 0.068	-0.219 ± 0.072	-0.231 ± 0.057	-0.434 ± 0.049	-0.475 ± 0.044	-0.504 ± 0.045	-0.488 ± 0.046
3	0.013 ± 0.008	0.021 ± 0.018	0.023 ± 0.025	0.022 ± 0.016	-0.202 ± 0.044	-0.198 ± 0.067	-0.219 ± 0.070	-0.230 ± 0.056	-0.433 ± 0.048	-0.473 ± 0.043	-0.501 ± 0.045	-0.486 ± 0.047
4	0.014 ± 0.008	0.021 ± 0.017	0.023 ± 0.024	0.022 ± 0.015	-0.201 ± 0.042	-0.197 ± 0.066	-0.219 ± 0.068	-0.228 ± 0.056	-0.432 ± 0.047	-0.470 ± 0.043	-0.499 ± 0.044	-0.483 ± 0.047
5	0.014 ± 0.008	0.021 ± 0.017	0.023 ± 0.024	0.022 ± 0.015	-0.200 ± 0.040	-0.196 ± 0.066	-0.218 ± 0.066	-0.227 ± 0.055	-0.431 ± 0.046	-0.468 ± 0.042	-0.497 ± 0.044	-0.481 ± 0.047
6	0.014 ± 0.008	0.021 ± 0.017	0.023 ± 0.023	0.022 ± 0.015	-0.199 ± 0.039	-0.195 ± 0.065	-0.218 ± 0.065	-0.226 ± 0.054	-0.430 ± 0.045	-0.465 ± 0.042	-0.495 ± 0.043	-0.478 ± 0.047
7	0.014 ± 0.007	0.021 ± 0.016	0.023 ± 0.022	0.023 ± 0.015	-0.198 ± 0.037	-0.194 ± 0.064	-0.218 ± 0.063	-0.225 ± 0.054	-0.430 ± 0.044	-0.463 ± 0.041	-0.492 ± 0.043	-0.476 ± 0.047
8	0.015 ± 0.007	0.022 ± 0.016	0.024 ± 0.022	0.023 ± 0.014	-0.198 ± 0.035	-0.193 ± 0.063	-0.217 ± 0.061	-0.224 ± 0.053	-0.429 ± 0.043	-0.460 ± 0.041	-0.490 ± 0.042	-0.473 ± 0.047
9	0.015 ± 0.007	0.022 ± 0.016	0.024 ± 0.021	0.023 ± 0.014	-0.197 ± 0.034	-0.192 ± 0.062	-0.217 ± 0.060	-0.222 ± 0.052	-0.428 ± 0.042	-0.458 ± 0.040	-0.487 ± 0.041	-0.471 ± 0.046
10	0.015 ± 0.007	0.022 ± 0.015	0.024 ± 0.021	0.023 ± 0.014	-0.196 ± 0.032	-0.191 ± 0.061	-0.217 ± 0.058	-0.221 ± 0.052	-0.427 ± 0.041	-0.455 ± 0.040	-0.485 ± 0.041	-0.468 ± 0.046
11	0.015 ± 0.007	0.022 ± 0.015	0.024 ± 0.020	0.024 ± 0.014	-0.196 ± 0.031	-0.190 ± 0.060	-0.217 ± 0.056	-0.220 ± 0.051	-0.427 ± 0.040	-0.453 ± 0.039	-0.482 ± 0.040	-0.466 ± 0.046
12	0.016 ± 0.007	0.022 ± 0.015	0.024 ± 0.020	0.024 ± 0.014	-0.195 ± 0.029	-0.190 ± 0.059	-0.216 ± 0.055	-0.219 ± 0.050	-0.426 ± 0.039	-0.450 ± 0.038	-0.480 ± 0.039	-0.463 ± 0.046
13	0.016 ± 0.007	0.022 ± 0.014	0.025 ± 0.019	0.024 ± 0.014	-0.195 ± 0.028	-0.189 ± 0.058	-0.216 ± 0.053	-0.218 ± 0.049	-0.425 ± 0.038	-0.448 ± 0.037	-0.477 ± 0.038	-0.461 ± 0.045
14	0.016 ± 0.006	0.023 ± 0.014	0.025 ± 0.019	0.025 ± 0.014	-0.194 ± 0.027	-0.188 ± 0.057	-0.216 ± 0.052	-0.217 ± 0.049	-0.425 ± 0.038	-0.445 ± 0.037	-0.475 ± 0.037	-0.458 ± 0.045
15	0.017 ± 0.006	0.023 ± 0.014	0.025 ± 0.018	0.025 ± 0.014	-0.194 ± 0.025	-0.187 ± 0.056	-0.215 ± 0.051	-0.216 ± 0.048	-0.424 ± 0.037	-0.443 ± 0.036	-0.472 ± 0.036	-0.456 ± 0.045
16	0.017 ± 0.007	0.023 ± 0.014	0.025 ± 0.018	0.025 ± 0.013	-0.194 ± 0.024	-0.187 ± 0.055	-0.215 ± 0.050	-0.216 ± 0.048	-0.423 ± 0.036	-0.440 ± 0.035	-0.470 ± 0.035	-0.453 ± 0.044
17	0.018 ± 0.007	0.023 ± 0.013	0.025 ± 0.018	0.026 ± 0.013	-0.193 ± 0.023	-0.186 ± 0.054	-0.215 ± 0.049	-0.215 ± 0.047	-0.423 ± 0.035	-0.438 ± 0.034	-0.467 ± 0.034	-0.451 ± 0.044
18	0.018 ± 0.007	0.023 ± 0.013	0.026 ± 0.017	0.026 ± 0.013	-0.193 ± 0.023	-0.186 ± 0.053	-0.215 ± 0.048	-0.214 ± 0.046	-0.422 ± 0.035	-0.435 ± 0.033	-0.464 ± 0.033	-0.449 ± 0.043
19	0.018 ±	0.024 ±	0.026 ±	0.026 ±	-0.193 ±	-0.186 ±	-0.214 ±	-0.214 ±	-0.421 ±	-0.433 ±	-0.462 ±	-0.446 ±

	0.007	0.013	0.017	0.013	0.022	0.052	0.047	0.046	0.034	0.032	0.032	0.043
20	0.019 ± 0.007	0.024 ± 0.013	0.026 ± 0.017	0.027 ± 0.013	-0.194 ± 0.022	-0.186 ± 0.051	-0.215 ± 0.046	-0.214 ± 0.045	-0.421 ± 0.034	-0.430 ± 0.031	-0.459 ± 0.031	-0.444 ± 0.042
21	0.019 ± 0.007	0.025 ± 0.013	0.027 ± 0.016	0.027 ± 0.013	-0.194 ± 0.021	-0.186 ± 0.050	-0.215 ± 0.045	-0.213 ± 0.045	-0.420 ± 0.033	-0.428 ± 0.030	-0.456 ± 0.030	-0.441 ± 0.041
22	0.020 ± 0.007	0.025 ± 0.012	0.027 ± 0.016	0.028 ± 0.013	-0.194 ± 0.021	-0.186 ± 0.050	-0.215 ± 0.045	-0.213 ± 0.045	-0.419 ± 0.032	-0.425 ± 0.029	-0.453 ± 0.030	-0.439 ± 0.041
23	0.021 ± 0.007	0.025 ± 0.012	0.028 ± 0.016	0.028 ± 0.013	-0.195 ± 0.021	-0.187 ± 0.049	-0.215 ± 0.044	-0.214 ± 0.045	-0.418 ± 0.032	-0.423 ± 0.028	-0.451 ± 0.029	-0.436 ± 0.040
24	0.021 ± 0.007	0.026 ± 0.012	0.028 ± 0.015	0.029 ± 0.013	-0.196 ± 0.022	-0.187 ± 0.048	-0.216 ± 0.044	-0.214 ± 0.044	-0.417 ± 0.031	-0.420 ± 0.027	-0.448 ± 0.028	-0.434 ± 0.039
25	0.022 ± 0.008	0.026 ± 0.012	0.029 ± 0.015	0.030 ± 0.013	-0.196 ± 0.022	-0.188 ± 0.048	-0.216 ± 0.043	-0.214 ± 0.044	-0.417 ± 0.031	-0.418 ± 0.026	-0.445 ± 0.027	-0.431 ± 0.038
26	0.023 ± 0.008	0.027 ± 0.012	0.029 ± 0.015	0.030 ± 0.013	-0.197 ± 0.023	-0.189 ± 0.047	-0.217 ± 0.043	-0.215 ± 0.044	-0.416 ± 0.030	-0.415 ± 0.025	-0.442 ± 0.026	-0.429 ± 0.038
27	0.023 ± 0.008	0.028 ± 0.012	0.030 ± 0.015	0.031 ± 0.013	-0.198 ± 0.023	-0.190 ± 0.047	-0.218 ± 0.043	-0.216 ± 0.044	-0.415 ± 0.030	-0.413 ± 0.024	-0.440 ± 0.025	-0.427 ± 0.037
28	0.024 ± 0.008	0.028 ± 0.012	0.030 ± 0.014	0.032 ± 0.013	-0.200 ± 0.024	-0.192 ± 0.047	-0.219 ± 0.043	-0.217 ± 0.044	-0.414 ± 0.029	-0.411 ± 0.023	-0.437 ± 0.025	-0.424 ± 0.036
29	0.025 ± 0.008	0.029 ± 0.012	0.031 ± 0.014	0.032 ± 0.013	-0.201 ± 0.025	-0.193 ± 0.047	-0.220 ± 0.043	-0.218 ± 0.044	-0.413 ± 0.029	-0.409 ± 0.022	-0.434 ± 0.024	-0.422 ± 0.035
30	0.026 ± 0.008	0.030 ± 0.012	0.031 ± 0.014	0.033 ± 0.013	-0.202 ± 0.026	-0.195 ± 0.046	-0.222 ± 0.043	-0.219 ± 0.044	-0.412 ± 0.028	-0.406 ± 0.021	-0.432 ± 0.024	-0.420 ± 0.034
31	0.027 ± 0.008	0.030 ± 0.011	0.032 ± 0.014	0.034 ± 0.013	-0.204 ± 0.026	-0.197 ± 0.046	-0.223 ± 0.043	-0.221 ± 0.044	-0.411 ± 0.028	-0.404 ± 0.020	-0.429 ± 0.024	-0.418 ± 0.034
32	0.028 ± 0.008	0.031 ± 0.011	0.033 ± 0.014	0.035 ± 0.013	-0.206 ± 0.027	-0.199 ± 0.046	-0.225 ± 0.043	-0.222 ± 0.044	-0.411 ± 0.027	-0.402 ± 0.020	-0.427 ± 0.023	-0.416 ± 0.033
33	0.029 ± 0.008	0.032 ± 0.011	0.034 ± 0.014	0.036 ± 0.013	-0.208 ± 0.028	-0.201 ± 0.046	-0.227 ± 0.043	-0.224 ± 0.044	-0.410 ± 0.027	-0.401 ± 0.019	-0.424 ± 0.023	-0.414 ± 0.032
34	0.030 ± 0.008	0.033 ± 0.011	0.034 ± 0.013	0.036 ± 0.013	-0.210 ± 0.028	-0.204 ± 0.046	-0.229 ± 0.043	-0.226 ± 0.044	-0.409 ± 0.027	-0.399 ± 0.019	-0.422 ± 0.024	-0.412 ± 0.032
35	0.031 ± 0.008	0.034 ± 0.011	0.035 ± 0.013	0.037 ± 0.013	-0.212 ± 0.029	-0.206 ± 0.046	-0.231 ± 0.043	-0.228 ± 0.044	-0.408 ± 0.027	-0.397 ± 0.019	-0.420 ± 0.024	-0.410 ± 0.032
36	0.032 ± 0.008	0.035 ± 0.011	0.036 ± 0.013	0.038 ± 0.013	-0.214 ± 0.029	-0.209 ± 0.046	-0.233 ± 0.043	-0.231 ± 0.044	-0.407 ± 0.027	-0.396 ± 0.019	-0.417 ± 0.024	-0.409 ± 0.031
37	0.033 ± 0.008	0.036 ± 0.011	0.037 ± 0.013	0.039 ± 0.013	-0.217 ± 0.030	-0.212 ± 0.046	-0.235 ± 0.043	-0.233 ± 0.044	-0.406 ± 0.028	-0.394 ± 0.020	-0.415 ± 0.025	-0.407 ± 0.031
38	0.034 ± 0.008	0.036 ± 0.011	0.038 ± 0.013	0.040 ± 0.012	-0.219 ± 0.030	-0.215 ± 0.046	-0.238 ± 0.043	-0.235 ± 0.044	-0.405 ± 0.028	-0.393 ± 0.021	-0.414 ± 0.025	-0.406 ± 0.031
39	0.035 ±	0.037 ±	0.038 ±	0.041 ±	-0.222 ±	-0.218 ±	-0.241 ±	-0.238 ±	-0.405 ±	-0.392 ±	-0.412 ±	-0.405 ±

	0.008	0.011	0.013	0.012	0.030	0.046	0.044	0.044	0.029	0.022	0.026	0.031
40	0.036 ± 0.008	0.038 ± 0.011	0.039 ± 0.013	0.042 ± 0.012	-0.224 ± 0.030	-0.221 ± 0.046	-0.243 ± 0.044	-0.241 ± 0.043	-0.404 ± 0.030	-0.391 ± 0.023	-0.410 ± 0.027	-0.404 ± 0.031
41	0.037 ± 0.008	0.039 ± 0.010	0.040 ± 0.012	0.043 ± 0.012	-0.227 ± 0.030	-0.225 ± 0.046	-0.246 ± 0.044	-0.243 ± 0.043	-0.404 ± 0.031	-0.391 ± 0.025	-0.409 ± 0.028	-0.403 ± 0.032
42	0.038 ± 0.008	0.040 ± 0.010	0.041 ± 0.012	0.044 ± 0.012	-0.230 ± 0.030	-0.228 ± 0.046	-0.249 ± 0.044	-0.246 ± 0.043	-0.403 ± 0.032	-0.390 ± 0.026	-0.408 ± 0.029	-0.402 ± 0.032
43	0.039 ± 0.008	0.041 ± 0.010	0.042 ± 0.012	0.045 ± 0.012	-0.232 ± 0.030	-0.231 ± 0.046	-0.252 ± 0.045	-0.249 ± 0.043	-0.403 ± 0.033	-0.390 ± 0.028	-0.407 ± 0.030	-0.402 ± 0.032
44	0.040 ± 0.007	0.042 ± 0.010	0.043 ± 0.012	0.045 ± 0.012	-0.235 ± 0.029	-0.235 ± 0.046	-0.255 ± 0.045	-0.252 ± 0.043	-0.403 ± 0.035	-0.390 ± 0.030	-0.406 ± 0.031	-0.402 ± 0.033
45	0.041 ± 0.007	0.043 ± 0.010	0.044 ± 0.012	0.046 ± 0.012	-0.238 ± 0.029	-0.238 ± 0.046	-0.258 ± 0.045	-0.255 ± 0.042	-0.403 ± 0.036	-0.390 ± 0.032	-0.406 ± 0.032	-0.402 ± 0.034
46	0.042 ± 0.007	0.044 ± 0.010	0.045 ± 0.012	0.047 ± 0.012	-0.241 ± 0.028	-0.242 ± 0.046	-0.261 ± 0.046	-0.258 ± 0.042	-0.403 ± 0.038	-0.391 ± 0.034	-0.406 ± 0.034	-0.402 ± 0.034
47	0.043 ± 0.007	0.045 ± 0.010	0.046 ± 0.012	0.048 ± 0.012	-0.244 ± 0.028	-0.245 ± 0.045	-0.264 ± 0.046	-0.260 ± 0.042	-0.403 ± 0.040	-0.392 ± 0.036	-0.406 ± 0.035	-0.403 ± 0.035
48	0.044 ± 0.006	0.046 ± 0.009	0.046 ± 0.012	0.049 ± 0.012	-0.247 ± 0.027	-0.249 ± 0.045	-0.267 ± 0.046	-0.263 ± 0.042	-0.404 ± 0.042	-0.393 ± 0.039	-0.406 ± 0.036	-0.404 ± 0.036
49	0.045 ± 0.006	0.047 ± 0.009	0.047 ± 0.012	0.050 ± 0.012	-0.249 ± 0.026	-0.252 ± 0.045	-0.270 ± 0.047	-0.266 ± 0.042	-0.404 ± 0.044	-0.394 ± 0.041	-0.407 ± 0.037	-0.405 ± 0.037
50	0.046 ± 0.006	0.048 ± 0.009	0.048 ± 0.012	0.050 ± 0.012	-0.252 ± 0.026	-0.255 ± 0.045	-0.273 ± 0.048	-0.268 ± 0.042	-0.405 ± 0.045	-0.396 ± 0.043	-0.408 ± 0.039	-0.407 ± 0.038
51	0.047 ± 0.005	0.049 ± 0.009	0.049 ± 0.012	0.051 ± 0.012	-0.254 ± 0.025	-0.258 ± 0.045	-0.276 ± 0.048	-0.271 ± 0.042	-0.406 ± 0.047	-0.398 ± 0.045	-0.409 ± 0.040	-0.408 ± 0.039
52	0.048 ± 0.005	0.049 ± 0.009	0.050 ± 0.012	0.052 ± 0.011	-0.257 ± 0.024	-0.261 ± 0.045	-0.279 ± 0.049	-0.273 ± 0.042	-0.408 ± 0.049	-0.400 ± 0.047	-0.411 ± 0.041	-0.410 ± 0.040
53	0.048 ± 0.005	0.050 ± 0.009	0.050 ± 0.012	0.053 ± 0.011	-0.259 ± 0.024	-0.264 ± 0.046	-0.281 ± 0.049	-0.276 ± 0.042	-0.409 ± 0.051	-0.403 ± 0.049	-0.413 ± 0.042	-0.413 ± 0.041
54	0.049 ± 0.005	0.051 ± 0.009	0.051 ± 0.012	0.053 ± 0.011	-0.261 ± 0.023	-0.267 ± 0.046	-0.284 ± 0.050	-0.278 ± 0.042	-0.411 ± 0.053	-0.406 ± 0.050	-0.415 ± 0.043	-0.416 ± 0.042
55	0.050 ± 0.004	0.052 ± 0.008	0.052 ± 0.012	0.054 ± 0.011	-0.263 ± 0.023	-0.269 ± 0.046	-0.286 ± 0.051	-0.280 ± 0.043	-0.414 ± 0.055	-0.409 ± 0.052	-0.418 ± 0.044	-0.419 ± 0.042
56	0.051 ± 0.004	0.052 ± 0.008	0.052 ± 0.012	0.054 ± 0.011	-0.265 ± 0.023	-0.271 ± 0.047	-0.288 ± 0.052	-0.281 ± 0.043	-0.416 ± 0.057	-0.413 ± 0.054	-0.421 ± 0.046	-0.422 ± 0.043
57	0.051 ± 0.004	0.053 ± 0.008	0.053 ± 0.012	0.055 ± 0.011	-0.267 ± 0.022	-0.273 ± 0.047	-0.290 ± 0.053	-0.283 ± 0.043	-0.419 ± 0.059	-0.417 ± 0.055	-0.425 ± 0.047	-0.426 ± 0.044
58	0.052 ± 0.004	0.053 ± 0.008	0.053 ± 0.012	0.055 ± 0.012	-0.268 ± 0.022	-0.275 ± 0.048	-0.292 ± 0.054	-0.284 ± 0.044	-0.422 ± 0.060	-0.421 ± 0.057	-0.429 ± 0.048	-0.430 ± 0.045
59	0.052 ±	0.054 ±	0.054 ±	0.055 ±	-0.270 ±	-0.276 ±	-0.293 ±	-0.285 ±	-0.426 ±	-0.426 ±	-0.434 ±	-0.435 ±

	0.003	0.008	0.012	0.012	0.023	0.048	0.055	0.045	0.062	0.058	0.049	0.046
60	0.053 ± 0.003	0.054 ± 0.008	0.054 ± 0.012	0.056 ± 0.012	-0.271 ± 0.023	-0.278 ± 0.049	-0.294 ± 0.056	-0.286 ± 0.045	-0.430 ± 0.063	-0.431 ± 0.059	-0.439 ± 0.050	-0.440 ± 0.047
61	0.053 ± 0.003	0.054 ± 0.008	0.054 ± 0.012	0.056 ± 0.012	-0.271 ± 0.024	-0.278 ± 0.050	-0.295 ± 0.057	-0.286 ± 0.046	-0.434 ± 0.065	-0.436 ± 0.061	-0.444 ± 0.051	-0.446 ± 0.048
62	0.053 ± 0.003	0.055 ± 0.008	0.055 ± 0.012	0.056 ± 0.012	-0.272 ± 0.025	-0.279 ± 0.051	-0.296 ± 0.058	-0.286 ± 0.047	-0.439 ± 0.066	-0.442 ± 0.062	-0.450 ± 0.052	-0.451 ± 0.049
63	0.054 ± 0.003	0.055 ± 0.008	0.055 ± 0.012	0.056 ± 0.012	-0.272 ± 0.026	-0.279 ± 0.052	-0.296 ± 0.059	-0.286 ± 0.048	-0.444 ± 0.067	-0.448 ± 0.062	-0.456 ± 0.053	-0.458 ± 0.050
64	0.054 ± 0.003	0.055 ± 0.009	0.055 ± 0.013	0.056 ± 0.012	-0.272 ± 0.027	-0.279 ± 0.054	-0.296 ± 0.061	-0.286 ± 0.049	-0.449 ± 0.069	-0.455 ± 0.063	-0.463 ± 0.054	-0.464 ± 0.051
65	0.054 ± 0.003	0.055 ± 0.009	0.055 ± 0.013	0.056 ± 0.012	-0.271 ± 0.028	-0.278 ± 0.055	-0.296 ± 0.062	-0.285 ± 0.051	-0.455 ± 0.070	-0.461 ± 0.064	-0.470 ± 0.055	-0.471 ± 0.051
66	0.054 ± 0.004	0.055 ± 0.009	0.055 ± 0.013	0.056 ± 0.013	-0.270 ± 0.029	-0.277 ± 0.056	-0.295 ± 0.063	-0.284 ± 0.052	-0.461 ± 0.071	-0.469 ± 0.065	-0.477 ± 0.056	-0.479 ± 0.052
67	0.054 ± 0.004	0.055 ± 0.009	0.055 ± 0.013	0.056 ± 0.013	-0.269 ± 0.031	-0.276 ± 0.058	-0.294 ± 0.064	-0.283 ± 0.053	-0.468 ± 0.072	-0.476 ± 0.065	-0.485 ± 0.057	-0.486 ± 0.053
68	0.054 ± 0.004	0.055 ± 0.009	0.055 ± 0.013	0.056 ± 0.013	-0.268 ± 0.032	-0.274 ± 0.059	-0.293 ± 0.066	-0.282 ± 0.055	-0.475 ± 0.073	-0.484 ± 0.066	-0.494 ± 0.058	-0.494 ± 0.055
69	0.053 ± 0.004	0.055 ± 0.010	0.055 ± 0.014	0.055 ± 0.013	-0.266 ± 0.034	-0.272 ± 0.061	-0.291 ± 0.067	-0.280 ± 0.056	-0.483 ± 0.074	-0.492 ± 0.066	-0.503 ± 0.059	-0.503 ± 0.056
70	0.053 ± 0.004	0.054 ± 0.010	0.055 ± 0.014	0.055 ± 0.014	-0.264 ± 0.035	-0.270 ± 0.063	-0.289 ± 0.068	-0.277 ± 0.058	-0.491 ± 0.075	-0.501 ± 0.067	-0.512 ± 0.061	-0.512 ± 0.057
71	0.053 ± 0.004	0.054 ± 0.010	0.054 ± 0.014	0.055 ± 0.014	-0.262 ± 0.037	-0.267 ± 0.064	-0.287 ± 0.070	-0.275 ± 0.059	-0.499 ± 0.076	-0.509 ± 0.068	-0.522 ± 0.062	-0.521 ± 0.058
72	0.052 ± 0.005	0.053 ± 0.011	0.054 ± 0.015	0.054 ± 0.014	-0.259 ± 0.039	-0.264 ± 0.066	-0.284 ± 0.071	-0.272 ± 0.061	-0.508 ± 0.077	-0.518 ± 0.069	-0.532 ± 0.064	-0.531 ± 0.060
73	0.052 ± 0.005	0.053 ± 0.011	0.054 ± 0.015	0.054 ± 0.014	-0.256 ± 0.040	-0.260 ± 0.068	-0.281 ± 0.072	-0.269 ± 0.063	-0.518 ± 0.078	-0.528 ± 0.070	-0.542 ± 0.066	-0.540 ± 0.062
74	0.051 ± 0.005	0.052 ± 0.011	0.053 ± 0.015	0.053 ± 0.015	-0.253 ± 0.042	-0.256 ± 0.069	-0.278 ± 0.074	-0.265 ± 0.064	-0.527 ± 0.079	-0.537 ± 0.071	-0.553 ± 0.068	-0.551 ± 0.064
75	0.051 ± 0.005	0.052 ± 0.012	0.052 ± 0.016	0.052 ± 0.015	-0.249 ± 0.044	-0.252 ± 0.071	-0.274 ± 0.075	-0.262 ± 0.066	-0.537 ± 0.080	-0.547 ± 0.072	-0.564 ± 0.070	-0.561 ± 0.066
76	0.050 ± 0.006	0.051 ± 0.012	0.052 ± 0.016	0.052 ± 0.015	-0.245 ± 0.045	-0.248 ± 0.073	-0.270 ± 0.076	-0.258 ± 0.068	-0.548 ± 0.082	-0.557 ± 0.074	-0.575 ± 0.072	-0.572 ± 0.068
77	0.049 ± 0.006	0.050 ± 0.013	0.051 ± 0.016	0.051 ± 0.016	-0.241 ± 0.047	-0.243 ± 0.075	-0.266 ± 0.077	-0.254 ± 0.069	-0.559 ± 0.083	-0.567 ± 0.076	-0.587 ± 0.075	-0.583 ± 0.070
78	0.048 ± 0.006	0.049 ± 0.013	0.051 ± 0.017	0.050 ± 0.016	-0.237 ± 0.048	-0.238 ± 0.076	-0.262 ± 0.079	-0.249 ± 0.071	-0.570 ± 0.085	-0.578 ± 0.078	-0.599 ± 0.077	-0.594 ± 0.073
79	0.048 ±	0.049 ±	0.050 ±	0.049 ±	-0.232 ±	-0.232 ±	-0.257 ±	-0.244 ±	-0.581 ±	-0.589 ±	-0.612 ±	-0.606 ±

	0.006	0.014	0.017	0.017	0.050	0.078	0.080	0.073	0.087	0.081	0.080	0.075
80	0.047 ± 0.006	0.048 ± 0.014	0.049 ± 0.017	0.048 ± 0.017	-0.227 ± 0.052	-0.227 ± 0.080	-0.252 ± 0.081	-0.239 ± 0.075	-0.593 ± 0.089	-0.599 ± 0.083	-0.625 ± 0.083	-0.617 ± 0.078
81	0.046 ± 0.007	0.047 ± 0.015	0.048 ± 0.018	0.047 ± 0.017	-0.221 ± 0.053	-0.221 ± 0.081	-0.246 ± 0.082	-0.234 ± 0.076	-0.605 ± 0.091	-0.610 ± 0.087	-0.637 ± 0.086	-0.629 ± 0.081
82	0.045 ± 0.007	0.046 ± 0.015	0.047 ± 0.018	0.046 ± 0.018	-0.216 ± 0.055	-0.214 ± 0.083	-0.241 ± 0.083	-0.228 ± 0.078	-0.618 ± 0.094	-0.621 ± 0.090	-0.651 ± 0.090	-0.641 ± 0.085
83	0.044 ± 0.007	0.045 ± 0.016	0.046 ± 0.019	0.045 ± 0.018	-0.210 ± 0.056	-0.208 ± 0.084	-0.235 ± 0.084	-0.223 ± 0.080	-0.631 ± 0.097	-0.633 ± 0.095	-0.664 ± 0.094	-0.654 ± 0.088
84	0.043 ± 0.007	0.044 ± 0.016	0.045 ± 0.019	0.044 ± 0.019	-0.204 ± 0.058	-0.201 ± 0.086	-0.229 ± 0.086	-0.217 ± 0.082	-0.644 ± 0.099	-0.644 ± 0.099	-0.678 ± 0.098	-0.666 ± 0.092
85	0.041 ± 0.007	0.042 ± 0.017	0.044 ± 0.020	0.043 ± 0.019	-0.198 ± 0.059	-0.194 ± 0.087	-0.222 ± 0.087	-0.211 ± 0.084	-0.657 ± 0.103	-0.656 ± 0.104	-0.691 ± 0.102	-0.679 ± 0.096
86	0.040 ± 0.007	0.041 ± 0.017	0.043 ± 0.020	0.042 ± 0.020	-0.191 ± 0.061	-0.188 ± 0.089	-0.216 ± 0.088	-0.205 ± 0.086	-0.671 ± 0.106	-0.667 ± 0.109	-0.705 ± 0.107	-0.692 ± 0.100
87	0.039 ± 0.008	0.040 ± 0.018	0.042 ± 0.021	0.041 ± 0.020	-0.185 ± 0.062	-0.181 ± 0.091	-0.210 ± 0.089	-0.199 ± 0.088	-0.685 ± 0.109	-0.679 ± 0.115	-0.719 ± 0.111	-0.704 ± 0.104
88	0.038 ± 0.008	0.039 ± 0.018	0.041 ± 0.021	0.040 ± 0.021	-0.179 ± 0.064	-0.174 ± 0.092	-0.204 ± 0.091	-0.193 ± 0.089	-0.699 ± 0.113	-0.690 ± 0.120	-0.733 ± 0.116	-0.717 ± 0.109
89	0.037 ± 0.008	0.038 ± 0.019	0.040 ± 0.022	0.038 ± 0.022	-0.173 ± 0.065	-0.167 ± 0.094	-0.197 ± 0.092	-0.187 ± 0.091	-0.713 ± 0.116	-0.702 ± 0.126	-0.747 ± 0.120	-0.730 ± 0.113
90	0.036 ± 0.008	0.037 ± 0.019	0.039 ± 0.022	0.037 ± 0.022	-0.166 ± 0.067	-0.160 ± 0.096	-0.191 ± 0.094	-0.181 ± 0.093	-0.727 ± 0.120	-0.713 ± 0.131	-0.760 ± 0.125	-0.742 ± 0.118
91	0.034 ± 0.008	0.035 ± 0.020	0.038 ± 0.023	0.036 ± 0.023	-0.160 ± 0.069	-0.153 ± 0.098	-0.185 ± 0.095	-0.175 ± 0.095	-0.741 ± 0.124	-0.725 ± 0.137	-0.774 ± 0.130	-0.755 ± 0.122
92	0.033 ± 0.009	0.034 ± 0.021	0.037 ± 0.023	0.035 ± 0.023	-0.154 ± 0.070	-0.146 ± 0.099	-0.179 ± 0.096	-0.169 ± 0.097	-0.755 ± 0.127	-0.736 ± 0.143	-0.788 ± 0.134	-0.768 ± 0.127
93	0.032 ± 0.009	0.033 ± 0.021	0.036 ± 0.024	0.034 ± 0.024	-0.148 ± 0.072	-0.139 ± 0.101	-0.173 ± 0.098	-0.163 ± 0.099	-0.769 ± 0.131	-0.748 ± 0.149	-0.802 ± 0.139	-0.780 ± 0.132
94	0.031 ± 0.009	0.032 ± 0.022	0.035 ± 0.024	0.033 ± 0.024	-0.141 ± 0.074	-0.132 ± 0.103	-0.166 ± 0.099	-0.157 ± 0.101	-0.783 ± 0.134	-0.759 ± 0.155	-0.815 ± 0.144	-0.793 ± 0.136
95	0.030 ± 0.009	0.031 ± 0.022	0.034 ± 0.025	0.032 ± 0.025	-0.135 ± 0.075	-0.125 ± 0.105	-0.160 ± 0.101	-0.151 ± 0.103	-0.798 ± 0.138	-0.771 ± 0.162	-0.829 ± 0.149	-0.806 ± 0.141
96	0.029 ± 0.010	0.030 ± 0.023	0.033 ± 0.025	0.031 ± 0.025	-0.129 ± 0.077	-0.118 ± 0.106	-0.154 ± 0.102	-0.145 ± 0.105	-0.812 ± 0.142	-0.783 ± 0.168	-0.843 ± 0.154	-0.819 ± 0.146
97	0.028 ± 0.010	0.029 ± 0.023	0.032 ± 0.026	0.030 ± 0.026	-0.123 ± 0.079	-0.111 ± 0.108	-0.148 ± 0.103	-0.139 ± 0.107	-0.826 ± 0.145	-0.794 ± 0.174	-0.857 ± 0.159	-0.831 ± 0.151
98	0.026 ± 0.010	0.027 ± 0.024	0.031 ± 0.026	0.028 ± 0.026	-0.117 ± 0.081	-0.105 ± 0.110	-0.141 ± 0.105	-0.133 ± 0.109	-0.841 ± 0.149	-0.806 ± 0.180	-0.870 ± 0.164	-0.844 ± 0.156
99	0.025 ±	0.026 ±	0.030 ±	0.027 ±	-0.110 ±	-0.098 ±	-0.135 ±	-0.127 ±	-0.855 ±	-0.817 ±	-0.884 ±	-0.857 ±

	0.010	0.025	0.027	0.027	0.083	0.112	0.106	0.111	0.153	0.187	0.169	0.160
100	0.024 ± 0.011	0.025 ± 0.025	0.030 ± 0.027	0.026 ± 0.028	-0.104 ± 0.084	-0.091 ± 0.113	-0.129 ± 0.108	-0.121 ± 0.113	-0.870 ± 0.156	-0.829 ± 0.193	-0.898 ± 0.174	-0.869 ± 0.165

Tab. S4 Full normalized knee joint reaction force data during squatting under four knee protector conditions (group mean ± SD, n = 5).

Percent cycle (%)	Fx/BW Pro.off	Fx/BW Pro.a	Fx/BW Pro.b	Fx/BW Pro.c	Fy/BW Pro.off	Fy/BW Pro.a	Fy/BW Pro.b	Fy/BW Pro.c	Fz/BW Pro.off	Fz/BW Pro.a	Fz/BW Pro.b	Fz/BW Pro.c
0	0.009 ± 0.002	0.009 ± 0.006	0.007 ± 0.006	0.008 ± 0.005	-0.031 ± 0.012	-0.032 ± 0.016	-0.038 ± 0.019	-0.033 ± 0.021	-0.263 ± 0.050	-0.233 ± 0.030	-0.241 ± 0.026	-0.255 ± 0.055
1	0.009 ± 0.001	0.010 ± 0.006	0.007 ± 0.005	0.008 ± 0.005	-0.032 ± 0.013	-0.034 ± 0.017	-0.040 ± 0.021	-0.034 ± 0.021	-0.263 ± 0.048	-0.235 ± 0.029	-0.243 ± 0.026	-0.256 ± 0.055
2	0.009 ± 0.001	0.010 ± 0.005	0.007 ± 0.005	0.008 ± 0.005	-0.034 ± 0.014	-0.036 ± 0.018	-0.042 ± 0.022	-0.036 ± 0.021	-0.263 ± 0.047	-0.236 ± 0.029	-0.244 ± 0.026	-0.257 ± 0.054
3	0.009 ± 0.001	0.010 ± 0.005	0.007 ± 0.005	0.008 ± 0.005	-0.035 ± 0.015	-0.038 ± 0.019	-0.044 ± 0.023	-0.037 ± 0.021	-0.263 ± 0.045	-0.238 ± 0.030	-0.245 ± 0.026	-0.258 ± 0.054
4	0.009 ± 0.001	0.010 ± 0.005	0.008 ± 0.005	0.008 ± 0.005	-0.037 ± 0.016	-0.041 ± 0.021	-0.045 ± 0.025	-0.038 ± 0.021	-0.263 ± 0.044	-0.239 ± 0.030	-0.246 ± 0.026	-0.259 ± 0.054
5	0.009 ± 0.001	0.010 ± 0.005	0.008 ± 0.005	0.008 ± 0.005	-0.038 ± 0.017	-0.043 ± 0.023	-0.047 ± 0.026	-0.039 ± 0.021	-0.263 ± 0.043	-0.241 ± 0.030	-0.248 ± 0.026	-0.260 ± 0.053
6	0.009 ± 0.002	0.010 ± 0.005	0.008 ± 0.005	0.008 ± 0.005	-0.039 ± 0.017	-0.045 ± 0.024	-0.049 ± 0.028	-0.040 ± 0.021	-0.264 ± 0.041	-0.242 ± 0.031	-0.249 ± 0.026	-0.261 ± 0.053
7	0.009 ± 0.002	0.010 ± 0.005	0.008 ± 0.005	0.008 ± 0.005	-0.041 ± 0.018	-0.047 ± 0.026	-0.050 ± 0.029	-0.041 ± 0.021	-0.264 ± 0.040	-0.244 ± 0.032	-0.250 ± 0.026	-0.262 ± 0.053
8	0.009 ± 0.002	0.010 ± 0.004	0.008 ± 0.005	0.008 ± 0.005	-0.043 ± 0.019	-0.049 ± 0.028	-0.052 ± 0.030	-0.043 ± 0.021	-0.264 ± 0.039	-0.245 ± 0.032	-0.251 ± 0.026	-0.263 ± 0.053
9	0.009 ± 0.002	0.010 ± 0.004	0.008 ± 0.004	0.008 ± 0.005	-0.044 ± 0.020	-0.052 ± 0.030	-0.054 ± 0.032	-0.044 ± 0.021	-0.264 ± 0.038	-0.247 ± 0.033	-0.253 ± 0.027	-0.264 ± 0.052
10	0.009 ± 0.002	0.010 ± 0.004	0.008 ± 0.004	0.008 ± 0.005	-0.046 ± 0.021	-0.054 ± 0.032	-0.056 ± 0.033	-0.045 ± 0.021	-0.265 ± 0.036	-0.248 ± 0.035	-0.254 ± 0.027	-0.265 ± 0.052
11	0.009 ± 0.002	0.010 ± 0.004	0.008 ± 0.004	0.008 ± 0.005	-0.048 ± 0.022	-0.056 ± 0.034	-0.058 ± 0.035	-0.046 ± 0.021	-0.265 ± 0.035	-0.250 ± 0.036	-0.256 ± 0.028	-0.266 ± 0.052
12	0.010 ± 0.002	0.010 ± 0.004	0.008 ± 0.004	0.008 ± 0.005	-0.050 ± 0.022	-0.059 ± 0.037	-0.060 ± 0.036	-0.048 ± 0.021	-0.266 ± 0.034	-0.252 ± 0.037	-0.257 ± 0.028	-0.268 ± 0.051
13	0.010 ± 0.003	0.010 ± 0.004	0.009 ± 0.004	0.008 ± 0.005	-0.052 ± 0.023	-0.061 ± 0.039	-0.062 ± 0.038	-0.049 ± 0.021	-0.267 ± 0.034	-0.254 ± 0.038	-0.259 ± 0.029	-0.269 ± 0.051
14	0.010 ± 0.003	0.011 ± 0.003	0.009 ± 0.004	0.009 ± 0.005	-0.054 ± 0.024	-0.064 ± 0.041	-0.065 ± 0.040	-0.051 ± 0.021	-0.268 ± 0.033	-0.256 ± 0.040	-0.261 ± 0.030	-0.270 ± 0.050
15	0.010	0.011	0.009	0.009	-0.057	-0.067	-0.067	-0.052	-0.269	-0.258	-0.263	-0.271

	± 0.003	± 0.003	± 0.004	± 0.005	± 0.025	± 0.044	± 0.042	± 0.021	± 0.032	± 0.041	± 0.031	± 0.050
16	0.010 ± 0.003	0.011 ± 0.003	0.009 ± 0.004	0.009 ± 0.005	-0.059 ± 0.026	-0.070 ± 0.046	-0.070 ± 0.044	-0.054 ± 0.021	-0.271 ± 0.031	-0.261 ± 0.043	-0.265 ± 0.032	-0.273 ± 0.049
17	0.010 ± 0.003	0.011 ± 0.003	0.009 ± 0.003	0.009 ± 0.006	-0.062 ± 0.028	-0.073 ± 0.049	-0.072 ± 0.046	-0.056 ± 0.021	-0.272 ± 0.031	-0.263 ± 0.045	-0.267 ± 0.033	-0.274 ± 0.049
18	0.010 ± 0.003	0.011 ± 0.003	0.010 ± 0.003	0.009 ± 0.006	-0.065 ± 0.029	-0.077 ± 0.051	-0.075 ± 0.048	-0.058 ± 0.021	-0.274 ± 0.030	-0.266 ± 0.046	-0.270 ± 0.035	-0.276 ± 0.048
19	0.010 ± 0.003	0.012 ± 0.003	0.010 ± 0.003	0.009 ± 0.006	-0.068 ± 0.030	-0.080 ± 0.054	-0.078 ± 0.050	-0.060 ± 0.021	-0.276 ± 0.030	-0.268 ± 0.048	-0.273 ± 0.036	-0.277 ± 0.048
20	0.011 ± 0.003	0.012 ± 0.003	0.010 ± 0.003	0.009 ± 0.006	-0.072 ± 0.032	-0.084 ± 0.057	-0.082 ± 0.052	-0.062 ± 0.021	-0.278 ± 0.030	-0.271 ± 0.050	-0.275 ± 0.037	-0.279 ± 0.047
21	0.011 ± 0.004	0.012 ± 0.003	0.011 ± 0.003	0.009 ± 0.006	-0.075 ± 0.034	-0.088 ± 0.060	-0.085 ± 0.055	-0.065 ± 0.021	-0.280 ± 0.030	-0.275 ± 0.052	-0.278 ± 0.039	-0.281 ± 0.046
22	0.011 ± 0.004	0.013 ± 0.003	0.011 ± 0.003	0.010 ± 0.006	-0.079 ± 0.036	-0.092 ± 0.063	-0.089 ± 0.057	-0.067 ± 0.021	-0.283 ± 0.030	-0.278 ± 0.053	-0.282 ± 0.041	-0.284 ± 0.045
23	0.012 ± 0.004	0.013 ± 0.004	0.012 ± 0.003	0.010 ± 0.006	-0.084 ± 0.038	-0.097 ± 0.066	-0.093 ± 0.060	-0.070 ± 0.021	-0.285 ± 0.030	-0.281 ± 0.055	-0.285 ± 0.042	-0.286 ± 0.044
24	0.012 ± 0.004	0.014 ± 0.004	0.012 ± 0.004	0.010 ± 0.006	-0.088 ± 0.040	-0.101 ± 0.069	-0.097 ± 0.062	-0.073 ± 0.021	-0.288 ± 0.030	-0.285 ± 0.057	-0.289 ± 0.044	-0.288 ± 0.043
25	0.012 ± 0.004	0.014 ± 0.005	0.013 ± 0.004	0.010 ± 0.007	-0.093 ± 0.043	-0.106 ± 0.072	-0.102 ± 0.065	-0.077 ± 0.021	-0.291 ± 0.030	-0.289 ± 0.059	-0.292 ± 0.046	-0.291 ± 0.043
26	0.013 ± 0.005	0.015 ± 0.005	0.013 ± 0.004	0.011 ± 0.007	-0.098 ± 0.045	-0.111 ± 0.075	-0.107 ± 0.068	-0.080 ± 0.021	-0.295 ± 0.031	-0.294 ± 0.061	-0.297 ± 0.048	-0.294 ± 0.042
27	0.013 ± 0.005	0.015 ± 0.006	0.014 ± 0.005	0.011 ± 0.007	-0.103 ± 0.048	-0.117 ± 0.078	-0.111 ± 0.070	-0.084 ± 0.022	-0.298 ± 0.032	-0.298 ± 0.063	-0.301 ± 0.050	-0.297 ± 0.041
28	0.014 ± 0.005	0.016 ± 0.006	0.015 ± 0.005	0.012 ± 0.007	-0.109 ± 0.052	-0.122 ± 0.080	-0.117 ± 0.073	-0.088 ± 0.022	-0.302 ± 0.033	-0.303 ± 0.065	-0.305 ± 0.052	-0.301 ± 0.040
29	0.015 ± 0.005	0.017 ± 0.007	0.015 ± 0.006	0.012 ± 0.008	-0.115 ± 0.055	-0.128 ± 0.083	-0.122 ± 0.076	-0.092 ± 0.022	-0.306 ± 0.033	-0.308 ± 0.066	-0.310 ± 0.054	-0.304 ± 0.039
30	0.016 ± 0.006	0.018 ± 0.008	0.016 ± 0.007	0.013 ± 0.008	-0.122 ± 0.059	-0.135 ± 0.086	-0.128 ± 0.079	-0.097 ± 0.022	-0.311 ± 0.034	-0.314 ± 0.068	-0.315 ± 0.056	-0.308 ± 0.038
31	0.016 ± 0.006	0.019 ± 0.008	0.017 ± 0.007	0.013 ± 0.008	-0.129 ± 0.062	-0.141 ± 0.089	-0.134 ± 0.082	-0.102 ± 0.023	-0.316 ± 0.035	-0.319 ± 0.069	-0.320 ± 0.058	-0.313 ± 0.038
32	0.017 ± 0.007	0.020 ± 0.009	0.018 ± 0.008	0.014 ± 0.009	-0.136 ± 0.066	-0.148 ± 0.091	-0.141 ± 0.084	-0.107 ± 0.023	-0.321 ± 0.036	-0.326 ± 0.070	-0.326 ± 0.060	-0.317 ± 0.037
33	0.018 ± 0.008	0.021 ± 0.009	0.019 ± 0.009	0.015 ± 0.009	-0.143 ± 0.070	-0.155 ± 0.094	-0.147 ± 0.087	-0.112 ± 0.024	-0.327 ± 0.037	-0.332 ± 0.071	-0.332 ± 0.062	-0.322 ± 0.037
34	0.019 ± 0.008	0.022 ± 0.010	0.020 ± 0.009	0.016 ± 0.010	-0.151 ± 0.075	-0.163 ± 0.096	-0.154 ± 0.089	-0.118 ± 0.025	-0.333 ± 0.038	-0.339 ± 0.072	-0.338 ± 0.064	-0.328 ± 0.037
35	0.021	0.023	0.021	0.017	-0.159	-0.171	-0.162	-0.124	-0.340	-0.346	-0.344	-0.333

	± 0.009	± 0.011	± 0.010	± 0.010	± 0.079	± 0.098	± 0.092	± 0.026	± 0.039	± 0.072	± 0.065	± 0.037
36	0.022 ± 0.010	0.025 ± 0.011	0.023 ± 0.011	0.018 ± 0.011	-0.168 ± 0.083	-0.179 ± 0.100	-0.169 ± 0.094	-0.131 ± 0.027	-0.347 ± 0.040	-0.354 ± 0.072	-0.351 ± 0.067	-0.339 ± 0.037
37	0.023 ± 0.011	0.026 ± 0.012	0.024 ± 0.012	0.019 ± 0.011	-0.177 ± 0.088	-0.188 ± 0.102	-0.177 ± 0.096	-0.137 ± 0.028	-0.355 ± 0.041	-0.362 ± 0.072	-0.358 ± 0.068	-0.346 ± 0.038
38	0.025 ± 0.011	0.027 ± 0.012	0.026 ± 0.012	0.020 ± 0.012	-0.186 ± 0.092	-0.196 ± 0.104	-0.185 ± 0.098	-0.145 ± 0.030	-0.364 ± 0.043	-0.371 ± 0.072	-0.365 ± 0.068	-0.352 ± 0.039
39	0.027 ± 0.012	0.029 ± 0.013	0.027 ± 0.013	0.021 ± 0.013	-0.196 ± 0.096	-0.206 ± 0.105	-0.194 ± 0.100	-0.152 ± 0.032	-0.373 ± 0.045	-0.380 ± 0.071	-0.373 ± 0.069	-0.359 ± 0.040
40	0.028 ± 0.013	0.031 ± 0.013	0.029 ± 0.014	0.022 ± 0.014	-0.205 ± 0.101	-0.215 ± 0.106	-0.203 ± 0.102	-0.160 ± 0.034	-0.382 ± 0.047	-0.389 ± 0.071	-0.381 ± 0.069	-0.367 ± 0.042
41	0.030 ± 0.014	0.033 ± 0.014	0.030 ± 0.014	0.024 ± 0.014	-0.216 ± 0.105	-0.225 ± 0.107	-0.212 ± 0.103	-0.168 ± 0.037	-0.392 ± 0.050	-0.399 ± 0.070	-0.389 ± 0.069	-0.375 ± 0.044
42	0.032 ± 0.015	0.035 ± 0.014	0.032 ± 0.015	0.025 ± 0.015	-0.226 ± 0.108	-0.236 ± 0.107	-0.221 ± 0.104	-0.177 ± 0.039	-0.403 ± 0.054	-0.409 ± 0.069	-0.398 ± 0.068	-0.383 ± 0.046
43	0.034 ± 0.016	0.037 ± 0.014	0.034 ± 0.015	0.027 ± 0.016	-0.237 ± 0.112	-0.246 ± 0.108	-0.231 ± 0.105	-0.186 ± 0.042	-0.413 ± 0.059	-0.419 ± 0.068	-0.407 ± 0.068	-0.391 ± 0.049
44	0.036 ± 0.017	0.039 ± 0.014	0.036 ± 0.016	0.029 ± 0.017	-0.248 ± 0.115	-0.257 ± 0.108	-0.242 ± 0.106	-0.195 ± 0.046	-0.424 ± 0.064	-0.430 ± 0.067	-0.416 ± 0.067	-0.400 ± 0.052
45	0.038 ± 0.017	0.041 ± 0.015	0.038 ± 0.016	0.031 ± 0.018	-0.259 ± 0.118	-0.269 ± 0.108	-0.252 ± 0.106	-0.205 ± 0.049	-0.436 ± 0.070	-0.441 ± 0.067	-0.425 ± 0.066	-0.409 ± 0.055
46	0.040 ± 0.018	0.043 ± 0.015	0.041 ± 0.016	0.033 ± 0.019	-0.271 ± 0.120	-0.280 ± 0.107	-0.263 ± 0.106	-0.215 ± 0.053	-0.447 ± 0.076	-0.452 ± 0.067	-0.435 ± 0.064	-0.419 ± 0.059
47	0.042 ± 0.018	0.046 ± 0.015	0.043 ± 0.017	0.035 ± 0.021	-0.282 ± 0.122	-0.292 ± 0.107	-0.274 ± 0.106	-0.225 ± 0.057	-0.459 ± 0.082	-0.464 ± 0.067	-0.445 ± 0.063	-0.428 ± 0.063
48	0.045 ± 0.019	0.048 ± 0.015	0.045 ± 0.017	0.037 ± 0.022	-0.294 ± 0.123	-0.305 ± 0.106	-0.285 ± 0.106	-0.235 ± 0.061	-0.470 ± 0.089	-0.475 ± 0.068	-0.455 ± 0.062	-0.438 ± 0.067
49	0.047 ± 0.019	0.051 ± 0.015	0.048 ± 0.017	0.039 ± 0.023	-0.306 ± 0.124	-0.317 ± 0.105	-0.297 ± 0.105	-0.246 ± 0.065	-0.482 ± 0.095	-0.487 ± 0.069	-0.466 ± 0.061	-0.448 ± 0.071
50	0.049 ± 0.019	0.053 ± 0.015	0.050 ± 0.018	0.041 ± 0.024	-0.318 ± 0.125	-0.330 ± 0.105	-0.309 ± 0.105	-0.257 ± 0.070	-0.494 ± 0.102	-0.499 ± 0.071	-0.476 ± 0.061	-0.458 ± 0.076
51	0.052 ± 0.019	0.056 ± 0.016	0.053 ± 0.018	0.044 ± 0.026	-0.331 ± 0.125	-0.343 ± 0.104	-0.321 ± 0.104	-0.269 ± 0.074	-0.505 ± 0.109	-0.511 ± 0.074	-0.487 ± 0.061	-0.468 ± 0.081
52	0.054 ± 0.020	0.059 ± 0.016	0.055 ± 0.018	0.046 ± 0.027	-0.343 ± 0.125	-0.356 ± 0.103	-0.334 ± 0.103	-0.280 ± 0.079	-0.517 ± 0.115	-0.522 ± 0.076	-0.498 ± 0.061	-0.479 ± 0.086
53	0.057 ± 0.020	0.061 ± 0.016	0.058 ± 0.019	0.049 ± 0.028	-0.356 ± 0.124	-0.369 ± 0.102	-0.346 ± 0.103	-0.292 ± 0.084	-0.528 ± 0.121	-0.534 ± 0.080	-0.508 ± 0.062	-0.489 ± 0.091
54	0.059 ± 0.020	0.064 ± 0.017	0.061 ± 0.019	0.051 ± 0.030	-0.369 ± 0.124	-0.382 ± 0.101	-0.359 ± 0.102	-0.304 ± 0.089	-0.539 ± 0.127	-0.545 ± 0.083	-0.519 ± 0.064	-0.499 ± 0.096
55	0.062	0.067	0.063	0.054	-0.382	-0.396	-0.372	-0.316	-0.550	-0.557	-0.529	-0.509

	± 0.020	± 0.017	± 0.020	± 0.031	± 0.123	± 0.101	± 0.102	± 0.094	± 0.133	± 0.087	± 0.066	± 0.101
56	0.065 ± 0.020	0.070 ± 0.018	0.066 ± 0.021	0.056 ± 0.033	-0.395 ± 0.122	-0.409 ± 0.100	-0.385 ± 0.102	-0.328 ± 0.099	-0.561 ± 0.139	-0.568 ± 0.090	-0.539 ± 0.068	-0.519 ± 0.106
57	0.067 ± 0.020	0.073 ± 0.018	0.069 ± 0.021	0.059 ± 0.034	-0.408 ± 0.121	-0.423 ± 0.100	-0.398 ± 0.102	-0.340 ± 0.105	-0.571 ± 0.145	-0.578 ± 0.094	-0.550 ± 0.071	-0.529 ± 0.111
58	0.070 ± 0.020	0.075 ± 0.019	0.072 ± 0.022	0.061 ± 0.036	-0.422 ± 0.120	-0.436 ± 0.100	-0.411 ± 0.102	-0.352 ± 0.110	-0.582 ± 0.150	-0.589 ± 0.098	-0.559 ± 0.074	-0.539 ± 0.116
59	0.073 ± 0.020	0.078 ± 0.019	0.074 ± 0.023	0.064 ± 0.037	-0.436 ± 0.119	-0.450 ± 0.100	-0.424 ± 0.103	-0.365 ± 0.115	-0.592 ± 0.156	-0.599 ± 0.101	-0.569 ± 0.077	-0.548 ± 0.121
60	0.076 ± 0.020	0.081 ± 0.020	0.077 ± 0.024	0.067 ± 0.039	-0.449 ± 0.118	-0.463 ± 0.100	-0.437 ± 0.104	-0.377 ± 0.121	-0.602 ± 0.162	-0.609 ± 0.105	-0.579 ± 0.080	-0.558 ± 0.126
61	0.079 ± 0.021	0.084 ± 0.021	0.080 ± 0.025	0.069 ± 0.040	-0.463 ± 0.117	-0.477 ± 0.101	-0.450 ± 0.105	-0.389 ± 0.126	-0.612 ± 0.167	-0.618 ± 0.108	-0.588 ± 0.083	-0.567 ± 0.131
62	0.082 ± 0.021	0.086 ± 0.022	0.082 ± 0.026	0.072 ± 0.042	-0.477 ± 0.116	-0.490 ± 0.102	-0.464 ± 0.107	-0.401 ± 0.132	-0.622 ± 0.173	-0.628 ± 0.112	-0.597 ± 0.086	-0.576 ± 0.135
63	0.084 ± 0.021	0.089 ± 0.023	0.085 ± 0.027	0.074 ± 0.043	-0.491 ± 0.116	-0.503 ± 0.104	-0.477 ± 0.109	-0.414 ± 0.137	-0.631 ± 0.178	-0.637 ± 0.115	-0.605 ± 0.089	-0.585 ± 0.140
64	0.087 ± 0.022	0.092 ± 0.024	0.088 ± 0.028	0.077 ± 0.044	-0.506 ± 0.115	-0.517 ± 0.105	-0.490 ± 0.111	-0.426 ± 0.142	-0.641 ± 0.184	-0.645 ± 0.118	-0.614 ± 0.093	-0.593 ± 0.144
65	0.090 ± 0.022	0.095 ± 0.025	0.090 ± 0.029	0.079 ± 0.046	-0.520 ± 0.116	-0.530 ± 0.108	-0.503 ± 0.114	-0.438 ± 0.148	-0.650 ± 0.190	-0.654 ± 0.122	-0.622 ± 0.096	-0.602 ± 0.148
66	0.093 ± 0.023	0.097 ± 0.026	0.093 ± 0.030	0.082 ± 0.047	-0.534 ± 0.116	-0.543 ± 0.110	-0.517 ± 0.117	-0.450 ± 0.153	-0.660 ± 0.196	-0.662 ± 0.125	-0.630 ± 0.099	-0.610 ± 0.153
67	0.096 ± 0.023	0.100 ± 0.027	0.096 ± 0.032	0.084 ± 0.049	-0.549 ± 0.117	-0.557 ± 0.113	-0.530 ± 0.121	-0.463 ± 0.159	-0.669 ± 0.203	-0.670 ± 0.128	-0.638 ± 0.102	-0.617 ± 0.157
68	0.099 ± 0.024	0.103 ± 0.028	0.098 ± 0.033	0.087 ± 0.050	-0.563 ± 0.118	-0.570 ± 0.116	-0.543 ± 0.125	-0.475 ± 0.164	-0.678 ± 0.209	-0.677 ± 0.131	-0.646 ± 0.105	-0.625 ± 0.161
69	0.102 ± 0.025	0.105 ± 0.029	0.101 ± 0.034	0.089 ± 0.051	-0.577 ± 0.120	-0.583 ± 0.120	-0.556 ± 0.129	-0.487 ± 0.170	-0.687 ± 0.216	-0.685 ± 0.134	-0.653 ± 0.108	-0.632 ± 0.165
70	0.105 ± 0.026	0.108 ± 0.030	0.104 ± 0.035	0.092 ± 0.053	-0.592 ± 0.122	-0.596 ± 0.124	-0.570 ± 0.133	-0.498 ± 0.176	-0.696 ± 0.223	-0.692 ± 0.136	-0.660 ± 0.111	-0.640 ± 0.168
71	0.108 ± 0.027	0.111 ± 0.032	0.106 ± 0.037	0.094 ± 0.054	-0.606 ± 0.125	-0.609 ± 0.128	-0.583 ± 0.138	-0.510 ± 0.181	-0.705 ± 0.230	-0.699 ± 0.139	-0.668 ± 0.114	-0.647 ± 0.172
72	0.112 ± 0.028	0.113 ± 0.033	0.109 ± 0.038	0.097 ± 0.055	-0.620 ± 0.128	-0.622 ± 0.133	-0.596 ± 0.143	-0.522 ± 0.187	-0.714 ± 0.237	-0.705 ± 0.142	-0.675 ± 0.117	-0.653 ± 0.176
73	0.115 ± 0.029	0.116 ± 0.034	0.112 ± 0.039	0.099 ± 0.057	-0.635 ± 0.132	-0.635 ± 0.138	-0.610 ± 0.148	-0.533 ± 0.193	-0.722 ± 0.245	-0.712 ± 0.145	-0.682 ± 0.120	-0.660 ± 0.180
74	0.118 ± 0.031	0.119 ± 0.036	0.115 ± 0.041	0.102 ± 0.058	-0.649 ± 0.137	-0.648 ± 0.144	-0.623 ± 0.154	-0.545 ± 0.198	-0.731 ± 0.253	-0.718 ± 0.147	-0.688 ± 0.123	-0.666 ± 0.183
75	0.121	0.121	0.117	0.104	-0.664	-0.661	-0.636	-0.556	-0.740	-0.724	-0.695	-0.673

	± 0.032	± 0.037	± 0.042	± 0.060	± 0.143	± 0.149	± 0.159	± 0.204	± 0.261	± 0.150	± 0.126	± 0.187
76	0.124 ± 0.034	0.124 ± 0.039	0.120 ± 0.044	0.106 ± 0.061	-0.678 ± 0.149	-0.674 ± 0.155	-0.649 ± 0.165	-0.567 ± 0.210	-0.749 ± 0.269	-0.730 ± 0.152	-0.702 ± 0.129	-0.679 ± 0.190
77	0.127 ± 0.036	0.127 ± 0.041	0.123 ± 0.045	0.108 ± 0.062	-0.693 ± 0.156	-0.686 ± 0.162	-0.663 ± 0.171	-0.578 ± 0.216	-0.757 ± 0.278	-0.735 ± 0.155	-0.708 ± 0.131	-0.684 ± 0.194
78	0.131 ± 0.039	0.129 ± 0.043	0.126 ± 0.047	0.111 ± 0.064	-0.707 ± 0.164	-0.699 ± 0.168	-0.676 ± 0.178	-0.589 ± 0.221	-0.766 ± 0.287	-0.741 ± 0.157	-0.714 ± 0.134	-0.690 ± 0.197
79	0.134 ± 0.041	0.132 ± 0.044	0.129 ± 0.048	0.113 ± 0.065	-0.722 ± 0.173	-0.711 ± 0.175	-0.689 ± 0.184	-0.600 ± 0.227	-0.775 ± 0.297	-0.746 ± 0.159	-0.720 ± 0.137	-0.695 ± 0.200
80	0.137 ± 0.044	0.134 ± 0.046	0.132 ± 0.050	0.115 ± 0.066	-0.737 ± 0.183	-0.724 ± 0.181	-0.702 ± 0.190	-0.610 ± 0.233	-0.783 ± 0.306	-0.751 ± 0.162	-0.727 ± 0.140	-0.701 ± 0.203
81	0.141 ± 0.047	0.137 ± 0.048	0.135 ± 0.052	0.117 ± 0.067	-0.752 ± 0.194	-0.736 ± 0.188	-0.715 ± 0.197	-0.620 ± 0.239	-0.792 ± 0.316	-0.756 ± 0.164	-0.732 ± 0.143	-0.706 ± 0.207
82	0.145 ± 0.050	0.140 ± 0.050	0.138 ± 0.053	0.119 ± 0.069	-0.767 ± 0.206	-0.748 ± 0.194	-0.728 ± 0.204	-0.630 ± 0.244	-0.801 ± 0.326	-0.761 ± 0.166	-0.738 ± 0.145	-0.711 ± 0.210
83	0.148 ± 0.053	0.142 ± 0.052	0.141 ± 0.055	0.121 ± 0.070	-0.782 ± 0.218	-0.759 ± 0.201	-0.740 ± 0.210	-0.640 ± 0.250	-0.809 ± 0.337	-0.765 ± 0.168	-0.744 ± 0.148	-0.715 ± 0.212
84	0.152 ± 0.057	0.144 ± 0.053	0.143 ± 0.057	0.123 ± 0.071	-0.798 ± 0.232	-0.771 ± 0.208	-0.753 ± 0.217	-0.650 ± 0.256	-0.818 ± 0.348	-0.770 ± 0.170	-0.749 ± 0.151	-0.719 ± 0.215
85	0.156 ± 0.061	0.147 ± 0.055	0.146 ± 0.058	0.125 ± 0.073	-0.813 ± 0.246	-0.782 ± 0.214	-0.765 ± 0.224	-0.659 ± 0.261	-0.826 ± 0.358	-0.774 ± 0.172	-0.754 ± 0.153	-0.724 ± 0.218
86	0.160 ± 0.066	0.149 ± 0.057	0.149 ± 0.060	0.127 ± 0.074	-0.829 ± 0.261	-0.793 ± 0.220	-0.778 ± 0.230	-0.668 ± 0.267	-0.835 ± 0.369	-0.778 ± 0.173	-0.760 ± 0.156	-0.728 ± 0.220
87	0.164 ± 0.070	0.151 ± 0.059	0.152 ± 0.062	0.129 ± 0.075	-0.845 ± 0.277	-0.804 ± 0.226	-0.790 ± 0.237	-0.676 ± 0.272	-0.843 ± 0.380	-0.782 ± 0.175	-0.765 ± 0.158	-0.731 ± 0.223
88	0.168 ± 0.075	0.154 ± 0.060	0.155 ± 0.064	0.131 ± 0.076	-0.862 ± 0.294	-0.814 ± 0.232	-0.801 ± 0.244	-0.685 ± 0.277	-0.851 ± 0.391	-0.786 ± 0.176	-0.769 ± 0.161	-0.735 ± 0.225
89	0.173 ± 0.080	0.156 ± 0.062	0.158 ± 0.066	0.133 ± 0.077	-0.878 ± 0.312	-0.825 ± 0.237	-0.813 ± 0.250	-0.693 ± 0.282	-0.859 ± 0.401	-0.790 ± 0.178	-0.774 ± 0.163	-0.738 ± 0.227
90	0.177 ± 0.086	0.158 ± 0.063	0.161 ± 0.067	0.134 ± 0.079	-0.895 ± 0.331	-0.835 ± 0.242	-0.825 ± 0.257	-0.701 ± 0.288	-0.867 ± 0.411	-0.793 ± 0.179	-0.778 ± 0.165	-0.742 ± 0.229
91	0.182 ± 0.091	0.160 ± 0.065	0.163 ± 0.069	0.136 ± 0.080	-0.912 ± 0.350	-0.844 ± 0.247	-0.836 ± 0.263	-0.709 ± 0.293	-0.874 ± 0.421	-0.797 ± 0.180	-0.783 ± 0.167	-0.745 ± 0.231
92	0.186 ± 0.097	0.162 ± 0.066	0.166 ± 0.071	0.138 ± 0.081	-0.930 ± 0.371	-0.854 ± 0.252	-0.847 ± 0.270	-0.717 ± 0.298	-0.882 ± 0.430	-0.800 ± 0.181	-0.787 ± 0.170	-0.748 ± 0.233
93	0.191 ± 0.104	0.164 ± 0.067	0.169 ± 0.073	0.139 ± 0.082	-0.947 ± 0.392	-0.863 ± 0.256	-0.858 ± 0.276	-0.724 ± 0.302	-0.889 ± 0.440	-0.803 ± 0.181	-0.791 ± 0.172	-0.751 ± 0.235
94	0.196 ± 0.110	0.166 ± 0.068	0.172 ± 0.075	0.141 ± 0.083	-0.965 ± 0.414	-0.872 ± 0.260	-0.869 ± 0.282	-0.732 ± 0.307	-0.895 ± 0.448	-0.806 ± 0.182	-0.795 ± 0.174	-0.753 ± 0.237
95	0.201	0.168	0.174	0.142	-0.983	-0.881	-0.880	-0.739	-0.902	-0.810	-0.799	-0.756

	± 0.117	± 0.069	± 0.076	± 0.084	± 0.436	± 0.264	± 0.288	± 0.312	± 0.457	± 0.183	± 0.176	± 0.239
96	0.206 ± 0.124	0.170 ± 0.071	0.177 ± 0.078	0.144 ± 0.085	-1.002 ± 0.459	-0.890 ± 0.267	-0.891 ± 0.295	-0.746 ± 0.316	-0.908 ± 0.465	-0.813 ± 0.183	-0.803 ± 0.179	-0.759 ± 0.240
97	0.211 ± 0.131	0.172 ± 0.072	0.180 ± 0.080	0.145 ± 0.086	-1.020 ± 0.483	-0.899 ± 0.271	-0.901 ± 0.301	-0.754 ± 0.321	-0.915 ± 0.473	-0.816 ± 0.184	-0.807 ± 0.181	-0.761 ± 0.242
98	0.216 ± 0.139	0.174 ± 0.073	0.182 ± 0.082	0.147 ± 0.087	-1.039 ± 0.507	-0.908 ± 0.275	-0.912 ± 0.307	-0.761 ± 0.325	-0.921 ± 0.481	-0.819 ± 0.185	-0.811 ± 0.183	-0.764 ± 0.243
99	0.221 ± 0.146	0.175 ± 0.074	0.185 ± 0.084	0.148 ± 0.088	-1.058 ± 0.532	-0.917 ± 0.278	-0.923 ± 0.313	-0.768 ± 0.330	-0.928 ± 0.489	-0.822 ± 0.185	-0.815 ± 0.185	-0.766 ± 0.245
100	0.227 ± 0.154	0.177 ± 0.075	0.188 ± 0.085	0.150 ± 0.089	-1.077 ± 0.557	-0.926 ± 0.282	-0.934 ± 0.320	-0.775 ± 0.334	-0.934 ± 0.497	-0.825 ± 0.186	-0.818 ± 0.188	-0.769 ± 0.246

Tab. S5 Full normalized knee joint reaction force data during STS under four knee protector conditions (group mean ± SD, n = 5).

Percent cycle (%)	Fx/BW Pro.off	Fx/BW Pro.a	Fx/BW Pro.b	Fx/BW Pro.c	Fy/BW Pro.off	Fy/BW Pro.a	Fy/BW Pro.b	Fy/BW Pro.c	Fz/BW Pro.off	Fz/BW Pro.a	Fz/BW Pro.b	Fz/BW Pro.c
0	0.020 ± 0.025	0.010 ± 0.004	0.010 ± 0.006	0.013 ± 0.008	-0.150 ± 0.246	-0.031 ± 0.016	-0.041 ± 0.029	-0.050 ± 0.036	-0.313 ± 0.080	-0.270 ± 0.059	-0.267 ± 0.032	-0.256 ± 0.046
1	0.023 ± 0.030	0.011 ± 0.004	0.011 ± 0.007	0.014 ± 0.009	-0.166 ± 0.279	-0.037 ± 0.019	-0.045 ± 0.035	-0.056 ± 0.044	-0.320 ± 0.095	-0.275 ± 0.046	-0.269 ± 0.029	-0.261 ± 0.042
2	0.026 ± 0.037	0.012 ± 0.005	0.011 ± 0.008	0.015 ± 0.011	-0.184 ± 0.314	-0.043 ± 0.026	-0.050 ± 0.042	-0.064 ± 0.055	-0.328 ± 0.111	-0.281 ± 0.033	-0.271 ± 0.027	-0.267 ± 0.040
3	0.029 ± 0.044	0.013 ± 0.006	0.012 ± 0.010	0.017 ± 0.014	-0.201 ± 0.348	-0.050 ± 0.036	-0.056 ± 0.051	-0.072 ± 0.068	-0.337 ± 0.129	-0.287 ± 0.024	-0.275 ± 0.026	-0.273 ± 0.041
4	0.033 ± 0.053	0.015 ± 0.009	0.013 ± 0.012	0.018 ± 0.016	-0.218 ± 0.383	-0.056 ± 0.046	-0.063 ± 0.062	-0.082 ± 0.083	-0.346 ± 0.149	-0.293 ± 0.024	-0.279 ± 0.029	-0.281 ± 0.047
5	0.037 ± 0.062	0.016 ± 0.011	0.015 ± 0.014	0.020 ± 0.020	-0.235 ± 0.415	-0.064 ± 0.058	-0.070 ± 0.076	-0.093 ± 0.101	-0.355 ± 0.169	-0.299 ± 0.033	-0.284 ± 0.036	-0.290 ± 0.057
6	0.042 ± 0.072	0.017 ± 0.015	0.016 ± 0.017	0.022 ± 0.024	-0.250 ± 0.444	-0.072 ± 0.072	-0.079 ± 0.092	-0.105 ± 0.121	-0.365 ± 0.190	-0.306 ± 0.048	-0.289 ± 0.047	-0.300 ± 0.070
7	0.047 ± 0.083	0.019 ± 0.019	0.018 ± 0.021	0.025 ± 0.029	-0.264 ± 0.470	-0.081 ± 0.089	-0.089 ± 0.111	-0.119 ± 0.143	-0.374 ± 0.212	-0.314 ± 0.066	-0.296 ± 0.061	-0.311 ± 0.086
8	0.052 ± 0.094	0.022 ± 0.023	0.020 ± 0.026	0.028 ± 0.034	-0.277 ± 0.491	-0.092 ± 0.108	-0.101 ± 0.132	-0.134 ± 0.166	-0.384 ± 0.232	-0.323 ± 0.086	-0.304 ± 0.077	-0.323 ± 0.104
9	0.056 ± 0.104	0.024 ± 0.029	0.023 ± 0.031	0.032 ± 0.040	-0.288 ± 0.509	-0.104 ± 0.129	-0.113 ± 0.156	-0.150 ± 0.192	-0.393 ± 0.251	-0.332 ± 0.107	-0.312 ± 0.097	-0.336 ± 0.123
10	0.061 ± 0.113	0.027 ± 0.035	0.026 ± 0.038	0.035 ± 0.047	-0.298 ± 0.523	-0.117 ± 0.153	-0.127 ± 0.182	-0.168 ± 0.219	-0.401 ± 0.267	-0.342 ± 0.128	-0.322 ± 0.118	-0.350 ± 0.143

11	0.065 ± 0.121	0.030 ± 0.042	0.029 ± 0.044	0.040 ± 0.054	-0.307 ± 0.535	-0.131 ± 0.178	-0.142 ± 0.210	-0.187 ± 0.246	-0.409 ± 0.281	-0.352 ± 0.149	-0.332 ± 0.140	-0.364 ± 0.163
12	0.068 ± 0.128	0.034 ± 0.050	0.032 ± 0.052	0.044 ± 0.061	-0.316 ± 0.547	-0.147 ± 0.205	-0.158 ± 0.240	-0.207 ± 0.275	-0.416 ± 0.291	-0.362 ± 0.169	-0.343 ± 0.162	-0.379 ± 0.181
13	0.071 ± 0.133	0.038 ± 0.058	0.036 ± 0.060	0.049 ± 0.069	-0.325 ± 0.558	-0.164 ± 0.234	-0.176 ± 0.273	-0.228 ± 0.303	-0.421 ± 0.297	-0.372 ± 0.188	-0.353 ± 0.184	-0.394 ± 0.199
14	0.074 ± 0.138	0.042 ± 0.066	0.041 ± 0.069	0.054 ± 0.078	-0.336 ± 0.569	-0.181 ± 0.264	-0.194 ± 0.307	-0.250 ± 0.332	-0.427 ± 0.300	-0.382 ± 0.205	-0.364 ± 0.204	-0.410 ± 0.215
15	0.076 ± 0.141	0.047 ± 0.075	0.045 ± 0.079	0.060 ± 0.086	-0.347 ± 0.582	-0.200 ± 0.294	-0.215 ± 0.343	-0.273 ± 0.360	-0.432 ± 0.301	-0.392 ± 0.219	-0.374 ± 0.223	-0.426 ± 0.229
16	0.079 ± 0.145	0.052 ± 0.084	0.051 ± 0.089	0.066 ± 0.095	-0.359 ± 0.594	-0.219 ± 0.324	-0.236 ± 0.381	-0.297 ± 0.386	-0.437 ± 0.299	-0.402 ± 0.232	-0.384 ± 0.240	-0.442 ± 0.241
17	0.082 ± 0.149	0.057 ± 0.094	0.056 ± 0.101	0.072 ± 0.103	-0.372 ± 0.607	-0.239 ± 0.353	-0.259 ± 0.420	-0.321 ± 0.412	-0.443 ± 0.297	-0.413 ± 0.242	-0.394 ± 0.254	-0.458 ± 0.251
18	0.085 ± 0.153	0.063 ± 0.103	0.063 ± 0.114	0.079 ± 0.112	-0.384 ± 0.618	-0.260 ± 0.382	-0.282 ± 0.459	-0.345 ± 0.435	-0.450 ± 0.294	-0.424 ± 0.250	-0.404 ± 0.267	-0.474 ± 0.259
19	0.088 ± 0.157	0.068 ± 0.112	0.070 ± 0.127	0.085 ± 0.120	-0.396 ± 0.625	-0.281 ± 0.410	-0.306 ± 0.498	-0.370 ± 0.456	-0.459 ± 0.291	-0.436 ± 0.256	-0.415 ± 0.278	-0.491 ± 0.265
20	0.092 ± 0.162	0.074 ± 0.121	0.077 ± 0.141	0.092 ± 0.128	-0.408 ± 0.629	-0.302 ± 0.435	-0.330 ± 0.534	-0.395 ± 0.475	-0.470 ± 0.289	-0.449 ± 0.260	-0.426 ± 0.287	-0.507 ± 0.269
21	0.096 ± 0.166	0.080 ± 0.130	0.085 ± 0.154	0.099 ± 0.135	-0.418 ± 0.628	-0.324 ± 0.459	-0.354 ± 0.568	-0.420 ± 0.491	-0.483 ± 0.287	-0.462 ± 0.262	-0.438 ± 0.294	-0.524 ± 0.271
22	0.100 ± 0.169	0.086 ± 0.138	0.092 ± 0.167	0.106 ± 0.142	-0.427 ± 0.622	-0.345 ± 0.478	-0.377 ± 0.596	-0.445 ± 0.504	-0.497 ± 0.285	-0.476 ± 0.262	-0.452 ± 0.299	-0.542 ± 0.272
23	0.104 ± 0.172	0.092 ± 0.144	0.100 ± 0.179	0.113 ± 0.148	-0.435 ± 0.611	-0.367 ± 0.495	-0.398 ± 0.618	-0.470 ± 0.514	-0.511 ± 0.282	-0.491 ± 0.260	-0.466 ± 0.301	-0.560 ± 0.271
24	0.107 ± 0.173	0.098 ± 0.150	0.106 ± 0.188	0.120 ± 0.152	-0.443 ± 0.596	-0.387 ± 0.506	-0.418 ± 0.633	-0.495 ± 0.520	-0.527 ± 0.278	-0.507 ± 0.255	-0.481 ± 0.301	-0.578 ± 0.269
25	0.110 ± 0.172	0.103 ± 0.154	0.113 ± 0.195	0.127 ± 0.156	-0.450 ± 0.578	-0.407 ± 0.513	-0.436 ± 0.641	-0.519 ± 0.524	-0.543 ± 0.271	-0.523 ± 0.249	-0.497 ± 0.298	-0.596 ± 0.266
26	0.113 ± 0.170	0.108 ± 0.157	0.118 ± 0.200	0.133 ± 0.159	-0.458 ± 0.558	-0.427 ± 0.515	-0.453 ± 0.641	-0.544 ± 0.524	-0.559 ± 0.263	-0.539 ± 0.239	-0.514 ± 0.291	-0.615 ± 0.262
27	0.115 ± 0.166	0.113 ± 0.158	0.123 ± 0.201	0.140 ± 0.161	-0.467 ± 0.536	-0.445 ± 0.511	-0.468 ± 0.633	-0.568 ± 0.521	-0.575 ± 0.253	-0.556 ± 0.228	-0.531 ± 0.280	-0.635 ± 0.257
28	0.117 ± 0.161	0.117 ± 0.157	0.127 ± 0.200	0.146 ± 0.161	-0.477 ± 0.514	-0.463 ± 0.502	-0.482 ± 0.619	-0.592 ± 0.514	-0.592 ± 0.243	-0.573 ± 0.215	-0.549 ± 0.266	-0.654 ± 0.252
29	0.119 ± 0.155	0.121 ± 0.154	0.130 ± 0.196	0.152 ± 0.160	-0.489 ± 0.492	-0.480 ± 0.487	-0.496 ± 0.600	-0.615 ± 0.505	-0.608 ± 0.233	-0.590 ± 0.200	-0.568 ± 0.248	-0.674 ± 0.245
30	0.121 ± 0.149	0.124 ± 0.149	0.133 ± 0.191	0.158 ± 0.158	-0.502 ± 0.470	-0.496 ± 0.467	-0.510 ± 0.577	-0.640 ± 0.493	-0.625 ± 0.225	-0.607 ± 0.184	-0.588 ± 0.227	-0.694 ± 0.237

31	0.124 ± 0.142	0.127 ± 0.143	0.136 ± 0.184	0.164 ± 0.155	-0.517 ± 0.448	-0.513 ± 0.443	-0.525 ± 0.552	-0.664 ± 0.480	-0.643 ± 0.218	-0.625 ± 0.168	-0.609 ± 0.205	-0.715 ± 0.230
32	0.127 ± 0.136	0.131 ± 0.135	0.138 ± 0.175	0.170 ± 0.151	-0.534 ± 0.426	-0.530 ± 0.416	-0.541 ± 0.525	-0.689 ± 0.466	-0.661 ± 0.215	-0.643 ± 0.154	-0.631 ± 0.182	-0.736 ± 0.222
33	0.130 ± 0.129	0.134 ± 0.127	0.142 ± 0.167	0.176 ± 0.146	-0.552 ± 0.404	-0.548 ± 0.386	-0.560 ± 0.497	-0.716 ± 0.450	-0.680 ± 0.214	-0.662 ± 0.144	-0.653 ± 0.159	-0.757 ± 0.215
34	0.134 ± 0.123	0.137 ± 0.118	0.145 ± 0.158	0.182 ± 0.141	-0.572 ± 0.383	-0.568 ± 0.355	-0.580 ± 0.469	-0.743 ± 0.435	-0.699 ± 0.216	-0.682 ± 0.137	-0.677 ± 0.137	-0.779 ± 0.209
35	0.138 ± 0.117	0.142 ± 0.109	0.150 ± 0.149	0.189 ± 0.135	-0.594 ± 0.361	-0.590 ± 0.324	-0.603 ± 0.440	-0.773 ± 0.420	-0.719 ± 0.219	-0.703 ± 0.137	-0.702 ± 0.118	-0.801 ± 0.205
36	0.143 ± 0.111	0.146 ± 0.100	0.154 ± 0.141	0.196 ± 0.129	-0.617 ± 0.340	-0.614 ± 0.293	-0.628 ± 0.412	-0.803 ± 0.405	-0.739 ± 0.225	-0.725 ± 0.142	-0.727 ± 0.104	-0.824 ± 0.203
37	0.148 ± 0.106	0.152 ± 0.092	0.160 ± 0.132	0.204 ± 0.123	-0.642 ± 0.318	-0.641 ± 0.263	-0.656 ± 0.385	-0.836 ± 0.391	-0.759 ± 0.232	-0.748 ± 0.152	-0.753 ± 0.095	-0.848 ± 0.203
38	0.154 ± 0.101	0.159 ± 0.084	0.166 ± 0.124	0.212 ± 0.117	-0.669 ± 0.296	-0.672 ± 0.234	-0.686 ± 0.357	-0.870 ± 0.378	-0.779 ± 0.240	-0.773 ± 0.167	-0.780 ± 0.094	-0.872 ± 0.204
39	0.161 ± 0.095	0.167 ± 0.078	0.173 ± 0.116	0.221 ± 0.111	-0.697 ± 0.274	-0.706 ± 0.208	-0.719 ± 0.329	-0.906 ± 0.367	-0.800 ± 0.249	-0.799 ± 0.184	-0.807 ± 0.100	-0.897 ± 0.208
40	0.168 ± 0.090	0.176 ± 0.072	0.181 ± 0.108	0.230 ± 0.106	-0.728 ± 0.253	-0.743 ± 0.185	-0.755 ± 0.302	-0.944 ± 0.356	-0.820 ± 0.259	-0.826 ± 0.204	-0.835 ± 0.111	-0.923 ± 0.214
41	0.175 ± 0.085	0.186 ± 0.068	0.190 ± 0.101	0.240 ± 0.100	-0.760 ± 0.234	-0.784 ± 0.167	-0.793 ± 0.274	-0.983 ± 0.347	-0.841 ± 0.269	-0.855 ± 0.226	-0.863 ± 0.126	-0.948 ± 0.222
42	0.184 ± 0.081	0.198 ± 0.066	0.200 ± 0.093	0.251 ± 0.095	-0.795 ± 0.217	-0.829 ± 0.155	-0.835 ± 0.246	-1.025 ± 0.341	-0.862 ± 0.279	-0.885 ± 0.249	-0.892 ± 0.142	-0.974 ± 0.231
43	0.193 ± 0.077	0.210 ± 0.066	0.211 ± 0.086	0.262 ± 0.091	-0.833 ± 0.203	-0.877 ± 0.154	-0.879 ± 0.218	-1.069 ± 0.336	-0.883 ± 0.290	-0.916 ± 0.274	-0.921 ± 0.160	-1.000 ± 0.241
44	0.203 ± 0.075	0.224 ± 0.068	0.223 ± 0.079	0.274 ± 0.087	-0.873 ± 0.193	-0.929 ± 0.164	-0.927 ± 0.190	-1.114 ± 0.334	-0.904 ± 0.301	-0.948 ± 0.300	-0.950 ± 0.178	-1.027 ± 0.253
45	0.213 ± 0.073	0.239 ± 0.073	0.237 ± 0.073	0.287 ± 0.085	-0.917 ± 0.188	-0.984 ± 0.185	-0.978 ± 0.163	-1.162 ± 0.334	-0.926 ± 0.312	-0.981 ± 0.327	-0.980 ± 0.196	-1.053 ± 0.265
46	0.225 ± 0.073	0.255 ± 0.080	0.251 ± 0.068	0.301 ± 0.084	-0.963 ± 0.189	-1.042 ± 0.215	-1.032 ± 0.139	-1.213 ± 0.336	-0.949 ± 0.322	-1.015 ± 0.355	-1.010 ± 0.215	-1.080 ± 0.277
47	0.238 ± 0.075	0.272 ± 0.089	0.267 ± 0.063	0.315 ± 0.083	-1.013 ± 0.194	-1.104 ± 0.251	-1.090 ± 0.121	-1.265 ± 0.338	-0.973 ± 0.333	-1.050 ± 0.383	-1.041 ± 0.234	-1.107 ± 0.289
48	0.252 ± 0.077	0.290 ± 0.100	0.284 ± 0.060	0.330 ± 0.084	-1.064 ± 0.204	-1.168 ± 0.293	-1.151 ± 0.113	-1.318 ± 0.341	-0.998 ± 0.343	-1.085 ± 0.412	-1.072 ± 0.253	-1.134 ± 0.301
49	0.267 ± 0.081	0.309 ± 0.112	0.302 ± 0.060	0.346 ± 0.085	-1.118 ± 0.217	-1.235 ± 0.338	-1.215 ± 0.119	-1.374 ± 0.344	-1.023 ± 0.353	-1.121 ± 0.440	-1.103 ± 0.272	-1.162 ± 0.311
50	0.282 ± 0.085	0.329 ± 0.124	0.321 ± 0.062	0.362 ± 0.087	-1.173 ± 0.233	-1.304 ± 0.385	-1.281 ± 0.138	-1.430 ± 0.346	-1.049 ± 0.362	-1.157 ± 0.468	-1.135 ± 0.290	-1.189 ± 0.321

51	0.298 ± 0.090	0.349 ± 0.137	0.340 ± 0.066	0.379 ± 0.089	-1.229 ± 0.252	-1.374 ± 0.432	-1.349 ± 0.167	-1.486 ± 0.348	-1.075 ± 0.370	-1.193 ± 0.495	-1.167 ± 0.309	-1.216 ± 0.329
52	0.314 ± 0.095	0.370 ± 0.151	0.361 ± 0.073	0.396 ± 0.090	-1.286 ± 0.273	-1.445 ± 0.480	-1.419 ± 0.202	-1.543 ± 0.348	-1.101 ± 0.377	-1.228 ± 0.521	-1.198 ± 0.326	-1.242 ± 0.336
53	0.331 ± 0.101	0.390 ± 0.164	0.382 ± 0.081	0.412 ± 0.092	-1.344 ± 0.296	-1.516 ± 0.526	-1.489 ± 0.240	-1.599 ± 0.348	-1.126 ± 0.385	-1.262 ± 0.545	-1.228 ± 0.343	-1.267 ± 0.342
54	0.347 ± 0.107	0.411 ± 0.177	0.403 ± 0.090	0.429 ± 0.093	-1.401 ± 0.319	-1.585 ± 0.570	-1.560 ± 0.280	-1.654 ± 0.346	-1.151 ± 0.391	-1.295 ± 0.566	-1.258 ± 0.359	-1.292 ± 0.346
55	0.364 ± 0.113	0.431 ± 0.189	0.424 ± 0.101	0.446 ± 0.093	-1.459 ± 0.342	-1.653 ± 0.612	-1.629 ± 0.320	-1.709 ± 0.343	-1.175 ± 0.397	-1.325 ± 0.585	-1.286 ± 0.373	-1.315 ± 0.348
56	0.380 ± 0.119	0.450 ± 0.200	0.445 ± 0.111	0.462 ± 0.094	-1.517 ± 0.365	-1.718 ± 0.650	-1.697 ± 0.359	-1.761 ± 0.339	-1.197 ± 0.402	-1.353 ± 0.601	-1.312 ± 0.386	-1.336 ± 0.349
57	0.396 ± 0.125	0.469 ± 0.211	0.465 ± 0.122	0.478 ± 0.094	-1.574 ± 0.386	-1.778 ± 0.684	-1.762 ± 0.397	-1.812 ± 0.335	-1.218 ± 0.406	-1.379 ± 0.614	-1.336 ± 0.396	-1.356 ± 0.348
58	0.412 ± 0.130	0.486 ± 0.220	0.484 ± 0.132	0.493 ± 0.094	-1.631 ± 0.406	-1.834 ± 0.714	-1.824 ± 0.433	-1.861 ± 0.331	-1.238 ± 0.409	-1.401 ± 0.624	-1.358 ± 0.405	-1.374 ± 0.346
59	0.427 ± 0.135	0.501 ± 0.228	0.502 ± 0.142	0.508 ± 0.094	-1.687 ± 0.424	-1.885 ± 0.739	-1.881 ± 0.466	-1.906 ± 0.330	-1.257 ± 0.409	-1.420 ± 0.630	-1.378 ± 0.411	-1.390 ± 0.341
60	0.443 ± 0.140	0.515 ± 0.235	0.518 ± 0.151	0.522 ± 0.096	-1.742 ± 0.442	-1.928 ± 0.760	-1.932 ± 0.496	-1.948 ± 0.331	-1.276 ± 0.408	-1.435 ± 0.632	-1.394 ± 0.414	-1.404 ± 0.336
61	0.458 ± 0.145	0.527 ± 0.241	0.533 ± 0.160	0.534 ± 0.099	-1.797 ± 0.460	-1.965 ± 0.775	-1.978 ± 0.523	-1.987 ± 0.338	-1.293 ± 0.404	-1.446 ± 0.631	-1.407 ± 0.415	-1.416 ± 0.329
62	0.473 ± 0.150	0.536 ± 0.245	0.546 ± 0.168	0.546 ± 0.103	-1.849 ± 0.479	-1.995 ± 0.786	-2.016 ± 0.547	-2.020 ± 0.350	-1.310 ± 0.398	-1.452 ± 0.627	-1.416 ± 0.414	-1.425 ± 0.322
63	0.487 ± 0.155	0.544 ± 0.248	0.556 ± 0.175	0.555 ± 0.110	-1.899 ± 0.501	-2.017 ± 0.791	-2.047 ± 0.567	-2.048 ± 0.368	-1.326 ± 0.390	-1.454 ± 0.620	-1.422 ± 0.410	-1.431 ± 0.315
64	0.500 ± 0.161	0.548 ± 0.249	0.564 ± 0.181	0.563 ± 0.118	-1.946 ± 0.528	-2.030 ± 0.793	-2.069 ± 0.584	-2.070 ± 0.392	-1.341 ± 0.382	-1.452 ± 0.609	-1.424 ± 0.404	-1.434 ± 0.309
65	0.513 ± 0.169	0.551 ± 0.250	0.570 ± 0.187	0.569 ± 0.127	-1.989 ± 0.558	-2.036 ± 0.789	-2.084 ± 0.598	-2.085 ± 0.419	-1.355 ± 0.373	-1.446 ± 0.596	-1.423 ± 0.396	-1.434 ± 0.304
66	0.524 ± 0.177	0.551 ± 0.249	0.572 ± 0.191	0.573 ± 0.136	-2.027 ± 0.594	-2.035 ± 0.782	-2.089 ± 0.607	-2.092 ± 0.448	-1.367 ± 0.364	-1.436 ± 0.581	-1.418 ± 0.386	-1.430 ± 0.300
67	0.534 ± 0.186	0.549 ± 0.247	0.572 ± 0.194	0.575 ± 0.146	-2.059 ± 0.634	-2.027 ± 0.771	-2.086 ± 0.614	-2.092 ± 0.477	-1.378 ± 0.358	-1.423 ± 0.564	-1.409 ± 0.375	-1.422 ± 0.297
68	0.542 ± 0.197	0.545 ± 0.244	0.570 ± 0.196	0.573 ± 0.155	-2.083 ± 0.678	-2.011 ± 0.757	-2.074 ± 0.616	-2.084 ± 0.504	-1.387 ± 0.355	-1.406 ± 0.545	-1.396 ± 0.363	-1.411 ± 0.295
69	0.548 ± 0.208	0.539 ± 0.239	0.564 ± 0.196	0.569 ± 0.163	-2.100 ± 0.724	-1.990 ± 0.740	-2.053 ± 0.616	-2.067 ± 0.528	-1.392 ± 0.354	-1.386 ± 0.525	-1.379 ± 0.350	-1.396 ± 0.294
70	0.551 ± 0.219	0.531 ± 0.234	0.556 ± 0.195	0.563 ± 0.170	-2.108 ± 0.771	-1.964 ± 0.721	-2.023 ± 0.612	-2.042 ± 0.548	-1.393 ± 0.357	-1.364 ± 0.504	-1.359 ± 0.336	-1.378 ± 0.292

71	0.552 ± 0.231	0.522 ± 0.229	0.545 ± 0.194	0.554 ± 0.176	-2.106 ± 0.818	-1.934 ± 0.699	-1.986 ± 0.605	-2.011 ± 0.564	-1.391 ± 0.362	-1.341 ± 0.482	-1.337 ± 0.322	-1.357 ± 0.290
72	0.550 ± 0.243	0.512 ± 0.222	0.531 ± 0.191	0.542 ± 0.180	-2.096 ± 0.864	-1.900 ± 0.675	-1.943 ± 0.595	-1.972 ± 0.575	-1.384 ± 0.369	-1.316 ± 0.459	-1.311 ± 0.308	-1.333 ± 0.287
73	0.546 ± 0.255	0.501 ± 0.215	0.516 ± 0.188	0.529 ± 0.183	-2.078 ± 0.910	-1.864 ± 0.649	-1.893 ± 0.585	-1.928 ± 0.582	-1.372 ± 0.377	-1.290 ± 0.436	-1.283 ± 0.294	-1.306 ± 0.284
74	0.538 ± 0.266	0.489 ± 0.207	0.499 ± 0.184	0.514 ± 0.184	-2.052 ± 0.954	-1.826 ± 0.622	-1.839 ± 0.574	-1.879 ± 0.586	-1.357 ± 0.385	-1.264 ± 0.412	-1.253 ± 0.281	-1.278 ± 0.279
75	0.529 ± 0.278	0.477 ± 0.198	0.482 ± 0.181	0.498 ± 0.185	-2.021 ± 0.995	-1.787 ± 0.594	-1.782 ± 0.564	-1.826 ± 0.587	-1.338 ± 0.393	-1.238 ± 0.389	-1.221 ± 0.269	-1.248 ± 0.273
76	0.518 ± 0.289	0.464 ± 0.190	0.463 ± 0.177	0.481 ± 0.185	-1.985 ± 1.032	-1.747 ± 0.566	-1.723 ± 0.556	-1.771 ± 0.587	-1.316 ± 0.401	-1.212 ± 0.366	-1.188 ± 0.258	-1.218 ± 0.266
77	0.506 ± 0.300	0.452 ± 0.181	0.444 ± 0.174	0.464 ± 0.185	-1.946 ± 1.064	-1.707 ± 0.539	-1.664 ± 0.550	-1.715 ± 0.586	-1.293 ± 0.409	-1.187 ± 0.344	-1.155 ± 0.250	-1.186 ± 0.260
78	0.493 ± 0.309	0.439 ± 0.173	0.426 ± 0.172	0.446 ± 0.184	-1.906 ± 1.089	-1.666 ± 0.514	-1.605 ± 0.547	-1.659 ± 0.585	-1.268 ± 0.416	-1.162 ± 0.323	-1.122 ± 0.243	-1.155 ± 0.253
79	0.480 ± 0.317	0.426 ± 0.165	0.408 ± 0.170	0.429 ± 0.183	-1.864 ± 1.107	-1.625 ± 0.491	-1.548 ± 0.547	-1.604 ± 0.585	-1.243 ± 0.422	-1.137 ± 0.305	-1.089 ± 0.239	-1.124 ± 0.248
80	0.468 ± 0.322	0.413 ± 0.158	0.391 ± 0.169	0.413 ± 0.182	-1.822 ± 1.118	-1.585 ± 0.473	-1.494 ± 0.549	-1.551 ± 0.585	-1.217 ± 0.426	-1.113 ± 0.289	-1.056 ± 0.238	-1.093 ± 0.244
81	0.456 ± 0.326	0.400 ± 0.153	0.375 ± 0.168	0.398 ± 0.181	-1.779 ± 1.122	-1.544 ± 0.459	-1.443 ± 0.553	-1.501 ± 0.587	-1.192 ± 0.429	-1.089 ± 0.276	-1.025 ± 0.239	-1.063 ± 0.242
82	0.444 ± 0.327	0.387 ± 0.148	0.361 ± 0.167	0.383 ± 0.180	-1.737 ± 1.119	-1.504 ± 0.450	-1.396 ± 0.558	-1.453 ± 0.590	-1.166 ± 0.431	-1.066 ± 0.265	-0.994 ± 0.242	-1.033 ± 0.243
83	0.433 ± 0.326	0.375 ± 0.145	0.347 ± 0.166	0.370 ± 0.178	-1.696 ± 1.110	-1.464 ± 0.446	-1.352 ± 0.563	-1.408 ± 0.594	-1.140 ± 0.433	-1.043 ± 0.257	-0.966 ± 0.247	-1.003 ± 0.246
84	0.422 ± 0.323	0.363 ± 0.144	0.335 ± 0.165	0.358 ± 0.177	-1.655 ± 1.096	-1.425 ± 0.447	-1.313 ± 0.568	-1.367 ± 0.598	-1.114 ± 0.435	-1.020 ± 0.252	-0.938 ± 0.253	-0.975 ± 0.252
85	0.412 ± 0.318	0.351 ± 0.144	0.324 ± 0.164	0.347 ± 0.175	-1.615 ± 1.078	-1.387 ± 0.453	-1.277 ± 0.573	-1.329 ± 0.603	-1.087 ± 0.436	-0.997 ± 0.250	-0.913 ± 0.260	-0.948 ± 0.260
86	0.402 ± 0.313	0.340 ± 0.144	0.315 ± 0.163	0.337 ± 0.174	-1.575 ± 1.057	-1.350 ± 0.462	-1.245 ± 0.576	-1.294 ± 0.607	-1.061 ± 0.438	-0.975 ± 0.251	-0.889 ± 0.266	-0.923 ± 0.269
87	0.393 ± 0.306	0.329 ± 0.146	0.306 ± 0.161	0.328 ± 0.172	-1.537 ± 1.035	-1.315 ± 0.475	-1.215 ± 0.579	-1.263 ± 0.611	-1.036 ± 0.440	-0.954 ± 0.253	-0.868 ± 0.273	-0.899 ± 0.280
88	0.383 ± 0.299	0.319 ± 0.148	0.298 ± 0.160	0.320 ± 0.170	-1.501 ± 1.011	-1.283 ± 0.488	-1.189 ± 0.580	-1.236 ± 0.615	-1.011 ± 0.442	-0.933 ± 0.258	-0.848 ± 0.278	-0.877 ± 0.290
89	0.374 ± 0.292	0.311 ± 0.150	0.291 ± 0.158	0.313 ± 0.168	-1.466 ± 0.986	-1.252 ± 0.503	-1.166 ± 0.581	-1.211 ± 0.617	-0.988 ± 0.443	-0.913 ± 0.265	-0.830 ± 0.282	-0.857 ± 0.299
90	0.366 ± 0.284	0.302 ± 0.152	0.284 ± 0.156	0.306 ± 0.166	-1.433 ± 0.961	-1.224 ± 0.517	-1.144 ± 0.580	-1.189 ± 0.618	-0.966 ± 0.443	-0.893 ± 0.274	-0.814 ± 0.286	-0.839 ± 0.308

91	0.358 ± 0.277	0.295 ± 0.153	0.278 ± 0.154	0.301 ± 0.164	-1.402 ± 0.936	-1.198 ± 0.531	-1.125 ± 0.579	-1.170 ± 0.618	-0.946 ± 0.441	-0.874 ± 0.283	-0.799 ± 0.288	-0.822 ± 0.314
92	0.350 ± 0.269	0.289 ± 0.154	0.273 ± 0.152	0.296 ± 0.162	-1.373 ± 0.911	-1.175 ± 0.543	-1.108 ± 0.577	-1.152 ± 0.617	-0.927 ± 0.438	-0.857 ± 0.293	-0.786 ± 0.289	-0.808 ± 0.320
93	0.343 ± 0.261	0.283 ± 0.155	0.268 ± 0.150	0.291 ± 0.160	-1.346 ± 0.887	-1.153 ± 0.554	-1.092 ± 0.574	-1.137 ± 0.615	-0.910 ± 0.434	-0.840 ± 0.302	-0.774 ± 0.289	-0.796 ± 0.324
94	0.336 ± 0.254	0.277 ± 0.156	0.264 ± 0.148	0.287 ± 0.158	-1.320 ± 0.864	-1.134 ± 0.563	-1.077 ± 0.571	-1.123 ± 0.612	-0.894 ± 0.429	-0.825 ± 0.311	-0.764 ± 0.288	-0.784 ± 0.326
95	0.330 ± 0.246	0.273 ± 0.156	0.259 ± 0.145	0.283 ± 0.156	-1.295 ± 0.841	-1.115 ± 0.570	-1.063 ± 0.567	-1.110 ± 0.609	-0.878 ± 0.424	-0.810 ± 0.319	-0.754 ± 0.285	-0.774 ± 0.327
96	0.323 ± 0.239	0.268 ± 0.155	0.255 ± 0.143	0.279 ± 0.154	-1.271 ± 0.820	-1.099 ± 0.576	-1.050 ± 0.563	-1.097 ± 0.606	-0.864 ± 0.418	-0.797 ± 0.326	-0.744 ± 0.283	-0.765 ± 0.328
97	0.316 ± 0.232	0.264 ± 0.155	0.251 ± 0.141	0.275 ± 0.152	-1.248 ± 0.798	-1.083 ± 0.580	-1.037 ± 0.558	-1.085 ± 0.601	-0.850 ± 0.412	-0.784 ± 0.332	-0.736 ± 0.279	-0.755 ± 0.327
98	0.310 ± 0.225	0.260 ± 0.154	0.247 ± 0.139	0.272 ± 0.150	-1.224 ± 0.777	-1.067 ± 0.584	-1.024 ± 0.553	-1.074 ± 0.597	-0.835 ± 0.406	-0.771 ± 0.338	-0.727 ± 0.275	-0.747 ± 0.327
99	0.303 ± 0.218	0.255 ± 0.153	0.243 ± 0.137	0.268 ± 0.148	-1.201 ± 0.757	-1.051 ± 0.588	-1.011 ± 0.549	-1.062 ± 0.593	-0.821 ± 0.400	-0.758 ± 0.344	-0.718 ± 0.271	-0.738 ± 0.326
100	0.297 ± 0.211	0.251 ± 0.152	0.239 ± 0.134	0.264 ± 0.146	-1.177 ± 0.737	-1.035 ± 0.593	-0.998 ± 0.544	-1.050 ± 0.589	-0.807 ± 0.395	-0.745 ± 0.350	-0.710 ± 0.268	-0.729 ± 0.326

Tab. S6 Full normalized knee flexion moment data during walking under four knee protector conditions (group mean ± SD, n = 5).

Percent cycle (%)	Pro.off	Pro.a	Pro.b	Pro.c
0	0.0002 ± 0.0003	0.0002 ± 0.0006	0.0003 ± 0.0004	0.0004 ± 0.0006
1	0.0002 ± 0.0003	0.0001 ± 0.0007	0.0003 ± 0.0004	0.0003 ± 0.0006
2	0.0001 ± 0.0003	0.0000 ± 0.0008	0.0002 ± 0.0004	0.0001 ± 0.0006
3	0.0001 ± 0.0003	0.0000 ± 0.0007	0.0001 ± 0.0005	-0.0001 ± 0.0008
4	-0.0000 ± 0.0003	-0.0000 ± 0.0007	-0.0000 ± 0.0007	-0.0002 ± 0.0011
5	-0.0001 ± 0.0004	-0.0001 ± 0.0005	-0.0002 ± 0.0009	-0.0004 ± 0.0014
6	-0.0002 ± 0.0005	-0.0002 ± 0.0004	-0.0003 ± 0.0010	-0.0005 ± 0.0015
7	-0.0003 ± 0.0006	-0.0002 ± 0.0004	-0.0005 ± 0.0011	-0.0005 ± 0.0015
8	-0.0004 ± 0.0006	-0.0004 ± 0.0006	-0.0007 ± 0.0011	-0.0006 ± 0.0013
9	-0.0005 ± 0.0007	-0.0005 ± 0.0007	-0.0008 ± 0.0009	-0.0006 ± 0.0011
10	-0.0006 ± 0.0007	-0.0007 ± 0.0008	-0.0010 ± 0.0007	-0.0007 ± 0.0008
11	-0.0007 ± 0.0007	-0.0009 ± 0.0008	-0.0010 ± 0.0004	-0.0007 ± 0.0006
12	-0.0009 ± 0.0007	-0.0011 ± 0.0007	-0.0011 ± 0.0003	-0.0008 ± 0.0007
13	-0.0011 ± 0.0007	-0.0013 ± 0.0008	-0.0011 ± 0.0005	-0.0008 ± 0.0009
14	-0.0013 ± 0.0006	-0.0015 ± 0.0011	-0.0010 ± 0.0007	-0.0008 ± 0.0012
15	-0.0015 ± 0.0007	-0.0016 ± 0.0014	-0.0008 ± 0.0009	-0.0008 ± 0.0015
16	-0.0016 ± 0.0008	-0.0015 ± 0.0018	-0.0007 ± 0.0010	-0.0008 ± 0.0016
17	-0.0016 ± 0.0010	-0.0014 ± 0.0020	-0.0005 ± 0.0012	-0.0007 ± 0.0017
18	-0.0015 ± 0.0011	-0.0012 ± 0.0022	-0.0004 ± 0.0013	-0.0006 ± 0.0017
19	-0.0012 ± 0.0012	-0.0008 ± 0.0022	-0.0002 ± 0.0014	-0.0004 ± 0.0014
20	-0.0009 ± 0.0011	-0.0004 ± 0.0021	-0.0001 ± 0.0015	-0.0002 ± 0.0012
21	-0.0004 ± 0.0009	0.0001 ± 0.0019	-0.0001 ± 0.0016	-0.0000 ± 0.0009

22	0.0001 ± 0.0007	0.0005 ± 0.0017	0.0000 ± 0.0015	0.0002 ± 0.0009
23	0.0007 ± 0.0004	0.0010 ± 0.0014	0.0001 ± 0.0014	0.0005 ± 0.0012
24	0.0013 ± 0.0003	0.0014 ± 0.0013	0.0002 ± 0.0012	0.0007 ± 0.0014
25	0.0017 ± 0.0006	0.0018 ± 0.0012	0.0003 ± 0.0011	0.0009 ± 0.0015
26	0.0020 ± 0.0009	0.0020 ± 0.0013	0.0005 ± 0.0011	0.0011 ± 0.0015
27	0.0021 ± 0.0010	0.0021 ± 0.0013	0.0006 ± 0.0012	0.0012 ± 0.0015
28	0.0019 ± 0.0011	0.0020 ± 0.0012	0.0007 ± 0.0013	0.0012 ± 0.0015
29	0.0016 ± 0.0011	0.0017 ± 0.0010	0.0007 ± 0.0014	0.0011 ± 0.0016
30	0.0011 ± 0.0011	0.0012 ± 0.0009	0.0007 ± 0.0013	0.0009 ± 0.0016
31	0.0005 ± 0.0011	0.0006 ± 0.0009	0.0006 ± 0.0012	0.0007 ± 0.0016
32	-0.0001 ± 0.0010	0.0001 ± 0.0010	0.0005 ± 0.0010	0.0005 ± 0.0014
33	-0.0006 ± 0.0010	-0.0004 ± 0.0012	0.0003 ± 0.0008	0.0002 ± 0.0012
34	-0.0009 ± 0.0009	-0.0008 ± 0.0013	0.0001 ± 0.0005	0.0000 ± 0.0009
35	-0.0011 ± 0.0009	-0.0010 ± 0.0013	-0.0000 ± 0.0004	-0.0001 ± 0.0007
36	-0.0011 ± 0.0010	-0.0011 ± 0.0013	-0.0002 ± 0.0004	-0.0002 ± 0.0006
37	-0.0010 ± 0.0010	-0.0010 ± 0.0013	-0.0003 ± 0.0006	-0.0002 ± 0.0006
38	-0.0008 ± 0.0010	-0.0009 ± 0.0012	-0.0003 ± 0.0008	-0.0002 ± 0.0007
39	-0.0006 ± 0.0010	-0.0007 ± 0.0011	-0.0003 ± 0.0009	-0.0002 ± 0.0007
40	-0.0004 ± 0.0010	-0.0005 ± 0.0010	-0.0003 ± 0.0009	-0.0002 ± 0.0008
41	-0.0003 ± 0.0009	-0.0004 ± 0.0010	-0.0003 ± 0.0009	-0.0002 ± 0.0008
42	-0.0002 ± 0.0009	-0.0003 ± 0.0010	-0.0002 ± 0.0009	-0.0001 ± 0.0008
43	-0.0001 ± 0.0008	-0.0003 ± 0.0010	-0.0001 ± 0.0008	-0.0001 ± 0.0008
44	-0.0001 ± 0.0008	-0.0002 ± 0.0011	-0.0000 ± 0.0008	-0.0001 ± 0.0008
45	-0.0000 ± 0.0008	-0.0002 ± 0.0011	0.0000 ± 0.0008	-0.0000 ± 0.0008
46	0.0000 ± 0.0008	-0.0001 ± 0.0011	0.0001 ± 0.0008	0.0000 ± 0.0007
47	0.0001 ± 0.0007	-0.0000 ± 0.0010	0.0002 ± 0.0008	0.0001 ± 0.0007
48	0.0001 ± 0.0007	0.0000 ± 0.0010	0.0002 ± 0.0008	0.0001 ± 0.0007
49	0.0002 ± 0.0007	0.0001 ± 0.0009	0.0003 ± 0.0008	0.0002 ± 0.0007
50	0.0002 ± 0.0007	0.0002 ± 0.0009	0.0004 ± 0.0008	0.0003 ± 0.0006
51	0.0003 ± 0.0006	0.0003 ± 0.0009	0.0005 ± 0.0008	0.0004 ± 0.0006
52	0.0004 ± 0.0006	0.0004 ± 0.0008	0.0005 ± 0.0008	0.0005 ± 0.0006
53	0.0004 ± 0.0006	0.0005 ± 0.0008	0.0006 ± 0.0009	0.0006 ± 0.0006
54	0.0005 ± 0.0006	0.0006 ± 0.0008	0.0007 ± 0.0009	0.0007 ± 0.0006
55	0.0006 ± 0.0006	0.0007 ± 0.0009	0.0008 ± 0.0009	0.0008 ± 0.0006
56	0.0007 ± 0.0007	0.0008 ± 0.0009	0.0008 ± 0.0009	0.0009 ± 0.0007
57	0.0008 ± 0.0007	0.0008 ± 0.0009	0.0009 ± 0.0009	0.0010 ± 0.0007
58	0.0009 ± 0.0007	0.0009 ± 0.0009	0.0010 ± 0.0009	0.0011 ± 0.0007
59	0.0010 ± 0.0007	0.0009 ± 0.0009	0.0010 ± 0.0009	0.0012 ± 0.0006
60	0.0012 ± 0.0007	0.0009 ± 0.0009	0.0011 ± 0.0008	0.0012 ± 0.0006
61	0.0013 ± 0.0007	0.0009 ± 0.0009	0.0011 ± 0.0008	0.0013 ± 0.0006
62	0.0014 ± 0.0007	0.0009 ± 0.0010	0.0011 ± 0.0007	0.0013 ± 0.0005
63	0.0014 ± 0.0007	0.0009 ± 0.0011	0.0010 ± 0.0008	0.0013 ± 0.0004
64	0.0014 ± 0.0007	0.0008 ± 0.0012	0.0010 ± 0.0009	0.0012 ± 0.0004
65	0.0013 ± 0.0006	0.0007 ± 0.0012	0.0009 ± 0.0010	0.0011 ± 0.0004
66	0.0011 ± 0.0006	0.0006 ± 0.0012	0.0008 ± 0.0012	0.0010 ± 0.0005
67	0.0009 ± 0.0006	0.0005 ± 0.0012	0.0007 ± 0.0013	0.0008 ± 0.0007
68	0.0007 ± 0.0006	0.0004 ± 0.0010	0.0007 ± 0.0012	0.0006 ± 0.0007
69	0.0005 ± 0.0007	0.0003 ± 0.0009	0.0007 ± 0.0010	0.0004 ± 0.0008
70	0.0003 ± 0.0008	0.0002 ± 0.0007	0.0007 ± 0.0006	0.0002 ± 0.0007
71	0.0002 ± 0.0009	0.0001 ± 0.0006	0.0007 ± 0.0002	-0.0000 ± 0.0006
72	0.0001 ± 0.0009	0.0000 ± 0.0005	0.0007 ± 0.0003	-0.0001 ± 0.0004
73	0.0000 ± 0.0009	-0.0000 ± 0.0005	0.0006 ± 0.0007	-0.0002 ± 0.0003
74	-0.0000 ± 0.0009	-0.0000 ± 0.0007	0.0006 ± 0.0011	-0.0003 ± 0.0003
75	-0.0000 ± 0.0009	0.0001 ± 0.0010	0.0005 ± 0.0014	-0.0002 ± 0.0006
76	-0.0000 ± 0.0008	0.0002 ± 0.0013	0.0004 ± 0.0017	-0.0001 ± 0.0009
77	0.0001 ± 0.0009	0.0004 ± 0.0017	0.0004 ± 0.0019	0.0002 ± 0.0013
78	0.0002 ± 0.0010	0.0007 ± 0.0019	0.0005 ± 0.0022	0.0005 ± 0.0016
79	0.0004 ± 0.0012	0.0010 ± 0.0020	0.0007 ± 0.0024	0.0010 ± 0.0019
80	0.0007 ± 0.0014	0.0013 ± 0.0019	0.0010 ± 0.0025	0.0015 ± 0.0021
81	0.0012 ± 0.0016	0.0016 ± 0.0016	0.0013 ± 0.0026	0.0021 ± 0.0023

82	0.0017 ± 0.0018	0.0019 ± 0.0012	0.0017 ± 0.0025	0.0026 ± 0.0025
83	0.0023 ± 0.0019	0.0022 ± 0.0008	0.0021 ± 0.0023	0.0032 ± 0.0025
84	0.0028 ± 0.0020	0.0026 ± 0.0005	0.0024 ± 0.0018	0.0037 ± 0.0024
85	0.0033 ± 0.0019	0.0031 ± 0.0005	0.0027 ± 0.0013	0.0040 ± 0.0020
86	0.0037 ± 0.0016	0.0036 ± 0.0007	0.0030 ± 0.0011	0.0042 ± 0.0014
87	0.0039 ± 0.0013	0.0040 ± 0.0010	0.0032 ± 0.0014	0.0042 ± 0.0007
88	0.0040 ± 0.0011	0.0043 ± 0.0013	0.0033 ± 0.0019	0.0041 ± 0.0008
89	0.0040 ± 0.0013	0.0044 ± 0.0017	0.0034 ± 0.0023	0.0039 ± 0.0015
90	0.0039 ± 0.0019	0.0042 ± 0.0020	0.0034 ± 0.0026	0.0035 ± 0.0022
91	0.0037 ± 0.0025	0.0037 ± 0.0023	0.0033 ± 0.0028	0.0030 ± 0.0026
92	0.0034 ± 0.0029	0.0030 ± 0.0024	0.0031 ± 0.0028	0.0025 ± 0.0028
93	0.0030 ± 0.0032	0.0021 ± 0.0023	0.0027 ± 0.0028	0.0019 ± 0.0027
94	0.0025 ± 0.0032	0.0012 ± 0.0020	0.0023 ± 0.0028	0.0014 ± 0.0024
95	0.0019 ± 0.0029	0.0004 ± 0.0016	0.0018 ± 0.0027	0.0008 ± 0.0020
96	0.0014 ± 0.0025	-0.0002 ± 0.0012	0.0013 ± 0.0025	0.0004 ± 0.0014
97	0.0008 ± 0.0019	-0.0006 ± 0.0010	0.0008 ± 0.0021	0.0000 ± 0.0009
98	0.0003 ± 0.0013	-0.0007 ± 0.0008	0.0003 ± 0.0015	-0.0001 ± 0.0003
99	-0.0002 ± 0.0007	-0.0004 ± 0.0007	0.0000 ± 0.0005	-0.0001 ± 0.0004
100	-0.0006 ± 0.0010	0.0004 ± 0.0008	-0.0002 ± 0.0010	0.0003 ± 0.0012

Tab. S7 Full normalized knee flexion moment data during jogging under four knee protector conditions (group mean ± SD, n = 5).

Percent cycle (%)	Pro.off	Pro.a	Pro.b	Pro.c
0	0.0027 ± 0.0013	0.0023 ± 0.0015	0.0025 ± 0.0015	0.0027 ± 0.0018
1	0.0026 ± 0.0013	0.0022 ± 0.0015	0.0024 ± 0.0015	0.0026 ± 0.0018
2	0.0025 ± 0.0013	0.0022 ± 0.0014	0.0024 ± 0.0015	0.0025 ± 0.0018
3	0.0025 ± 0.0013	0.0021 ± 0.0014	0.0023 ± 0.0014	0.0024 ± 0.0018
4	0.0024 ± 0.0012	0.0020 ± 0.0014	0.0022 ± 0.0014	0.0023 ± 0.0018
5	0.0023 ± 0.0012	0.0019 ± 0.0014	0.0021 ± 0.0014	0.0022 ± 0.0017
6	0.0023 ± 0.0012	0.0018 ± 0.0014	0.0020 ± 0.0014	0.0021 ± 0.0017
7	0.0022 ± 0.0012	0.0017 ± 0.0014	0.0020 ± 0.0014	0.0020 ± 0.0017
8	0.0021 ± 0.0011	0.0017 ± 0.0013	0.0019 ± 0.0014	0.0019 ± 0.0017
9	0.0021 ± 0.0011	0.0016 ± 0.0013	0.0018 ± 0.0014	0.0019 ± 0.0017
10	0.0020 ± 0.0011	0.0015 ± 0.0013	0.0017 ± 0.0014	0.0018 ± 0.0017
11	0.0020 ± 0.0011	0.0014 ± 0.0013	0.0017 ± 0.0014	0.0017 ± 0.0017
12	0.0019 ± 0.0010	0.0013 ± 0.0013	0.0016 ± 0.0014	0.0016 ± 0.0017
13	0.0018 ± 0.0010	0.0013 ± 0.0013	0.0015 ± 0.0014	0.0015 ± 0.0017
14	0.0018 ± 0.0010	0.0012 ± 0.0013	0.0014 ± 0.0014	0.0014 ± 0.0017
15	0.0017 ± 0.0010	0.0011 ± 0.0013	0.0013 ± 0.0014	0.0013 ± 0.0017
16	0.0016 ± 0.0009	0.0010 ± 0.0013	0.0013 ± 0.0014	0.0012 ± 0.0017
17	0.0016 ± 0.0009	0.0009 ± 0.0013	0.0012 ± 0.0014	0.0011 ± 0.0017
18	0.0015 ± 0.0009	0.0008 ± 0.0013	0.0011 ± 0.0014	0.0010 ± 0.0017
19	0.0014 ± 0.0009	0.0008 ± 0.0013	0.0010 ± 0.0014	0.0009 ± 0.0017
20	0.0014 ± 0.0008	0.0007 ± 0.0013	0.0009 ± 0.0014	0.0008 ± 0.0017
21	0.0013 ± 0.0008	0.0006 ± 0.0013	0.0009 ± 0.0014	0.0008 ± 0.0017
22	0.0013 ± 0.0008	0.0005 ± 0.0013	0.0008 ± 0.0014	0.0007 ± 0.0017
23	0.0012 ± 0.0007	0.0004 ± 0.0013	0.0007 ± 0.0015	0.0006 ± 0.0017
24	0.0011 ± 0.0007	0.0004 ± 0.0013	0.0006 ± 0.0015	0.0005 ± 0.0017
25	0.0011 ± 0.0007	0.0003 ± 0.0013	0.0005 ± 0.0015	0.0004 ± 0.0017
26	0.0010 ± 0.0006	0.0002 ± 0.0013	0.0004 ± 0.0015	0.0003 ± 0.0017
27	0.0010 ± 0.0006	0.0001 ± 0.0013	0.0004 ± 0.0015	0.0003 ± 0.0017
28	0.0009 ± 0.0006	0.0001 ± 0.0013	0.0003 ± 0.0016	0.0002 ± 0.0017
29	0.0008 ± 0.0005	-0.0000 ± 0.0013	0.0002 ± 0.0016	0.0001 ± 0.0017
30	0.0008 ± 0.0005	-0.0001 ± 0.0014	0.0001 ± 0.0016	0.0000 ± 0.0017
31	0.0007 ± 0.0004	-0.0001 ± 0.0014	0.0000 ± 0.0016	-0.0000 ± 0.0018
32	0.0007 ± 0.0004	-0.0002 ± 0.0014	-0.0000 ± 0.0017	-0.0001 ± 0.0018

33	0.0006 ± 0.0004	-0.0003 ± 0.0014	-0.0001 ± 0.0017	-0.0002 ± 0.0018
34	0.0006 ± 0.0003	-0.0003 ± 0.0015	-0.0002 ± 0.0017	-0.0002 ± 0.0018
35	0.0005 ± 0.0003	-0.0004 ± 0.0015	-0.0002 ± 0.0017	-0.0003 ± 0.0018
36	0.0005 ± 0.0003	-0.0004 ± 0.0015	-0.0003 ± 0.0018	-0.0003 ± 0.0019
37	0.0005 ± 0.0002	-0.0005 ± 0.0016	-0.0004 ± 0.0018	-0.0004 ± 0.0019
38	0.0004 ± 0.0002	-0.0005 ± 0.0016	-0.0004 ± 0.0019	-0.0004 ± 0.0019
39	0.0004 ± 0.0002	-0.0006 ± 0.0017	-0.0005 ± 0.0019	-0.0004 ± 0.0020
40	0.0004 ± 0.0002	-0.0006 ± 0.0017	-0.0005 ± 0.0019	-0.0005 ± 0.0020
41	0.0004 ± 0.0003	-0.0006 ± 0.0018	-0.0005 ± 0.0020	-0.0005 ± 0.0020
42	0.0004 ± 0.0003	-0.0006 ± 0.0018	-0.0006 ± 0.0020	-0.0005 ± 0.0021
43	0.0004 ± 0.0004	-0.0006 ± 0.0019	-0.0006 ± 0.0021	-0.0005 ± 0.0021
44	0.0004 ± 0.0004	-0.0006 ± 0.0019	-0.0006 ± 0.0021	-0.0005 ± 0.0021
45	0.0004 ± 0.0004	-0.0006 ± 0.0020	-0.0006 ± 0.0022	-0.0005 ± 0.0022
46	0.0004 ± 0.0005	-0.0006 ± 0.0021	-0.0006 ± 0.0022	-0.0005 ± 0.0022
47	0.0004 ± 0.0005	-0.0006 ± 0.0021	-0.0006 ± 0.0022	-0.0004 ± 0.0022
48	0.0005 ± 0.0006	-0.0006 ± 0.0022	-0.0006 ± 0.0023	-0.0004 ± 0.0023
49	0.0005 ± 0.0006	-0.0005 ± 0.0022	-0.0005 ± 0.0023	-0.0003 ± 0.0023
50	0.0006 ± 0.0007	-0.0005 ± 0.0023	-0.0005 ± 0.0024	-0.0003 ± 0.0024
51	0.0006 ± 0.0007	-0.0004 ± 0.0024	-0.0004 ± 0.0024	-0.0002 ± 0.0024
52	0.0007 ± 0.0007	-0.0004 ± 0.0024	-0.0004 ± 0.0025	-0.0001 ± 0.0024
53	0.0008 ± 0.0008	-0.0003 ± 0.0025	-0.0003 ± 0.0025	-0.0001 ± 0.0025
54	0.0009 ± 0.0008	-0.0002 ± 0.0025	-0.0002 ± 0.0025	0.0000 ± 0.0025
55	0.0010 ± 0.0008	-0.0001 ± 0.0026	-0.0001 ± 0.0026	0.0001 ± 0.0025
56	0.0011 ± 0.0009	-0.0000 ± 0.0026	-0.0000 ± 0.0026	0.0003 ± 0.0026
57	0.0012 ± 0.0009	0.0001 ± 0.0027	0.0001 ± 0.0027	0.0004 ± 0.0026
58	0.0013 ± 0.0009	0.0002 ± 0.0027	0.0002 ± 0.0027	0.0005 ± 0.0026
59	0.0015 ± 0.0009	0.0003 ± 0.0028	0.0004 ± 0.0027	0.0007 ± 0.0027
60	0.0016 ± 0.0009	0.0005 ± 0.0028	0.0005 ± 0.0028	0.0008 ± 0.0027
61	0.0018 ± 0.0009	0.0006 ± 0.0028	0.0007 ± 0.0028	0.0010 ± 0.0027
62	0.0020 ± 0.0009	0.0008 ± 0.0029	0.0009 ± 0.0028	0.0012 ± 0.0027
63	0.0022 ± 0.0009	0.0010 ± 0.0029	0.0011 ± 0.0028	0.0013 ± 0.0028
64	0.0023 ± 0.0009	0.0012 ± 0.0029	0.0013 ± 0.0028	0.0015 ± 0.0028
65	0.0026 ± 0.0009	0.0014 ± 0.0029	0.0015 ± 0.0029	0.0017 ± 0.0028
66	0.0028 ± 0.0009	0.0016 ± 0.0029	0.0017 ± 0.0029	0.0020 ± 0.0028
67	0.0030 ± 0.0009	0.0018 ± 0.0029	0.0019 ± 0.0029	0.0022 ± 0.0028
68	0.0032 ± 0.0008	0.0020 ± 0.0029	0.0022 ± 0.0029	0.0024 ± 0.0028
69	0.0035 ± 0.0008	0.0022 ± 0.0029	0.0025 ± 0.0029	0.0027 ± 0.0028
70	0.0037 ± 0.0008	0.0025 ± 0.0029	0.0027 ± 0.0029	0.0029 ± 0.0029
71	0.0040 ± 0.0008	0.0027 ± 0.0029	0.0030 ± 0.0029	0.0032 ± 0.0029
72	0.0042 ± 0.0007	0.0030 ± 0.0029	0.0033 ± 0.0029	0.0034 ± 0.0029
73	0.0045 ± 0.0007	0.0032 ± 0.0028	0.0036 ± 0.0029	0.0037 ± 0.0029
74	0.0048 ± 0.0007	0.0035 ± 0.0028	0.0039 ± 0.0029	0.0040 ± 0.0029
75	0.0051 ± 0.0007	0.0037 ± 0.0028	0.0042 ± 0.0030	0.0042 ± 0.0029
76	0.0054 ± 0.0007	0.0040 ± 0.0028	0.0045 ± 0.0030	0.0045 ± 0.0029
77	0.0057 ± 0.0007	0.0043 ± 0.0027	0.0049 ± 0.0030	0.0048 ± 0.0029
78	0.0060 ± 0.0007	0.0046 ± 0.0027	0.0052 ± 0.0030	0.0051 ± 0.0029
79	0.0063 ± 0.0008	0.0049 ± 0.0027	0.0056 ± 0.0030	0.0054 ± 0.0029
80	0.0067 ± 0.0008	0.0052 ± 0.0027	0.0059 ± 0.0030	0.0058 ± 0.0029
81	0.0070 ± 0.0009	0.0055 ± 0.0026	0.0063 ± 0.0030	0.0061 ± 0.0029
82	0.0073 ± 0.0009	0.0058 ± 0.0026	0.0066 ± 0.0030	0.0064 ± 0.0029
83	0.0077 ± 0.0010	0.0061 ± 0.0026	0.0070 ± 0.0030	0.0067 ± 0.0029
84	0.0080 ± 0.0011	0.0064 ± 0.0026	0.0074 ± 0.0030	0.0070 ± 0.0029
85	0.0084 ± 0.0012	0.0067 ± 0.0026	0.0078 ± 0.0030	0.0074 ± 0.0029
86	0.0087 ± 0.0013	0.0070 ± 0.0027	0.0081 ± 0.0031	0.0077 ± 0.0029
87	0.0091 ± 0.0014	0.0074 ± 0.0027	0.0085 ± 0.0031	0.0080 ± 0.0029
88	0.0094 ± 0.0015	0.0077 ± 0.0027	0.0089 ± 0.0031	0.0083 ± 0.0029
89	0.0098 ± 0.0016	0.0080 ± 0.0028	0.0093 ± 0.0032	0.0087 ± 0.0030
90	0.0101 ± 0.0017	0.0083 ± 0.0029	0.0096 ± 0.0032	0.0090 ± 0.0030
91	0.0105 ± 0.0018	0.0086 ± 0.0029	0.0100 ± 0.0032	0.0093 ± 0.0030
92	0.0108 ± 0.0019	0.0089 ± 0.0030	0.0104 ± 0.0033	0.0096 ± 0.0030

93	0.0112 ± 0.0021	0.0092 ± 0.0031	0.0108 ± 0.0033	0.0100 ± 0.0031
94	0.0116 ± 0.0022	0.0095 ± 0.0032	0.0111 ± 0.0034	0.0103 ± 0.0031
95	0.0119 ± 0.0023	0.0099 ± 0.0032	0.0115 ± 0.0034	0.0106 ± 0.0031
96	0.0123 ± 0.0024	0.0102 ± 0.0033	0.0119 ± 0.0035	0.0110 ± 0.0032
97	0.0126 ± 0.0025	0.0105 ± 0.0034	0.0123 ± 0.0035	0.0113 ± 0.0032
98	0.0130 ± 0.0026	0.0108 ± 0.0035	0.0127 ± 0.0036	0.0116 ± 0.0032
99	0.0133 ± 0.0027	0.0111 ± 0.0036	0.0130 ± 0.0037	0.0119 ± 0.0033
100	0.0137 ± 0.0028	0.0114 ± 0.0038	0.0134 ± 0.0037	0.0123 ± 0.0033

Tab. S8 Full normalized knee flexion moment data during squatting under four knee protector conditions (group mean ± SD, n = 5).

Percent cycle (%)	Pro.off	Pro.a	Pro.b	Pro.c
0	-0.0002 ± 0.0002	-0.0002 ± 0.0002	-0.0001 ± 0.0001	-0.0001 ± 0.0004
1	-0.0002 ± 0.0001	-0.0002 ± 0.0002	-0.0000 ± 0.0001	-0.0000 ± 0.0004
2	-0.0001 ± 0.0001	-0.0002 ± 0.0002	-0.0000 ± 0.0001	-0.0000 ± 0.0004
3	-0.0001 ± 0.0001	-0.0001 ± 0.0002	0.0000 ± 0.0002	-0.0000 ± 0.0004
4	-0.0001 ± 0.0001	-0.0001 ± 0.0002	0.0000 ± 0.0002	0.0000 ± 0.0004
5	-0.0001 ± 0.0001	-0.0001 ± 0.0002	0.0001 ± 0.0002	0.0000 ± 0.0004
6	-0.0000 ± 0.0000	-0.0000 ± 0.0002	0.0001 ± 0.0002	0.0001 ± 0.0004
7	-0.0000 ± 0.0000	-0.0000 ± 0.0002	0.0001 ± 0.0002	0.0001 ± 0.0004
8	0.0000 ± 0.0000	0.0000 ± 0.0002	0.0001 ± 0.0002	0.0001 ± 0.0004
9	0.0000 ± 0.0000	0.0000 ± 0.0003	0.0002 ± 0.0003	0.0001 ± 0.0004
10	0.0001 ± 0.0001	0.0001 ± 0.0003	0.0002 ± 0.0003	0.0001 ± 0.0004
11	0.0001 ± 0.0001	0.0001 ± 0.0003	0.0002 ± 0.0003	0.0002 ± 0.0004
12	0.0001 ± 0.0001	0.0001 ± 0.0003	0.0003 ± 0.0003	0.0002 ± 0.0004
13	0.0002 ± 0.0001	0.0002 ± 0.0003	0.0003 ± 0.0003	0.0002 ± 0.0004
14	0.0002 ± 0.0001	0.0002 ± 0.0004	0.0003 ± 0.0004	0.0002 ± 0.0004
15	0.0003 ± 0.0001	0.0003 ± 0.0004	0.0004 ± 0.0004	0.0003 ± 0.0004
16	0.0003 ± 0.0002	0.0003 ± 0.0004	0.0004 ± 0.0004	0.0003 ± 0.0003
17	0.0003 ± 0.0002	0.0003 ± 0.0004	0.0004 ± 0.0004	0.0003 ± 0.0003
18	0.0004 ± 0.0002	0.0004 ± 0.0005	0.0005 ± 0.0005	0.0003 ± 0.0003
19	0.0004 ± 0.0002	0.0004 ± 0.0005	0.0005 ± 0.0005	0.0004 ± 0.0003
20	0.0005 ± 0.0003	0.0005 ± 0.0005	0.0005 ± 0.0005	0.0004 ± 0.0003
21	0.0005 ± 0.0003	0.0005 ± 0.0005	0.0006 ± 0.0005	0.0004 ± 0.0003
22	0.0006 ± 0.0003	0.0005 ± 0.0005	0.0006 ± 0.0006	0.0005 ± 0.0003
23	0.0006 ± 0.0003	0.0006 ± 0.0005	0.0007 ± 0.0006	0.0005 ± 0.0003
24	0.0007 ± 0.0004	0.0006 ± 0.0006	0.0007 ± 0.0006	0.0006 ± 0.0003
25	0.0007 ± 0.0004	0.0007 ± 0.0006	0.0007 ± 0.0006	0.0006 ± 0.0003
26	0.0008 ± 0.0004	0.0008 ± 0.0006	0.0008 ± 0.0006	0.0007 ± 0.0003
27	0.0008 ± 0.0004	0.0008 ± 0.0006	0.0008 ± 0.0007	0.0007 ± 0.0003
28	0.0009 ± 0.0004	0.0009 ± 0.0006	0.0009 ± 0.0007	0.0008 ± 0.0003
29	0.0010 ± 0.0005	0.0009 ± 0.0006	0.0009 ± 0.0007	0.0008 ± 0.0003
30	0.0010 ± 0.0005	0.0010 ± 0.0006	0.0010 ± 0.0007	0.0009 ± 0.0003
31	0.0011 ± 0.0005	0.0010 ± 0.0006	0.0011 ± 0.0007	0.0009 ± 0.0003
32	0.0012 ± 0.0005	0.0011 ± 0.0006	0.0011 ± 0.0007	0.0010 ± 0.0003
33	0.0012 ± 0.0006	0.0011 ± 0.0007	0.0012 ± 0.0008	0.0010 ± 0.0003
34	0.0013 ± 0.0006	0.0012 ± 0.0007	0.0012 ± 0.0008	0.0011 ± 0.0003
35	0.0014 ± 0.0006	0.0013 ± 0.0007	0.0013 ± 0.0008	0.0012 ± 0.0003
36	0.0014 ± 0.0006	0.0013 ± 0.0007	0.0013 ± 0.0008	0.0012 ± 0.0003

37	0.0015 ± 0.0006	0.0014 ± 0.0007	0.0014 ± 0.0008	0.0013 ± 0.0003
38	0.0016 ± 0.0006	0.0014 ± 0.0007	0.0014 ± 0.0008	0.0013 ± 0.0003
39	0.0016 ± 0.0006	0.0015 ± 0.0007	0.0015 ± 0.0008	0.0014 ± 0.0003
40	0.0017 ± 0.0006	0.0016 ± 0.0007	0.0016 ± 0.0008	0.0015 ± 0.0003
41	0.0018 ± 0.0007	0.0016 ± 0.0007	0.0016 ± 0.0008	0.0015 ± 0.0003
42	0.0019 ± 0.0007	0.0017 ± 0.0007	0.0017 ± 0.0008	0.0016 ± 0.0003
43	0.0019 ± 0.0007	0.0017 ± 0.0007	0.0017 ± 0.0008	0.0016 ± 0.0003
44	0.0020 ± 0.0007	0.0018 ± 0.0007	0.0018 ± 0.0008	0.0017 ± 0.0003
45	0.0021 ± 0.0007	0.0019 ± 0.0007	0.0018 ± 0.0008	0.0018 ± 0.0003
46	0.0021 ± 0.0007	0.0019 ± 0.0007	0.0019 ± 0.0008	0.0018 ± 0.0003
47	0.0022 ± 0.0007	0.0020 ± 0.0007	0.0020 ± 0.0008	0.0019 ± 0.0003
48	0.0023 ± 0.0007	0.0020 ± 0.0007	0.0020 ± 0.0008	0.0019 ± 0.0003
49	0.0023 ± 0.0007	0.0021 ± 0.0006	0.0021 ± 0.0008	0.0020 ± 0.0003
50	0.0024 ± 0.0007	0.0021 ± 0.0006	0.0021 ± 0.0008	0.0020 ± 0.0004
51	0.0024 ± 0.0007	0.0022 ± 0.0006	0.0022 ± 0.0008	0.0021 ± 0.0004
52	0.0025 ± 0.0007	0.0022 ± 0.0006	0.0022 ± 0.0008	0.0022 ± 0.0004
53	0.0026 ± 0.0007	0.0023 ± 0.0006	0.0023 ± 0.0008	0.0022 ± 0.0004
54	0.0026 ± 0.0007	0.0023 ± 0.0006	0.0023 ± 0.0008	0.0023 ± 0.0004
55	0.0027 ± 0.0007	0.0024 ± 0.0006	0.0024 ± 0.0008	0.0023 ± 0.0004
56	0.0027 ± 0.0007	0.0024 ± 0.0006	0.0024 ± 0.0008	0.0024 ± 0.0004
57	0.0028 ± 0.0006	0.0025 ± 0.0006	0.0025 ± 0.0008	0.0024 ± 0.0004
58	0.0028 ± 0.0006	0.0025 ± 0.0006	0.0025 ± 0.0008	0.0025 ± 0.0004
59	0.0029 ± 0.0006	0.0026 ± 0.0006	0.0026 ± 0.0008	0.0025 ± 0.0004
60	0.0029 ± 0.0006	0.0026 ± 0.0005	0.0026 ± 0.0008	0.0026 ± 0.0004
61	0.0030 ± 0.0006	0.0027 ± 0.0005	0.0027 ± 0.0008	0.0026 ± 0.0004
62	0.0030 ± 0.0006	0.0027 ± 0.0005	0.0027 ± 0.0007	0.0026 ± 0.0005
63	0.0031 ± 0.0006	0.0028 ± 0.0005	0.0028 ± 0.0007	0.0027 ± 0.0005
64	0.0031 ± 0.0006	0.0028 ± 0.0005	0.0028 ± 0.0007	0.0027 ± 0.0005
65	0.0032 ± 0.0006	0.0028 ± 0.0005	0.0029 ± 0.0007	0.0028 ± 0.0005
66	0.0032 ± 0.0006	0.0029 ± 0.0005	0.0029 ± 0.0007	0.0028 ± 0.0005
67	0.0033 ± 0.0006	0.0029 ± 0.0005	0.0030 ± 0.0007	0.0029 ± 0.0005
68	0.0033 ± 0.0006	0.0030 ± 0.0004	0.0030 ± 0.0007	0.0029 ± 0.0005
69	0.0033 ± 0.0006	0.0030 ± 0.0004	0.0030 ± 0.0007	0.0030 ± 0.0005
70	0.0034 ± 0.0006	0.0030 ± 0.0004	0.0031 ± 0.0007	0.0030 ± 0.0005
71	0.0034 ± 0.0005	0.0031 ± 0.0004	0.0031 ± 0.0007	0.0030 ± 0.0005
72	0.0035 ± 0.0005	0.0031 ± 0.0004	0.0032 ± 0.0007	0.0031 ± 0.0005
73	0.0035 ± 0.0005	0.0031 ± 0.0004	0.0032 ± 0.0007	0.0031 ± 0.0005
74	0.0035 ± 0.0005	0.0032 ± 0.0004	0.0032 ± 0.0007	0.0031 ± 0.0005
75	0.0036 ± 0.0005	0.0032 ± 0.0004	0.0033 ± 0.0007	0.0032 ± 0.0005
76	0.0036 ± 0.0005	0.0032 ± 0.0003	0.0033 ± 0.0007	0.0032 ± 0.0005
77	0.0036 ± 0.0005	0.0032 ± 0.0003	0.0033 ± 0.0007	0.0032 ± 0.0005
78	0.0037 ± 0.0005	0.0033 ± 0.0003	0.0034 ± 0.0007	0.0033 ± 0.0005
79	0.0037 ± 0.0005	0.0033 ± 0.0003	0.0034 ± 0.0006	0.0033 ± 0.0006
80	0.0037 ± 0.0005	0.0033 ± 0.0003	0.0034 ± 0.0006	0.0033 ± 0.0006
81	0.0038 ± 0.0005	0.0033 ± 0.0003	0.0035 ± 0.0006	0.0033 ± 0.0006
82	0.0038 ± 0.0005	0.0034 ± 0.0003	0.0035 ± 0.0006	0.0034 ± 0.0006
83	0.0038 ± 0.0005	0.0034 ± 0.0003	0.0035 ± 0.0006	0.0034 ± 0.0006
84	0.0038 ± 0.0005	0.0034 ± 0.0003	0.0035 ± 0.0006	0.0034 ± 0.0006
85	0.0039 ± 0.0005	0.0034 ± 0.0003	0.0036 ± 0.0006	0.0034 ± 0.0006
86	0.0039 ± 0.0004	0.0035 ± 0.0002	0.0036 ± 0.0006	0.0035 ± 0.0006
87	0.0039 ± 0.0004	0.0035 ± 0.0002	0.0036 ± 0.0006	0.0035 ± 0.0006

88	0.0039 ± 0.0004	0.0035 ± 0.0002	0.0036 ± 0.0006	0.0035 ± 0.0006
89	0.0039 ± 0.0004	0.0035 ± 0.0002	0.0037 ± 0.0006	0.0035 ± 0.0006
90	0.0040 ± 0.0004	0.0035 ± 0.0002	0.0037 ± 0.0006	0.0035 ± 0.0006
91	0.0040 ± 0.0004	0.0035 ± 0.0002	0.0037 ± 0.0006	0.0035 ± 0.0006
92	0.0040 ± 0.0004	0.0036 ± 0.0002	0.0037 ± 0.0006	0.0036 ± 0.0006
93	0.0040 ± 0.0004	0.0036 ± 0.0002	0.0037 ± 0.0006	0.0036 ± 0.0006
94	0.0040 ± 0.0004	0.0036 ± 0.0002	0.0038 ± 0.0006	0.0036 ± 0.0006
95	0.0041 ± 0.0004	0.0036 ± 0.0002	0.0038 ± 0.0006	0.0036 ± 0.0006
96	0.0041 ± 0.0004	0.0036 ± 0.0002	0.0038 ± 0.0007	0.0036 ± 0.0006
97	0.0041 ± 0.0004	0.0036 ± 0.0002	0.0038 ± 0.0007	0.0036 ± 0.0006
98	0.0041 ± 0.0004	0.0036 ± 0.0002	0.0039 ± 0.0007	0.0036 ± 0.0006
99	0.0041 ± 0.0004	0.0037 ± 0.0002	0.0039 ± 0.0007	0.0037 ± 0.0007
100	0.0041 ± 0.0004	0.0037 ± 0.0002	0.0039 ± 0.0007	0.0037 ± 0.0007

Tab. S9 Full normalized knee flexion moment data during STS under four knee protector conditions (group mean ± SD, n = 5).

Percent cycle (%)	Pro.off	Pro.a	Pro.b	Pro.c
0	0.0004 ± 0.0011	0.0000 ± 0.0001	0.0001 ± 0.0003	0.0002 ± 0.0005
1	0.0005 ± 0.0011	0.0001 ± 0.0001	0.0002 ± 0.0003	0.0002 ± 0.0005
2	0.0005 ± 0.0011	0.0001 ± 0.0001	0.0002 ± 0.0004	0.0003 ± 0.0006
3	0.0005 ± 0.0012	0.0001 ± 0.0001	0.0002 ± 0.0005	0.0003 ± 0.0007
4	0.0005 ± 0.0012	0.0002 ± 0.0002	0.0003 ± 0.0005	0.0004 ± 0.0007
5	0.0006 ± 0.0012	0.0002 ± 0.0002	0.0003 ± 0.0006	0.0004 ± 0.0008
6	0.0006 ± 0.0012	0.0002 ± 0.0003	0.0003 ± 0.0007	0.0004 ± 0.0008
7	0.0006 ± 0.0012	0.0003 ± 0.0004	0.0004 ± 0.0007	0.0005 ± 0.0009
8	0.0006 ± 0.0012	0.0003 ± 0.0004	0.0004 ± 0.0008	0.0005 ± 0.0009
9	0.0006 ± 0.0012	0.0003 ± 0.0005	0.0005 ± 0.0008	0.0006 ± 0.0009
10	0.0006 ± 0.0012	0.0004 ± 0.0006	0.0005 ± 0.0009	0.0006 ± 0.0010
11	0.0007 ± 0.0012	0.0004 ± 0.0006	0.0005 ± 0.0009	0.0006 ± 0.0010
12	0.0007 ± 0.0012	0.0005 ± 0.0007	0.0006 ± 0.0010	0.0007 ± 0.0010
13	0.0007 ± 0.0012	0.0005 ± 0.0008	0.0006 ± 0.0010	0.0007 ± 0.0010
14	0.0007 ± 0.0012	0.0006 ± 0.0008	0.0007 ± 0.0010	0.0008 ± 0.0010
15	0.0007 ± 0.0012	0.0007 ± 0.0009	0.0007 ± 0.0010	0.0008 ± 0.0010
16	0.0007 ± 0.0012	0.0007 ± 0.0009	0.0007 ± 0.0010	0.0009 ± 0.0010
17	0.0008 ± 0.0012	0.0008 ± 0.0009	0.0008 ± 0.0010	0.0009 ± 0.0010
18	0.0008 ± 0.0011	0.0008 ± 0.0009	0.0008 ± 0.0010	0.0009 ± 0.0010
19	0.0008 ± 0.0011	0.0009 ± 0.0010	0.0009 ± 0.0010	0.0010 ± 0.0010
20	0.0009 ± 0.0011	0.0010 ± 0.0010	0.0009 ± 0.0009	0.0010 ± 0.0009
21	0.0009 ± 0.0011	0.0010 ± 0.0010	0.0010 ± 0.0009	0.0011 ± 0.0009
22	0.0010 ± 0.0010	0.0011 ± 0.0009	0.0010 ± 0.0008	0.0012 ± 0.0009
23	0.0010 ± 0.0010	0.0012 ± 0.0009	0.0011 ± 0.0008	0.0012 ± 0.0008

24	0.0011 ± 0.0010	0.0012 ± 0.0009	0.0011 ± 0.0007	0.0013 ± 0.0008
25	0.0011 ± 0.0010	0.0013 ± 0.0009	0.0012 ± 0.0007	0.0013 ± 0.0008
26	0.0012 ± 0.0009	0.0014 ± 0.0009	0.0012 ± 0.0006	0.0014 ± 0.0008
27	0.0013 ± 0.0009	0.0014 ± 0.0008	0.0013 ± 0.0006	0.0014 ± 0.0007
28	0.0013 ± 0.0009	0.0015 ± 0.0008	0.0014 ± 0.0006	0.0015 ± 0.0007
29	0.0014 ± 0.0008	0.0016 ± 0.0008	0.0014 ± 0.0005	0.0016 ± 0.0007
30	0.0015 ± 0.0008	0.0016 ± 0.0007	0.0015 ± 0.0005	0.0016 ± 0.0007
31	0.0015 ± 0.0008	0.0017 ± 0.0007	0.0016 ± 0.0005	0.0017 ± 0.0007
32	0.0016 ± 0.0008	0.0017 ± 0.0006	0.0016 ± 0.0005	0.0018 ± 0.0007
33	0.0017 ± 0.0007	0.0018 ± 0.0006	0.0017 ± 0.0005	0.0018 ± 0.0007
34	0.0017 ± 0.0007	0.0018 ± 0.0006	0.0018 ± 0.0006	0.0019 ± 0.0007
35	0.0018 ± 0.0007	0.0019 ± 0.0005	0.0018 ± 0.0006	0.0019 ± 0.0007
36	0.0019 ± 0.0007	0.0020 ± 0.0005	0.0019 ± 0.0006	0.0020 ± 0.0007
37	0.0019 ± 0.0007	0.0020 ± 0.0005	0.0019 ± 0.0006	0.0021 ± 0.0007
38	0.0020 ± 0.0007	0.0021 ± 0.0005	0.0020 ± 0.0006	0.0021 ± 0.0007
39	0.0021 ± 0.0007	0.0021 ± 0.0005	0.0021 ± 0.0006	0.0022 ± 0.0007
40	0.0021 ± 0.0007	0.0022 ± 0.0005	0.0021 ± 0.0006	0.0022 ± 0.0007
41	0.0022 ± 0.0007	0.0022 ± 0.0005	0.0022 ± 0.0007	0.0023 ± 0.0007
42	0.0022 ± 0.0007	0.0023 ± 0.0005	0.0022 ± 0.0007	0.0023 ± 0.0007
43	0.0023 ± 0.0007	0.0023 ± 0.0005	0.0023 ± 0.0007	0.0024 ± 0.0007
44	0.0024 ± 0.0007	0.0024 ± 0.0005	0.0023 ± 0.0007	0.0024 ± 0.0007
45	0.0024 ± 0.0007	0.0024 ± 0.0005	0.0024 ± 0.0007	0.0024 ± 0.0007
46	0.0025 ± 0.0008	0.0024 ± 0.0006	0.0024 ± 0.0007	0.0025 ± 0.0007
47	0.0025 ± 0.0008	0.0025 ± 0.0006	0.0024 ± 0.0007	0.0025 ± 0.0007
48	0.0025 ± 0.0008	0.0025 ± 0.0006	0.0025 ± 0.0007	0.0025 ± 0.0007
49	0.0026 ± 0.0008	0.0025 ± 0.0006	0.0025 ± 0.0007	0.0025 ± 0.0007
50	0.0026 ± 0.0008	0.0025 ± 0.0006	0.0025 ± 0.0007	0.0026 ± 0.0007
51	0.0027 ± 0.0008	0.0025 ± 0.0006	0.0025 ± 0.0007	0.0026 ± 0.0007
52	0.0027 ± 0.0008	0.0026 ± 0.0006	0.0025 ± 0.0007	0.0026 ± 0.0007
53	0.0027 ± 0.0008	0.0026 ± 0.0006	0.0026 ± 0.0007	0.0026 ± 0.0007
54	0.0027 ± 0.0008	0.0026 ± 0.0007	0.0026 ± 0.0007	0.0026 ± 0.0007
55	0.0027 ± 0.0008	0.0026 ± 0.0007	0.0026 ± 0.0007	0.0026 ± 0.0007
56	0.0028 ± 0.0008	0.0026 ± 0.0007	0.0026 ± 0.0007	0.0026 ± 0.0007
57	0.0028 ± 0.0008	0.0026 ± 0.0007	0.0026 ± 0.0007	0.0026 ± 0.0007
58	0.0028 ± 0.0008	0.0026 ± 0.0007	0.0026 ± 0.0007	0.0026 ± 0.0007
59	0.0028 ± 0.0008	0.0025 ± 0.0007	0.0026 ± 0.0007	0.0026 ± 0.0007
60	0.0028 ± 0.0008	0.0025 ± 0.0007	0.0026 ± 0.0007	0.0026 ± 0.0008
61	0.0028 ± 0.0008	0.0025 ± 0.0007	0.0026 ± 0.0007	0.0026 ± 0.0008
62	0.0028 ± 0.0008	0.0025 ± 0.0007	0.0026 ± 0.0007	0.0026 ± 0.0008
63	0.0028 ± 0.0008	0.0025 ± 0.0007	0.0026 ± 0.0007	0.0026 ± 0.0008

64	0.0028 ± 0.0008	0.0025 ± 0.0007	0.0025 ± 0.0007	0.0026 ± 0.0008
65	0.0028 ± 0.0008	0.0024 ± 0.0007	0.0025 ± 0.0007	0.0026 ± 0.0008
66	0.0028 ± 0.0008	0.0024 ± 0.0007	0.0025 ± 0.0007	0.0026 ± 0.0008
67	0.0028 ± 0.0008	0.0024 ± 0.0007	0.0025 ± 0.0007	0.0026 ± 0.0008
68	0.0028 ± 0.0008	0.0024 ± 0.0007	0.0025 ± 0.0007	0.0026 ± 0.0008
69	0.0028 ± 0.0008	0.0024 ± 0.0007	0.0025 ± 0.0007	0.0026 ± 0.0008
70	0.0028 ± 0.0008	0.0023 ± 0.0007	0.0025 ± 0.0007	0.0026 ± 0.0008
71	0.0028 ± 0.0008	0.0023 ± 0.0007	0.0025 ± 0.0007	0.0026 ± 0.0008
72	0.0028 ± 0.0008	0.0023 ± 0.0007	0.0025 ± 0.0007	0.0025 ± 0.0008
73	0.0028 ± 0.0008	0.0023 ± 0.0007	0.0024 ± 0.0007	0.0025 ± 0.0008
74	0.0027 ± 0.0008	0.0023 ± 0.0007	0.0024 ± 0.0007	0.0025 ± 0.0008
75	0.0027 ± 0.0007	0.0023 ± 0.0007	0.0024 ± 0.0007	0.0025 ± 0.0008
76	0.0027 ± 0.0007	0.0022 ± 0.0008	0.0024 ± 0.0007	0.0024 ± 0.0008
77	0.0027 ± 0.0007	0.0022 ± 0.0008	0.0023 ± 0.0007	0.0024 ± 0.0008
78	0.0026 ± 0.0007	0.0022 ± 0.0008	0.0023 ± 0.0006	0.0024 ± 0.0008
79	0.0026 ± 0.0007	0.0022 ± 0.0008	0.0023 ± 0.0007	0.0023 ± 0.0008
80	0.0026 ± 0.0007	0.0022 ± 0.0008	0.0022 ± 0.0007	0.0023 ± 0.0008
81	0.0025 ± 0.0007	0.0022 ± 0.0008	0.0022 ± 0.0007	0.0023 ± 0.0008
82	0.0025 ± 0.0007	0.0021 ± 0.0008	0.0021 ± 0.0007	0.0022 ± 0.0008
83	0.0024 ± 0.0007	0.0021 ± 0.0008	0.0021 ± 0.0007	0.0022 ± 0.0008
84	0.0024 ± 0.0007	0.0021 ± 0.0008	0.0020 ± 0.0008	0.0021 ± 0.0009
85	0.0024 ± 0.0007	0.0021 ± 0.0008	0.0020 ± 0.0008	0.0021 ± 0.0009
86	0.0023 ± 0.0008	0.0020 ± 0.0008	0.0019 ± 0.0009	0.0020 ± 0.0009
87	0.0023 ± 0.0008	0.0020 ± 0.0008	0.0019 ± 0.0009	0.0020 ± 0.0010
88	0.0022 ± 0.0008	0.0020 ± 0.0008	0.0019 ± 0.0010	0.0019 ± 0.0010
89	0.0022 ± 0.0009	0.0019 ± 0.0008	0.0018 ± 0.0010	0.0019 ± 0.0011
90	0.0021 ± 0.0009	0.0019 ± 0.0008	0.0018 ± 0.0011	0.0019 ± 0.0011
91	0.0021 ± 0.0010	0.0018 ± 0.0008	0.0018 ± 0.0011	0.0018 ± 0.0012
92	0.0021 ± 0.0010	0.0018 ± 0.0009	0.0018 ± 0.0012	0.0018 ± 0.0012
93	0.0020 ± 0.0011	0.0017 ± 0.0009	0.0017 ± 0.0012	0.0018 ± 0.0013
94	0.0020 ± 0.0011	0.0017 ± 0.0010	0.0017 ± 0.0012	0.0018 ± 0.0013
95	0.0020 ± 0.0012	0.0016 ± 0.0010	0.0017 ± 0.0013	0.0017 ± 0.0013
96	0.0019 ± 0.0012	0.0016 ± 0.0011	0.0017 ± 0.0013	0.0017 ± 0.0014
97	0.0019 ± 0.0013	0.0015 ± 0.0012	0.0017 ± 0.0013	0.0017 ± 0.0014
98	0.0019 ± 0.0013	0.0015 ± 0.0013	0.0016 ± 0.0013	0.0017 ± 0.0014
99	0.0018 ± 0.0014	0.0015 ± 0.0013	0.0016 ± 0.0014	0.0017 ± 0.0015
100	0.0018 ± 0.0014	0.0014 ± 0.0014	0.0016 ± 0.0014	0.0017 ± 0.0015
